# Seafood and Neurocognitive Development in Children: A Systematic Review

**DOI:** 10.1016/j.advnut.2025.100391

**Published:** 2025-02-14

**Authors:** Lauren E O’Connor, Maureen K Spill, Sanjoy Saha, Arin Balalian, Julie S Davis, Amanda J MacFarlane

**Affiliations:** Texas A&M Agriculture, Food and Nutrition Evidence Center, Fort Worth, TX, United States

**Keywords:** fish, shellfish, ω-3 fatty acids, fatty fish, infants and toddlers, developmental disorders

## Abstract

Seafood is a source of essential nutrients to support neurocognitive development of children and adolescents, but there are concerns about contaminant exposure. Assessing seafood as a food group, rather than a source of nutrients or contaminants, can inform future dietary guidance. This study aimed to update and assess relationships between seafood consumption during childhood and adolescence and neurocognitive development. Three electronic databases were searched until September 2024 to update a previous search from 2000 to 2019. Articles were included if associations were assessed between seafood intake during childhood and adolescence and neurocognitive development outcomes (cognitive development, social-emotional and behavioral development, movement/physical development, language/communication development, depression, anxiety, attention-deficit/hyperactivity disorder, and autism spectrum disorder). All articles were screened at title, abstract, and full-text levels by 2 independent analysts. Data were extracted by 1 analyst, quality checked by a second analyst, and synthesized narratively by 2 analysts independently, considering direction, magnitude, and statistical significance of results for each outcome; discrepancies were resolved via discussion. Risk of bias was assessed using ROBINS-E and ROB 2.0. Certainty of evidence was assessed with Grading of Recommendations Assessment, Development and Evaluation (GRADE). Eighteen articles from 5 short-term (12–16 wk) RCTs conducted in Northern Europe and 9 prospective cohort studies conducted in various countries were included. The evidence suggested a relationship between higher seafood consumption and improved cognitive development outcomes for children and adolescents aged 0–18 y old (GRADE: low). This conclusion was informed by 5 short-term RCTs in which children aged 10 mo to 15 y were provided fatty fish compared with meat, poultry, or fish oil supplements. These RCTs were largely supported by results from 5 longer-term prospective cohort studies. Evidence was inconsistent for social-emotional and behavioral development outcomes and was lacking for other outcomes. Seafood consumption within current recommended intake amounts consumed mainly as fatty fish likely improves cognitive development outcomes in children and adolescents.

This review was registered at PROSPERO as CRD42023432844.

**Statement of significance**

Consuming seafood within current recommended intake amounts as mainly fatty fish likely improves cognitive development outcomes in children and adolescents. Seafood intake for all individuals in the United States, including children and adolescents, is below current recommendations, thus increasing intake may support better neurocognitive developmental outcomes.

## Introduction

Seafood, defined as fish and shellfish [1], is the primary dietary source of long chain polyunsaturated fatty acids (i.e., DHA and EPA) that are essential for child neurocognitive development [2]. Seafood recommendations are limited to 2–3 oz/wk for infants and toddlers and 8–10 oz/wk for children and adolescents in the United States [1], partly due to concerns about exposure to metals (eg, methylmercury and arsenic) [3] and other contaminants (eg, perfluoroalkyl and polyfluoroalkyl substances) [4,5]. This concern stems from delayed development and neurocognitive abnormalities observed in populations with atypical seafood intake patterns (eg, pilot whale) [6,7] or abnormally high contaminant exposures (eg, environmental disasters that polluted waters) [8,9]. Seafood intake may be less concerning for populations that consume a variety of seafood (eg, ocean compared with freshwater sources and imported compared with exported) or are under different environmental or biological conditions (eg, adequate selenium status) [10,11]. More research is needed to understand if beneficial effects of seafood intake outweigh risks of potential contaminant exposures and if higher seafood intakes would support improved neurocognitive developmental outcomes for children and adolescents in the United States.

The 2020 Dietary Guidelines Advisory Committee (DGAC) conducted a systematic review to assess relationships between seafood intake during childhood and adolescents and neurocognitive development [12] that was used as one resource to inform the 2020–2025 Dietary Guidelines for Americans [1]. Overall, the DGAC concluded that there was insufficient evidence to make conclusions [12]. In 2022, the United States Food and Drug Administration in collaboration with the Environmental Protection Agency, Department of Agriculture, and the National Oceanic and Atmospheric Administration tasked the National Academies of Sciences, Engineering, and Medicine (NASEM) to convene a committee to reassess the state of scientific evidence in nutrition and toxicology on this topic. This included a resynthesis of the evidence from the DGAC report on seafood intake and neurocognitive development in children and adolescents with new evidence published since the DGAC database search concluded [13]. Therefore, in support of this NASEM committee, we conducted a systematic review to provide an updated assessment of relationships between seafood consumption during childhood and adolescence and neurocognitive development.

## Methods

This systematic review was designed to update the 2020 DGAC systematic review [12]. As tasked by NASEM [13], the DGAC database search was updated to identify new studies and the data extraction, risk of bias assessments, and data synthesis were performed for all studies, including the studies included in the original DGAC review. This was deemed necessary to ensure that all data were extracted consistently and to allow for the use of an updated risk of bias tool. Our protocol was registered a priori in PROSPERO (CRD42023432844) and was based on the 2020 DGAC protocol [12]. Our protocol included the review questions, general search strategy, inclusion/exclusion criteria, risk of bias assessment, and synthesis plan including heterogeneity investigation. Our reporting adhered to the PRISMA guidelines [14] (Supplemental Appendix 1) and our methods met specifications of a high-quality systematic review according to the AMSTAR 2 critical appraisal tool [15] (Supplemental Appendix 2).

In brief, randomized controlled trials (RCTs), nonrandomized intervention studies, prospective cohort studies (PCSs), and retrospective cohort studies that compared different types, amounts, sources, frequency, or timing of seafood consumption during childhood and adolescence and neurocognitive development outcomes in the child at ages 0–18 y old were eligible (Supplemental Figure 1). Neurocognitive outcomes assessed included cognitive development, social-emotional and behavioral development (referred to as ‘behavior’ throughout the article), movement/physical development, language/communication development, depression, anxiety, attention-deficit/hyperactivity disorder (ADHD), and autism spectrum disorder (Supplemental Table 1). These study designs and outcomes were selected to reflect the 2020 DGAC protocol that was developed by technical experts in the field and systematic review methodologists.

### Search strategy

This systematic review included articles identified in the previous DGAC search from January 2000 to October 2019 and our updated replicated literature search until September 6, 2024. The full search strategy is shown in Supplemental Table 2.

### Screening

Two independent analysts screened articles at the title, abstract, and full-text levels using DistillerSR (Evidence Partners; 2020), following the same protocol as the DGAC review. Analysts piloted the screening forms with ≥25 articles to ensure the forms were adequate and that analysts interpreted the eligibility criteria similarly. Title screening was used to exclude clearly irrelevant studies; any disagreements automatically moved onto the next level. Disagreements about whether to include or exclude an article at the abstract or full-text level were discussed and resolved by 2 analysts. If necessary, a third analyst was consulted to resolve differences. Backward citation searching was conducted manually by reviewing the reference lists of all included articles. The list of inclusion and exclusion criteria used for screening is shown in Supplemental Table 3.

### Data extraction

Data from all articles were extracted by a trained analyst using a systematic approach and a standardized data extraction form. A second analyst reviewed all extracted data for accuracy and completeness. Any suggested changes were discussed. If necessary, a third analyst was consulted. The following data were extracted, as available, from each article: study characteristics including author name, publication year, study design, study name, country, baseline sample size, and funding source; participant characteristics including mother’s age, child sex (% female), race/ethnicity, socioeconomic status, maternal anthropometrics, gestational weight gain, and infant feeding practices; exposure details including definition/description of seafood intake, assessment method, seafood consumption amount and type, child levels of nutrients from seafood including ω-3 (n–3) polyunsaturated fatty acids, iodine, selenium, iron, fish protein, and vitamin D and maternal/infant levels of mercury; confounders including key confounders accounted for, key confounders not accounted for, and other confounders accounted for; outcome(s) and results including outcome category (Supplemental Table 1), outcome assessment tool, outcome assessment methods including subscale, child age at outcome assessment, results, analytical sample size, study limitations, summary of results, and quantified data as needed for synthesis. Data were extracted as is; unclear or missing data are noted throughout the article and tables. Authors were not contacted for missing data.

### Risk of bias

Risk of bias was assessed for all included articles independently by 2 analysts using one of the following tools depending on study design: ROB 2.0 for RCTs [16], ROBINS-I for nonrandomized studies of interventions [17], and ROBINS-E for nonrandomized studies of exposures [18]. These tools were designed to assess risk of bias by domain and then determine an overall risk of bias rating for each included article. The analysts piloted the tools on 2 to 3 articles to ensure a consistent approach and interpretation was applied. Further, upon completion of the dual, independent risk-of-bias assessments, domain-level ratings were compared between the 2 analysts. If there were differences, the analysts discussed and determined the appropriate rating. If necessary, a third analyst was consulted. The domain that had the highest risk of bias score was identified, and that score was applied as the overall rating for that article.

### Data synthesis

Articles were grouped for synthesis by outcome category, followed by study design, and then organized by age group described in the primary studies. Results were described using the study name because there were often multiple articles per study. Results reported in the primary articles were narratively synthesized by 2 analysts independently, considering direction, strength, and magnitude of reported effects or associations. Discrepancies were discussed until consensus was reached. Study characteristics and outcome data were presented in tabular format. Details of each assessment tool and guidance for interpreting the results are available in column T “Assessment tool interpretation” in Supplemental Data Appendix. Sensitivity analyses were conducted by omitting studies that were at high or very high risk of bias. Additional sources of heterogeneity, such as seafood type or population characteristics, were considered during the narrative synthesis.

Meta-analyses were planned, as indicated in the protocol, but not performed due to limitations in the data. This was a deviation from the protocol. This decision was made because only a small portion of the total extracted data could be pooled (eg, 8 of the 98 cognitive development outcomes and 10 of 32 behavior outcomes) due to the variation in the population age, outcome assessment tools, suboutcomes and the variety of estimands reported.

### Certainty of evidence

For each conclusion, GRADE was used to assess certainty of the evidence [19]. GRADE considers risk of bias, inconsistency, indirectness, and imprecision in results of included articles as well as risk for publication bias. For observational study designs, there are additional considerations related to dose–response relationships, magnitude of effect, and residual confounding. RCTs and nonrandomized studies of exposure (i.e., PCSs) were assessed separately, and the overall certainty rating was determined by the study design with the highest certainty.

## Results

### Search results

**FIGURE 1:**
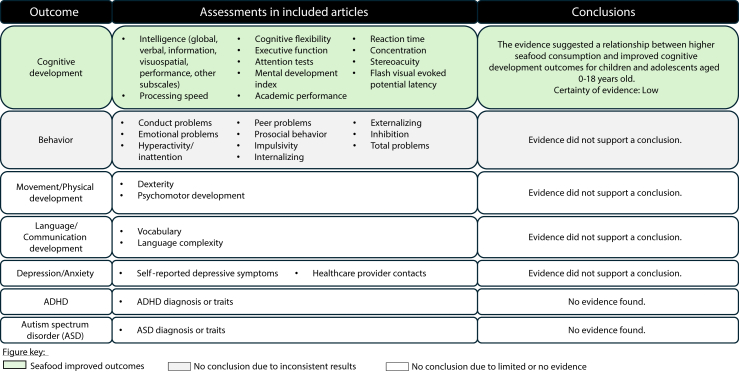
Summary of conclusions for relationship between seafood intake during childhood and adolescence and neurocognitive development outcomes. Behavior includes social-emotional and behavioral development. Certainty of evidence is further described in Table 3.

Five articles were identified from the updated search [[20], [21], [22], [23], [24]] (Supplemental Figure 2), resulting in 18 included articles from 5 RCTs and 9 PCSs (Table 1) [[20], [21], [22], [23], [24], [25], [26], [27], [28], [29], [30], [31], [32], [33], [34], [35], [36], [37]]. Full-text articles that were reviewed and excluded are listed in Supplemental Table 4. Results for each outcome are described in the text and tables and summarized in Figure 1.

**TABLE 1:** Characteristics of studies assessing relationships between seafood consumption during childhood and adolescence and neurocognitive development.

Study	Sample characteristics^1^	Seafood intervention	Comparator intervention(s)	Dietary compliance	Outcomes	Funding source
RCTs (parallel-arm design, *n* = 5)						
Polyunsaturated fatty acids in child nutrition (PINGU) [23]	10 mo old^2^, Germany, *n* = 214	Study-provided jarred infant foods; 2 meals/wk as vegetable-potato-salmon meals; intervention started at 5–7 mo old until 10 mo old	Comparator 1 (Rapeseed group): study-provided jarred infant foods with ALA-rich rapeseed oil; 2 meals/wk are vegetable-potato-meat meals; intervention started at 5–7 mo old until 10 mo old Comparator 2 (Corn oil control group): study-provided jarred infant foods with LA-rich corn oil; 2 meals/wk are vegetable-potato-meat meals; intervention started at 5–7 mo old until 10 mo old	Calculated for 1 wk at 6 and 9 mo based on available self-reported dietary records (details of tool not described). ≥1 fish meal/wk (in fish group only): At 6 mo: 76.5% At 9 mo: 89.7%	Cognition (Bayley Scales of Infant Development II, Flash visual evoked potential latency); Movement/physical (Bayley Scales of Infant Development II)	Federal Ministry of Education and Research, Module Innovations and New Ideas for the Nutritional Sector, HiPP^3^
Unnamed RCT [25]	5.0 ± 0.8 y old (mean ± SD), Germany, *n* = 205	3 meals provided/wk for 16 wk; family choice of prepared meals containing 50 g Atlantic salmon	3 meals provided to the entire family/wk for 16 wk; family choice of prepared meals containing 50 g beef, turkey, or ham meat	Compliance: Daily food diary during intervention; parent report of whether child ate a study meal, which one, how much was consumed, and other seafood meals eaten	Cognition (Wechsler Preschool and Primary Scale of Intelligence, third edition); Movement/physical (9-Hole Peg Test)	European Research Council
Fish Intervention Studies–KIDS (FINS-KIDS) [[26], [27], [28]]	5.2 ± 0.6 y old (mean ± SD), Norway, *n* = 232	3 prepared warm lunch meals provided/wk for 16 wk containing 50–80 g fatty fish (herring and mackerel)	3 prepared warm lunch meals provided/wk for 16 wk containing 50–80 g meat (chicken, lamb, and beef)	Weigh-backs performed by research staff during school lunch time. Total mean (SD) Fish intake for fish group: 2070 (978) g Meat intake for meat group: 2675 (850) g	Behavior^5^ (Strengths and Difficulties Questionnaire); Cognition (Wechsler Preschool and Primary Scale of Intelligence, third edition); Movement/physical (9-Hole Peg Test)	Norwegian Seafood Research Fund, Pelagia^3^
FiSK Junior study (Fish, children, health, and cognition) [24]	9.6 (9.2–9.7) (median and IQR), Denmark, *n* = 199	2 fish dinners provided/wk (salmon fillets) and 3 fish lunches/wk (salmon fish cakes, mackerel in tomato sauce, smoked mackerel, marinated herring, smoked trout, salmon sausages); ∼300 g/wk for 12 ± 2 wk	2 poultry dinners provided/wk (organic chicken: minced, whole, breast, or thigh), and 3 poultry lunches/wk (chicken liver pate, chicken meatballs, turkey salami, chicken sausages); ∼300 g/wk for 12 ± 2 wk	Median oily fish intake was 37 (19–61) g/wk; increased to 375 (325–426) g/wk during the intervention for fish group measured via FFQ (details of tool not described). Erythrocyte EPA+DHA FA%: increase from 4.9 ± 1.0 to 7.3 ± 1.4 in the fish group, which was 2.3 (95% CI: 1.9, 2.6) higher than poultry group at follow-up, indicating good compliance	Behavior (Behavior Rating Inventory of Executive Function, d2 Test of Attention, Flanker Test, KINDLC Questionnaire of Quality of Life, Cambridge Neurophychological Automated Battery, Strengths and Difficulties Questionnaire, Stroop Color Word Test); Cognition (Behavior Rating Inventory of Executive Function, Cambridge Neuropsychological Automated Battery, d2 Test of Attention, Flanker Test, Stroop Color Word Test, Switch Test)	Nordea-fonden, Skagenfood,^4^ Sødam,^3^ REMA1000 Danmark,^3^ Amanda Seafoods^3^
Fish Intervention Studies–TEENS (FINS-TEENS) [29,30]	14.6 ± 0.3 y old (mean ± SD), Norway, *n* = 478	3 prepared school meals provided/wk for 12 wk with 90 g fatty fish (salmon, mackerel, herring)	Comparator 1: 3 prepared school meals provided/wk for 12 wk with 90 g meat/cheese (chicken, turkey, beef, ± cheese) Comparator 2: habitual school lunch plus provided fish oil supplements 3 times/wk for 12 wk with the equivalent LCPUFAs as 90 g fish	Capsules counted and estimated food consumed was estimated by eye based on one-fourth servings by research staff during school lunch time. Compliance (% participants who consumed at least half of the meals/capsules during the trial), monitored by trained research assistants: Fish group: 38% Meat group: 66% Supplement group: 87%	Behavior (Stengths and Difficulties Questionnaire); Cognition (d2 Test of Attention)	Norwegian Seafood Research Fund, Marine Harvest,^3^ Lerøy,^3^ Pelagia^3^
Prospective cohort studies (*n* = 9)						
Odense Child Cohort [21]	Baseline data collected at 18 mo old with follow-up at 21 and 30 mo old, Denmark, *n* = 2448	Higher frequency of fish intake (not defined)	Lower frequency of fish intake (not defined)	Single question in postnatal questionnaire	Language/communication (MacArthur Bates Communicative Development Inventories)	Novo Nordic Foundation, Danish Council for Independent Research, Medical Sciences, Human Biomonitoring for Europe, European Union Horizon 2020, Odense University Hospital, Royal Danish Library
Avon Longitudinal Study of Parents and Children (ALSPAC)^4^ [31,32]	Baseline data collected at 3 y old with ≥4 follow-ups at 4–13 y old, United Kingdom, *n* = 13,988	Servings per week of white fish, oily fish, other fish, and shellfish	No or lower servings per week of white fish, oily fish, other fish, and shellfish	FFQ, semiquantitative, developed and compared with intakes from Dietary and Nutritional Survey of British Adults [38]; intake reported by parents	Behavior (Strengths and Difficultities Questionnaire); Cognition (Stereoacuity Test)	Eunice Kennedy Shriver National Institute of Child Health, Human Development of the National Institutes of Health, and Economic and Social Research Council, The Medical Research Council, The Wellcome Trust, The Ministry of Agriculture, Foods and Fisheries, the Departments of Health and the Environment, The South West Regional Health Authority, the National Eye Research Centre, Cow and Gate, and Milupa^3^
Spanish Environment and Childhood Project (INMA) [22]	Baseline data collected at 5 y old with follow-up at 8 y old, Spain, *n* = 2644	Quintile 1 of grams of seafood (not defined) per week	Comparator 1: Quintile 2 of grams of seafood (not defined) per week Comparator 2: Quintile 3 of grams of seafood (not defined) per week Comparator 3: Quintile 4 of grams of seafood (not defined) per week Comparator 4: Quintile 5 of grams of seafood (not defined) per week	Semiquantitative FFQ, validity and reproducibility assessed for children aged 5 y [39]; reported not described but tool developed for parental report	Cognition (Attention Network Test)	Spanish Institute of Health Carlos III-Ministry of Economy and Competitiveness, Generalitat de Catalunya-CIRIT, Generalitat Valenciana, Alicia Koplowitz Foundation, Universidad de Oviedo, Fundación Cajastur-Liberbank, Department of Health of the Basque Government, the Provincial Government of Gipuzkoa, the Fundación Roger Torné, Instituto de Salud Carlos III, and Spanish Institute of Health Carlos III
China Jintan Child Cohort Study [33]	Baseline data collected at 9–11 y with follow-up at 12 y old, China, *n* = 1009	Never consumed fish (not defined)	Comparator 1: Sometimes consumed fish (not defined) Comparator 2: Often consumed fish (not defined)	Nonquantified intake frequency question, development and validation described; self-reported by child	Cognition (Wechsler Intelligence Scales for Children)	NIH, National Institute of Environmental Health Sciences, and National Institute on Alcohol Abuse and Alcoholism
Children’s Lifestyle and School Performance Study (CLASS) [34]	Baseline data collected at ∼10–11 y with follow-up through 13–14 y old, Canada, *n* = 5517	First tertile of servings per day of fish intake (not defined) over past 12 mo	Comparator 1: Second tertile of servings per day of fish intake (not defined) over past 12 mo Comparator 2: Third tertile of servings per day of fish intake (not defined) over past 12 mo	Harvard Youth/Adolescent FFQ based on the validated Nurses’ Health Study FFQ, reproducibility in children assessed [40]; self-reported by child	Depression (number of health care contacts for internalizing disorder over 3-y period)	Canada Foundation for Innovation Leaders Opportunity Fund, Canadian Population Health Initiative, Canadian Institutes for Health Research, The Heart and Stroke Foundation of Canada
ROOTS Study [35]	Baseline data collected at 14.5 ± 0.3 y old with follow-up at 17 y old, United Kingdom, *n* = 1238	Fish (not defined) servings per week	Different amount of fish (not defined) servings per week	4-d diet diary (2 weekdays, 2 weekend days) with estimated portion size; average daily fish intake converted to servings per day using serving size of 140 g; validation not described; training provided, reported by child	Depression (Moods and Feelings Questionnaire)	Wellcome Trust, National Institute for Health and Care Research, Collaboration for Leadership in Applied Research and Care East of England, Medical Research Council, British Heart Foundation, Cancer Research UK, Economic and Social Research Council, Royal Society
ALLERGY 2000 [36]	Baseline data collected at 15 y old with follow-up at 16 y old, Sweden, *n* = 18,158	<1 servings of fish (not defined) intake per week	Comparator 1: 1 serving of fish (not defined) per week Comparator 2: >1 serving of fish (not defined) per week	Questionnaire assessing frequency of meals containing fish, development and validation not described; self-reported by child	Cognition (academic performance measured via total grades and high school entrance criterion)	Wellcome Trust, National Institute for Health and Care Research, Collaboration for Leadership in Applied Research and Care East of England, Medical Research Council, British Heart Foundation, Cancer Research UK, Economic and Social Research Council, Royal Society
Unnamed prospective cohort study [37]	Baseline data collected at 15 y old with follow up at 18 y old, Sweden, *n* = 4792	<1 serving of fish (not defined other than fish-containing meals) per week	Comparator 1: 1 serving of fish (not defined other than fish-containing meals) per week Comparator 2: >1 serving of fish (not defined other than fish-containing meals) per week	Questionnaire, no details provided, validation not described; completed by children with their parents	Cognition (intelligence test from the Swedish military service conscription examination)	Swedish Society of Medicine, Department of Public Health at the Vastra Gotaland Region, Swedish Science Council
Community Empowerment and Care for Wellbeing and Health Longevity [20]	Age not reported but assessed outcomes across a span of 6 y, Japan, *n* = 185	Fish and seafood consumption	Different amount of fish and seafood consumption	Nonquantified FFQ, nonquantified, development and validation not described; reported not described	Behavior (Strengths and Difficulties Questionnaire)	Grants-in-Aid for Scientific Research

Abbreviations: FFQ, food frequency questionnaire.

1 Baseline sample size. Analytical sample size may vary based on outcome category.

2 Age at outcome assessment.

3 Names of for-profit entities.

4 The name of this trial varies. It is formerly known as the Avon Longitudinal Study of Pregnancy and Childhood but will be referred to as the Avon Longitudinal Study of Parents and Children for consistency.

5 Behavior includes social-emotional and behavioral development

### Cognitive development

There were 5 RCTs [[23], [24], [25], [26], [27],^29^] and 5 PCSs [22,31,33,36,37] that assessed relationships between seafood intake and cognitive development (Table 2). The RCTs were short-term (<16 wk), conducted in Northern Europe, assessed outcomes in children aged 10 mo to 15 y, and compared fatty fish intake to meat, poultry, or ω-3 fatty acid supplements. The RCTs were either at low risk of bias [24,26,29], had some concerns due to randomization or intervention deviations [25,27], or at high risk of bias due to reporting [23] (Supplemental Table 5). The 5 PCSs were conducted in the United Kingdom, Spain, and China with 1 each and 2 studies in Sweden. Baseline dietary assessment occurred between the ages of 3 and 15 y, and outcomes were measured after 1–3 y of follow-up. Three assessed fish intake (not further defined), whereas 2 assessed seafood intake more generally [22,31]. The PCSs had some concerns of bias [33,36] due to confounding, exposure measurement, and missing data or were at high risk of bias [22,31,37] due to reporting (Supplemental Table 5).

**TABLE 2:** Relationships between seafood consumption during childhood and adolescence and cognitive development outcomes.

Study name	Outcome assessment tool	Results	
Parallel-arm RCTs (*n* = 5), organized from youngest to oldest age at outcome assessment			
Polyunsaturated fatty acids in child nutrition (PINGU) [23]	Bayley Scales of Infant Development II	Mean (SD) at 10 mo: Mental Development Index Rapeseed oil group (*n* = 40): 99.1 (9.3) Salmon group (*n* = 39): 98.7 (10.9) Corn oil control group (*n* = 45): 96.8 (8.8) Group difference: *P* = 0.78	
	Flash visual evoked potential latency measured in milliseconds	Mean (SD) at 10 mo: Mean left + right eye: Rapeseed oil group (*n* = 47): 111.5 (13.0) Salmon group (*n* = 41): 111.9 (12.6) Corn oil control group (*n* = 46): 117.9 (18.1) Group difference: *P* = 0.07 Left eye: Rapeseed oil group (*n* = 47): 112.3 (15.7) Salmon group (*n* = 41): 111.1 (12.6) Corn oil control group (*n* = 45): 118.7 (18.7) Group difference: *P* = 0.03 Right eye: Rapeseed oil group (*n* = 44): 111.3 (11.0) Salmon group (*n* = 40): 113.2 (13.0) Corn oil control group (*n* = 46): 117.6 (18.2) Group difference: *P* = 0.14	
Unnamed trial [25]	German version of the Wechsler Preschool and Primary Scale of Intelligence, third edition	Preintervention to postintervention change at age 4–6 y, mean (95% CI): Global intelligence full scale Salmon group (*n* = 96): 1.2 (0.6, 3.1) Meat (beef, turkey, or ham) group (*n* = 93): 1.0 (−0.2, 2.2) Group difference: *P* = 0.334 Global intelligence raw score Salmon group (*n* = 96): 17.4 (14.8, 20.1) Meat group^1^ (*n* = 93): 14.6 (11.9, 17.3) Group difference: *P* = 0.143 Verbal intelligence subscale Salmon group (*n* = 96): −0.4 (−1.8, 1.0) Meat group^1^ (*n* = 93): −0.3 (−1.6, 1.1) Group difference: *P* = 0.923 Verbal intelligence subtest Salmon group (*n* = 96): 2.4 (1.5, 3.4) Meat group^1^ (*n* = 93): 1.9 (0.9, 2.9) Group difference: *P* = 0.444 Performance intelligence subscale Salmon group (*n* = 96): 3.5 (1.8, 5.2) Meat group^1^ (*n* = 93): 1.4 (−0.3, 3.1) Group difference: *P* = 0.082 Performance intelligence subtest Salmon group (*n* = 96): 5.0 (3.8, 6.2) Meat group^1^ (*n* = 93): 3.2 (2.1, 4.4) Group difference: *P* = 0.039 Processing speed subscale Salmon group (*n* = 96): 3.4 (1.3, 5.6) Meat group^1^ (*n* = 93): 3.3 (1.1, 5.5) Group difference: *P* = 0.934 Processing speed subtest Salmon group (*n* = 96): 10.1 (7.9, 12.3) Meat group^1^ (*n* = 93): 9.4 (7.1, 11.6) Group difference: *P* = 0.640	Preintervention to postintervention change at age 4–6 y, mean (95% CI): Information test Salmon group (*n* = 96): 1.1 (0.6, 1.5) Meat group^1^ (*n* = 93): 0.6 (0.2, 1.0) Group difference: *P* = 0.142 Vocabulary test Salmon group (*n* = 96): 0.9 (0.2, 1.6) Meat group^1^ (*n* = 93): 0.4 (−0.3, 1.1) Group difference: *P* = 0.329 Word reasoning test Salmon group (*n* = 96): 0.6 (0.1, 1.0) Meat group^1^ (*n* = 93): 0.8 (0.4, 1.3) Group difference: *P* = 0.407 Block design test Salmon group (*n* = 96): 2.3 (1.4, 3.2) Meat group^1^ (*n* = 93): 1.5 (0.6, 2.4) Group difference: *P* = 0.222 Matrix reasoning test Salmon group (*n* = 96): 1.1 (0.7, 1.6) Meat group^1^ (*n* = 93): 1.0 (0.6, 1.5) Group difference: *P* = 0.718 Picture concept test Salmon group (*n* = 96): 1.5 (1.0, 2.1) Meat group^1^ (*n* = 93): 0.7 (0.1, 1.3) Group difference: *P* = 0.038 Coding test Salmon group (*n* = 96): 5.2 (3.3, 7.0) Meat group^1^ (*n* = 93): 5.4 (3.6, 7.3) Group difference: *P* = 0.833 Symbol search test Salmon group (*n* = 96): 5.0 (4.1, 6.0) Meat group^1^ (*n* = 93): 3.6 (2.6, 4.6) Group difference: *P* = 0.047
Fish Intervention Studies–KIDS (FINS-KIDS) [26,27]	Norwegian version of the Wechsler Preschool and Primary Scale of Intelligence, third edition [27]	Postintervention scores (95% CI) at age 4–6 y: Full scale intelligence Fish (herring and mackerel) group (*n* = 101): 162.6 (156.5, 168.6) Meat (chicken, lamb, and beef) group (*n* = 109): 160.0 (154.1, 165.9) Group difference: *P* = 0.475 Verbal intelligence subscale Fish group^2^ (*n* = 101): 60.2 (57.9, 62.5) Meat group^3^ (*n* = 109): 60.1 (57.8, 62.3) Group difference: *P* = 0.914	Postintervention scores (95% CI) at age 4–6 y: Performance intelligence subscale Fish group^2^ (*n* = 101): 56.4 (54.9, 57.9) Meat group^3^ (*n* = 109): 56.4 (55.0, 57.8) Group difference: *P* = 0.973 Processing speed subscale Fish group^2^ (*n* = 101): 45.1 (42.8, 47.4) Meat group^3^ (*n* = 109): 44.3 (42.1, 46.5) Group difference: *P* = 0.613
	Norwegian version of the Wechsler Preschool and Primary Scale of Intelligence, third edition [26]	Preintervention to postintervention change at 4–6 y, mean (95% CI): Total raw score Fish group^2^ (*n* = 105): 17.7 (14.8, 20.7) Meat group^3^ (*n* = 113): 17.8 (15.0, 20.6) Group difference: *P* = 0.97 (Per-protocol analysis found a significantly larger increase in fish group than in meat group, *P* = 0.006) Verbal raw score Fish group^2^ (*n* = 105): 3.8 (2.6, 5.0) Meat group^3^ (*n* = 113): 4.3 (3.1, 5.4) Group difference: *P* = 0.59 (Per-protocol analysis also showed NS) Performance raw score Fish group^2^ (*n* = 105): 6.0 (4.7, 7.3) Meat group^3^ (*n* = 113): 5.6 (4.4, 6.8) Group difference: *P* = 0.65 (Per-protocol analysis also showed NS) Processing speed raw score Fish group^2^ (*n* = 105): 8.1 (5.9, 10.3) Meat group^3^ (*n* = 113): 7.8 (5.7, 9.9) Group difference: *P* = 0.83 (Per-protocol analysis also showed NS) Information subtest Fish group^2^ (*n* = 105): 1.0 (0.6, 1.4) Meat group^3^ (*n* = 113): 1.1 (0.8, 1.5) Group difference: *P* = 0.63 (Per-protocol analysis also showed NS) Vocabulary subtest Fish group^2^ (*n* = 105): 1.1 (0.3, 1.9) Meat group^3^ (*n* = 113): 1.1 (0.4, 1.9) Group difference: *P* = 0.99 (Per-protocol analysis found a significantly larger increase in fish group than in meat group, *P* = 0.0468)	Preintervention to postintervention change at 4–6 y, mean (95% CI): Matrix reasoning subtest Fish group^2^ (*n* = 105): 2.5 (1.8, 3.1) Meat group^3^ (*n* = 113): 2.2 (1.6, 3.1) Group difference: *P* = 0.52 (Per-protocol analysis also showed NS) Picture concept subtest Fish group^2^ (*n* = 105): 2.1 (1.1, 3.0) Meat group^3^ (*n* = 113): 2.0 (1.1, 2.9) Group difference: *P* = 0.91 (Per-protocol analysis also showed NS) Coding subtest Fish group^2^ (*n* = 105): 4.5 (2.9, 6.2) Meat group^3^ (*n* = 113): 5.2 (3.6, 6.8) Group difference: *P* = 0.58 (Per-protocol analysis also showed NS) Symbol search subtest Fish group^2^ (*n* = 105): 3.6 (2.7, 4.5) Meat group^3^ (*n* = 113): 2.6 (1.7, 3.5) Group difference: *P* = 0.12 (Per-protocol analysis found a significantly larger increase in fish group than in meat group, *P* = 0.0163) Word reasoning subtest Fish group^2^ (*n* = 105): 1.8 (1.1, 2.4) Meat group^3^ (*n* = 113): 2.1 (1.4, 2.7) Group difference: *P* = 0.50 (Per-protocol analysis also showed NS) Block design subtest Fish group^2^ (*n* = 105): 1.7 (1.3, 2.1) Meat group^3^ (*n* = 113): 1.1 (0.7, 1.6) Group difference: *P* = 0.068 (Per-protocol analysis found a significantly larger increase in fish group than in meat group, *P* = 0.0269)
FiSK Junior study (Fish, children, health, and cognition) [24]	Behavior Rating Inventory of Executive Function (BRIEF)	Difference in preintervention to postintervention change between fish (salmon, mackerel, herring, and trout; *n* = 98) and poultry (chicken and turkey; *n* = 98) groups at ∼10 y, mean (95% CI): BRIEF global executive function: −1.51 (−4.45, 1.43), *P* = 0.310 BRIEF flexibility: 0.20 (−0.32, 0.72), *P* = 0.446 BRIEF working memory: −0.29 (−0.95, 0.37), *P* = 0.385	
	Cambridge Neurophychological Automated Battery	Difference in preintervention to postintervention change between fish^4^ (*n* = 98–99) and poultry^5^ (*n* = 98) groups at ∼10 y, OR (95% CI): Short-term memory, PAL memory score (%): 1.15 (0.92, 1.44), *P* = 0.214 Working memory, SWM strategy score: 0.35 (−0.21, 0.92), *P* = 0.219 Processing speed, 5-choice reaction time median (ms): −3 (−12, 6), *P* = 0.526 Processing speed, 5-choice reaction time SD (ms): 2 (−6, 11), *P* = 0.621 Rapid visual processing total error (%): 0.88 (0.79, 0.98), *P* = 0.017 Rapid visual processing misses (%): 0.87 (0.75, 1.02), *P* = 0.089	
	Flanker Test	Difference in preintervention to postintervention change between fish^4^ (*n* = 97) and poultry^5^ (*n* = 92) groups at ∼10 y, mean (95% CI): Cognitive flexibility, mixing cost (ms): −51 (−94, −7), *P* = 0.024 Flanker total error (%): 0.90 (0.65, 1.25), *P* = 0.520	
	Principal component analysis from a battery of tests	Difference in preintervention to postintervention change between fish^4^ (*n* = 89) and poultry^5^ (*n* = 89) groups at ∼10 y, mean (95% CI): Overall cognitive performance: −0.17 (−0.35, 0.01), *P* = 0.060 Speed-accuracy trade-off: 0.02 (−0.22, 0.27), *P* = 0.844	
	Switch Test	Difference in preintervention to postintervention change between fish^4^ (*n* = 97) and poultry^5^ (*n* = 92) groups at ∼10 y, mean (95% CI): Cognitive flexibility, Switch cost (ms): −5 (−43, 32), *P* = 0.776 Processing speed, switch reaction time (ms): −39 (−83, 6), *P* = 0.086 Switch total error (%): 0.97 (0.86, 1.09), *P* = 0.572	
	d2 Test of Attention	Difference in preintervention to postintervention change between fish^4^ (*n* = 99) and poultry^5^ (*n* = 97) groups at ∼10 y, mean (95% CI): Processing speed (characters): 2.5 (−4.7, 9.7), *P* = 0.490 Inattention error (%): 1.11 (0.93, 1.33), *P* = 0.239	
	Stroop Color-Word Test	Difference in preintervention to postintervention change between fish^4^ (*n* = 93) and poultry^5^ (*n* = 95) groups at ∼10 y, mean (95% CI): Processing speed, Stroop color time (s): −2 (−5, 1), *P* = 0.166	
Fish Intervention Studies–TEENS (FINS-TEENS) [29]	d2 Test of Attention	Preintervention to postintervention change at 14–15 y, β (95% CI): Processing speed (total number of characters processed) Fish (salmon, mackerel, and herring) group (*n* = 137): 1, REF Meat/cheese (chicken, turkey, and beef, with or without cheese) group (*n* = 148): −11.8 (−23.3, −0.4), *P* = 0.042 Supplement (fish oil) group (*n* = 141): −13.4 (−24.9, −1.8), *P* = 0.024 (Per-protocol analysis found lower significance for meat and supplement groups) Concentration performance (total number of correctly cancelled out targets minus commission errors) Fish group^6^ (*n* = 137): 1, REF Meat group^7^ (*n* = 148): −2.3 (−6.8, 2.2), *P* = 0.317 Supplement group^8^ (*n* = 141): −2.4 (−6.9, 2.2), *P* = 0.306 (Per-protocol analysis showed NS for meat and supplement groups) Total performance (total number of characters processed minus total errors made) Fish group^6^ (*n* = 137): 1, REF Meat group^7^ (*n* = 148): −7.9 (−17.4, 1.6), *P* = 0.103 Supplement group^8^ (*n* = 141): −10.4 (−20.0, −0.7), *P* = 0.035 (Per-protocol analysis showed NS for meat group and lower significance for supplement group) Omission errors (unmarked target characters) Fish group^6^ (*n* = 137): 1, REF Meat group^7^ (*n* = 148): 0.85 (0.74, 0.98), *P* = 0.026 Supplement group^8^ (*n* = 141): 1.01 (0.83, 1.23), *P* = 0.933 (Per-protocol analysis found NS for meat and supplement groups) Commission errors (incorrectly marked distraction characters) Fish group^6^ (*n* = 137): 1, REF Meat group^7^ (*n* = 148): 0.91 (0.59, 1.39), *P* = 0.648 Supplement group^8^ (*n* = 141): 0.88 (0.63, 1.24), *P* = 0.469 (Per-protocol analysis found NS for meat and supplement groups) Total errors (sum of omission and commission errors) Fish group^6^ (*n* = 137): 1, REF Meat group^7^ (*n* = 148): 0.88 (0.75, 1.02), *P* = 0.094 Supplement group^8^ (*n* = 141): 0.96 (0.80, 1.15), *P* = 0.671 (Per-protocol analysis found NS for meat and supplement groups)	
Prospective cohort studies (*n* = 5), organized from youngest to oldest age at outcome assessment			
Avon Longitudinal Study of Parents and Children (ALSPAC) [31]	Stereoacuity test	Association between child white fish, oily fish, other fish, and shellfish intake^9^ and stereoacuity at 3.5 y: Foveal stereo (*n* = 150) No: 33.3% Yes: 38.2% Macular stereo (*n* = 236) No: 50.7% Yes: 54.5% Peripheral stereo (*n* = 57) No: 16.3% Yes: 7.3% Univariate χ^2^, *P* = 0.039	
Spanish Environment and Childhood Project (INMA) [22]	Attention Network Test	Association between seafood^10^ intake at 5 y and omission errors at 8 y, IRR (95% CI): Q1 (median 84 g/wk, *n* = 281): REF Q2 (median 162 g/wk, *n* = 289): 0.92 (0.74, 1.14) Q3 (median 213 g/wk, *n* = 307): 0.88 (0.70, 1.10) Q4 (median 271 g/wk, *n* = 312): 0.92 (0.74, 1.16) Q5 (median 377 g/wk, *n* = 301): 1.03 (0.82, 1.30) *P*-trend = 0.646	
China Jintan Child Cohort Study [33]	Wechsler Intelligence Scales for Children, Chinese version	Association between fish^10^ intake at age 9–11 and intelligence at 12 y, β (SE): Global intelligence Never or seldom fish intake (*n* = 89): REF Sometimes fish intake (*n* = 315): 3.31 (1.45), *P* = 0.023 Often fish intake (*n* = 137): 4.80 (1.63), *P* = 0.003 Verbal intelligence Never or seldom fish intake (*n* = 89): REF Sometimes fish intake (*n* = 315): 2.92 (1.39), *P* = 0.036 Often fish intake (*n* = 137): 4.75 (1.55), *P* = 0.002 Performance intelligence Never or seldom fish intake (*n* = 89): REF Sometimes fish intake (*n* = 315): 2.52 (1.51), *P* = 0.097 Often fish intake (*n* = 137): 3.79 (1.69), *P* = 0.026	
ALLERGY 2000 [36]	School grades, total score; entrance criterion to senior high school in Sweden	Association between fish^10^ intake at 15 y and school grades at 16 y, β (95% CI), n = 9448: Fish intake < 1/wk: REF Fish intake 1/wk: 14.5 (11.8, 17.1), *P* < 0.0001 Fish intake > 1/wk: 19.9 (16.5, 23.3), *P* < 0.0001	
Unnamed prospective cohort study [37]	Intelligence test from the Swedish military service conscription examination	Association between fish^10^ intake at 15 y and intelligence at 18 y, β (95% CI), *n* = 3972: Global intelligence <1×/wk: REF 1×/wk: 0.36 (0.21, 0.51) >1×/wk: 0.58 (0.39, 0.77) Verbal intelligence <1×/wk: REF 1×/wk: 0.20 (0.05, 0.34) >1×/wk: 0.46 (0.29, 0.64) Visuospatial intelligence <1×/wk: REF 1×/wk: 0.33 (0.18, 0.48) >1×/wk: 0.51 (0.32, 0.69)	

Abbreviations: NS, nonsignificance; Q, quintile; REF, referent.

1 Meat group included self-selected beef, turkey, or ham.

2 Fish group included herring and mackerel.

3 Meat group included chicken, lamb, and beef.

4 Fish group included salmon fillets provided for dinner twice weekly. Salmon fish cakes, mackerel in tomato sauce, smoked mackerel, marinated herring, smoked trout, and salmon sausages were provided for lunch thrice weekly.

5 Poultry group included organic chicken (minced, whole, breast, or thigh) provided for dinner twice weekly. Chicken liver pate, chicken meatballs, turkey salami, and chicken sausages were provided for lunch thrice weekly.

6 Fish group included salmon, mackerel, and herring.

7 Meat/cheese group included chicken, turkey, and beef, with or without cheese.

8 Supplement group included fish oil.

9 Authors used the term fish, however, shellfish was included.

10 Authors used the term seafood or fish intake with no further description.

#### Evidence from RCTs

One RCT provided jarred food to 3 groups of infants that contained either salmon, rapeseed oil, or a corn oil control from 5–7 mo until 10 mo of age [23]. The infants who received salmon scored 2 points higher on the mental development index at 10 mo old than the control group, but 1 point lower than those who received rapeseed oil. These differences were not statistically significant, and all infants scored within a normal range. The reaction time, assessed via flash visual evoked potential latency, of infants who received salmon or rapeseed oil was 4–6 ms faster than the control group, which was statistically significant for 2 of 3 assessments.

For children aged 4–6 y, 2 RCTs—1 in Germany [25] and the Fish Intervention Studies (FINS)–KIDS [26,27] provided 2 groups of children with 150–240 g/wk (∼5–9 oz) of either fatty fish or meat/cheese for 16 wk and assessed intelligence with the Wechsler Preschool and Primary Scale of Intelligence. The fish group showed greater improvements in 81% (13/16) and 56% (9/16) of intelligence assessments in the unnamed RCT and FINS-KIDS, respectively. Moreover, the children who received fatty fish in FINS-KIDS had higher postintervention scores for 3 of 4 assessments. However, all differences in changes over time and postintervention values between groups were small (<3 points), and only 3 outcomes were statistically significant.

There were 2 studies on adolescents aged 10–15 y. The first was the FiSK Junior [24] in which 2 groups of adolescents received 300 g/wk (∼11 oz) of fatty fish or poultry for ∼12 wk. Cognitive development was measured via 3 different assessment tools. Across all assessments, 74% (14/19) suggested greater improvements in the fish group compared with the poultry group. However, differences between groups were small, and only 2 results were statistically significant. The second study was the FINS-TEENS [29] in which 3 groups received 270 g/wk (∼10 oz) of fatty fish, meat, or a fish oil supplement for 12 wk. The fish group had statistically greater improvements in processing speed than the meat and fish oil groups and statistically greater improvements in total performance than the fish oil group on the d2 test of attention. However, the effect sizes were small across all outcomes, with the highest difference being 13 on a scale that exceeds 400. Similarly, differences in attention errors were <1 point and were not statistically significant.

#### Evidence from PCSs

For children aged 3.5 y, results from the Avon Longitudinal Study of Parents and Children study suggested that higher intake of white fish, oily fish, other fish, and shellfish was statistically associated with higher likelihood of stereoacuity [31]. At 5 y old, in the Spanish Environment and Childhood Project study, seafood intake was not associated with attention outcomes at 8 y old [22]. For adolescents, evidence from 3 PCSs suggested that higher frequency of fish intake (not further defined) at ages 9–15 y was associated with higher intelligence test scores [33,37] and better academic performance [36] after 1–3 y of follow up.

#### Conclusion

Overall, the evidence suggested a relationship between higher seafood consumption, as mainly fatty fish, and improved cognitive development outcomes for children and adolescents aged 0–18 y old. There was consistency between short-term RCTs and longer-term PCSs in that the direction of results suggested benefits of higher fish intake. However, improvements within groups and differences between groups were small and largely not statistically significant. Conclusions did not change after excluding the 1 RCT and 3 PCSs that were at high risk of bias due to the overall consistency in results. The certainty of evidence was low (Table 3), due to lack of diversity in population characteristics and seafood type assessed as well as the imprecision in the results.

**TABLE 3:** Evidence suggested a relationship between higher seafood consumption and improved cognitive development outcomes for children and adolescents aged 0–18 y (GRADE: low).

Study design; No. of articles	Risk of bias^1^	Inconsistency^2^	Indirectness^2^	Imprecision^2^	Publication bias^3^	Large effect	Plausible confounding	Dose–response	Summary of findings	Certainty
5 RCTs from 6 articles [[23], [24], [25], [26], [27],^29^]	Not serious	Not serious	Serious; Population limited to Northern Europe; intervention only included fatty fish	Serious; wide CIs and large *P* values	Undetected	NA	NA	NA	Fatty fish consumption for 12–16 wk favorably affected cognitive development in children and adolescents aged 10 mo to 15 y compared with meat, poultry, or fish oil supplements	Low
NRS-Exp; 5 PCSs from 5 articles [22,31,33,36,37]	Serious; some concerns (*n* = 2) and high risk (*n* = 1)	Not serious	Not serious	Not serious	Strongly detected; all results statistically significant	No	No	No	Higher vs lower frequency of fish consumption was associated with favorable cognitive development at ages 3.5–18 y	Low

Certainty of evidence assessed using GRADE [19]: very low, low, moderate, or high.

Abbreviations: NA, not applicable; NRS-Exp, nonrandomized study of exposure; PCS, prospective cohort study; ROB, risk of bias.

1 Domain only downgrades. Rating choices: extremely serious, very serious, serious, or not serious.

2 Domain only downgrades. Rating choices: very serious, serious, or not serious.

3 Domain only downgrades. Rating choices: strongly detected, or undetected.

### Behavior

There were 3 RCTs [24,28,30] and 2 PCSs [20,32] that assessed relationships between seafood intake and behavior, including social-emotional and behavioral development (Table 4). The RCTs were short-term (<16 wk), conducted in Northern Europe, assessed outcomes in children aged 4 to 15 y and compared fatty fish intake to meat, meat/cheese, or poultry. The RCTs were at low risk of bias [24] or had some concerns [28,30] of bias due to missing data, outcome measurement, and selection of reported results (Supplemental Table 6). The PCSs were conducted in the United Kingdom and Japan and assessed intake of seafood or small fish and seaweed, respectively. Both these PCSs were at high risk of bias, due to confounding, missing data, and selection of reported results (Supplemental Table 6).

**TABLE 4:** Relationships between seafood consumption during childhood and adolescence and behavior outcomes.

Study name	Outcome assessment tool	Results	
Parallel-arm RCTs (*n* = 3), organized from youngest to oldest age at outcome assessment			
Fish Intervention Studies–KIDS (FINS-KIDS) [28]	Strengths and Difficulties Questionnaire	Preintervention to postintervention change at 4–6 y, mean (95% CI): Hyperactivity/inattention Fish (herring and mackerel) group (*n* = 81): 0.10 (−0.23, 0.42) Meat (chicken, lamb, and beef) group (*n* = 89): −0.03 (−0.35, 0.28) Group difference: *P* = 0.536 Emotional problems Fish^1^ group (*n* = 81): −0.02 (−0.29, 0.24) Meat^2^ group (*n* = 89): −0.08 (−0.33, 0.17) Group difference: *P* = 0.765 Conduct problems Fish^1^ group (*n* = 81): 0.04 (−0.22, 0.30) Meat^2^ group (*n* = 89): −0.07 (−0.32, 0.18) Group difference: *P* = 0.501	Preintervention to postintervention change at 4–6 y, mean (95% CI): Peer problems Fish^1^ group (*n* = 81): 0.07 (−0.15, 0.29) Meat^2^ group (*n* = 89): −0.16 (−0.37, 0.05) Group difference: *P* = 0.135 Total problems Fish^1^ group (*n* = 81): 0.22 (−0.47, 0.91) Meat^2^ group (*n* = 89): −0.37 (−1.03, 0.30) Group difference: *P* = 0.191 Per-protocol analysis for all outcomes also showed NS
FiSK Junior study (Fish, children, health, and cognition) [24]	Cambridge Neuropsychological Automated Battery	Difference in preintervention to postintervention change between fish (salmon, mackerel, herring, and trout) (*n* = 97) and poultry (chicken and turkey) (*n* = 98) groups at ∼10 y, OR (95% CI)—impulsivity, RVP false alarm (%): 0.86 (0.73, 1.02), *P* = 0.076	
	d2 Test of Attention	Difference in preintervention to postintervention change between fish^3^ (*n* = 99) and poultry^4^ (*n* = 97) groups at ∼10 y, OR (95% CI)—impulsivity error (%): 0.65 (0.41, 1.02), *P* = 0.062	
	Flanker Test	Difference in preintervention to postintervention change between fish^3^ (*n* = 97) and poultry^4^ (*n* = 92) groups at ∼10 y, OR (95% CI): Impulsivity, Flanker incongruent error (%): 0.99 (0.89, 1.09), *P* = 0.796 Inhibition, Flanker effect (ms): 2 (−11, 15), *P* = 0.773	
	Stroop Color-Word Test	Difference in preintervention to postintervention change between fish^3^ (*n* = 93) and poultry^4^ (*n* = 95) groups at ∼10 y, mean (95% CI)—inhibition, Stroop effect (s): −2 (−6, 3), *P* = 0.459	
	Behavior Rating Inventory of Executive Function	Difference in preintervention to postintervention change between fish^3^ (*n* = 98) and poultry^4^ (*n* = 98) groups at ∼10 y, mean (95% CI): Externalizing problems, impulsivity: −0.13 (−0.68, 0.42), *P* = 0.648 Internalizing problems, emotional control: −0.04 (−0.63, 0.55), *P* = 0.900	
	KINDLC Questionnaire of Quality of Life, child rated	Difference in preintervention to postintervention change between fish^3^ (*n* = 99) and poultry^4^ (*n* = 98) groups at ∼10 y, mean (95% CI): Internalizing problems, emotional well-being: 1.55 (−1.44, 4.54), *P* = 0.308 Prosocial behavior, friends: −0.03 (−3.61, 3.56), *P* = 0.989 Total problems, total well-being: −0.18 (−2.14, 1.78), *P* = 0.860	
	KINDLP Questionnaire of Quality of Life, parent rated	Difference in preintervention to postintervention change between fish^3^ (*n* = 99) and poultry^4^ (*n* = 98) groups at ∼10 y, mean (95% CI): Internalizing problems, KINDLP emotional well-being: 1.04 (−1.57, 3.65), *P* = 0.432 Prosocial behavior, friends: 0.43 (−2.20, 3.07), *P* = 0.745 Total problems, total well-being: 0.21 (−1.62, 2.04), *P* = 0.820	
	Principal component analysis from a battery of tests	Difference in preintervention to postintervention change between fish^3^ (*n* = 98) and poultry^4^ (*n* = 98) groups at ∼10 y, mean (95% CI): Overall socioemotional problems: −0.13 (−0.26, 0.01), *P* = 0.079 Externalizing vs internalizing problems: 0.02 (−0.20, 0.24), *P* = 0.865	
	Strengths and Difficulties Questionnaire	Difference in preintervention to postintervention change between fish^3^ (*n* = 99) and poultry^4^ (*n* = 98) groups at ∼10 y, mean (95% CI): Externalizing problems: −0.24 (−0.69, 0.21), *P* = 0.301 Internalizing problems: −0.63 (−1.11, −0.16), *P* = 0.009 Prosocial score: 0.17 (−0.12, 0.46), *P* = 0.240 Total difficulties: −0.89 (−1.60, −0.18), *P* = 0.014	
Fish Intervention Studies–TEENS (FINS-TEENS) [30]	Strengths and Difficulties Questionnaire, self-report for 11–16 y	Preintervention to postintervention change at 14–15 y, mean (95% CI): Prosocial behavior Fish (salmon, mackerel, and herring) group (*n* = 137): −0.02 (−0.25, 0.22), REF Meat (chicken, turkey, and beef, with or without cheese) group (*n* = 145): −0.03 (−0.26, 0.19), *P* = 0.93 Supplement (fish oil) group (*n* = 143): 0.04 (−0.19,0.26), *P* = 0.75 (Per-protocol analysis showed NS for meat and supplement groups) Hyperactivity/inattention Fish^5^ group (*n* = 137): −0.10 (−0.34, 0.16), REF Meat^6^ group (*n* = 145): 0.10 (−0.15, 0.35), *P* = 0.28 Supplement^7^ group (*n* = 143): −0.08 (−0.32, 0.17), *P* = 0.92 (Per-protocol analysis showed NS for meat and supplement groups) Conduct problems Fish^5^ group (*n* = 137): −0.07 (−0.27, 0.14), REF Meat^6^ group (*n* = 145): −0.27 (−0.47, −0.07), *P* = 0.13 Supplement^7^ group (*n* = 143): 0.10 (−0.10, 0.30), *P* = 0.23 (Per-protocol analysis showed NS for meat and supplement groups) Peer problems Fish^5^ group (*n* = 137): −0.02 (−0.22, 0.17), REF Meat^6^ group (*n* = 145): −0.16 (−0.35, 0.03), *P* = 0.31 Supplement^7^ group (*n* = 143): −0.02 (−0.21, 0.17), *P* = 0.99 (Per-protocol analysis showed NS for meat and supplement groups) Total difficulties Fish^5^ group (*n* = 137): −0.11 (−0.65, 0.44), REF Meat^6^ group (*n* = 145): −0.33 (−0.90, 0.20), *P* = 0.57 Supplement^7^ group (*n* = 143): 0.08 (−0.45, 0.62), *P* = 0.63 (Per-protocol analysis showed NS for meat and supplement groups)	
Prospective cohort studies (*n* = 2), organized from youngest to oldest age at outcome assessment			
Community Empowerment and Care for Wellbeing and Health Longevity [20]	Strength and Difficulties Questionnaire	Association between small fish and seaweed intake at 1–6 y and behavior problems (age NR): data not reported, NS	
Avon Longitudinal Study of Parents and Children (ALSPAC) [32]	Strengths and Difficulties Questionnaire	Mean (SE) weekly white fish, oily fish, other fish, and shellfish^8^ intake at 3 y by conduct problem trajectory: Severe conduct problems Boys, *n* = 348: 1.11 (0.08); Girls, *n* = 268: 1.25 (0.09) Low conduct problems Boys, *n* = 2312: 1.21 (0.03); Girls, *n* = 2420: 1.35 (0.03) Conduct problem trajectory: *P* = 0.12 Sex: *P* = 0.025	

Abbreviations: NR, not reported; NS, nonsignificance; OR, odds ratio; REF, referent.

1 Fish group included herring and mackerel.

2 Meat group included chicken, lamb, and beef.

3 Fish group included salmon fillets provided for dinner twice weekly. Salmon fish cakes, mackerel in tomato sauce, smoked mackerel, marinated herring, smoked trout, and salmon sausages were provided for lunch thrice weekly.

4 Poultry group included organic chicken (minced, whole, breast, or thigh) provided for dinner twice weekly. Chicken liver pate, chicken meatballs, turkey salami, and chicken sausages were provided for lunch thrice weekly.

5 Fish group included salmon, mackerel, and herring.

6 Meat/cheese group included chicken, turkey, and beef with or without cheese.

7 Supplement group included fish oil.

8 Authors used the term fish; however, shellfish was included.

#### Results from RCTs

For children aged 4–6 y, there was no change in behavior outcomes assessed with the Strengths and Difficulties Questionnaire within the fish or meat group in the FINS-KIDS study [28]. Outcomes changed from preintervention to postintervention within each group by <0.1 point during the 16-wk intervention, which were not statistically significant. Differences between groups were trivial, in part due to minor longitudinal chages, and not statistically significant, but the direction of effect consistently favored the meat compared to the fish group.

For adolescents aged 10–15 y, the FiSK Junior [24] and FINS-TEENS [30] studies assessed behavior using a variety of assessment tools. In the FiSK Junior study [24], participants in the fish group had greater improvements than the poultry group in 84% (16/19) of behavioral assessments. The largest improvement was 14%–36% lower odds of impulsive behaviors, but these results were not statistically significant. Additionally, those in the fish group compared with the poultry group had statistically significant greater improvements in internalizing problems and total difficulties assessed with the Strengths and Difficulties Questionnaire, but the difference in effect sizes were <1 point between groups. In FINS-TEENS [30], those in the fish group had higher postintervention scores on only 2 of the 5 of the Strengths and Difficulties Questionnaire components than those in the meat group. The other 3 favored the meat group but differences between groups were small (<0.5 points) and were not statistically significant.

#### Evidence from PCSs

Evidence from the 2 PCSs suggested that higher compared with lower parental-reported seafood intake at <6 y old was not statistically associated with behavior [20] or conduct [32] problems after ≤10 y follow-up. However, sufficient data were not provided to allow for an assessment of direction or magnitude.

#### Conclusions

Overall, it was unclear whether there was a relationship between higher seafood consumption and behavior outcomes in children and adolescents aged 0–18 y old. There was inconsistency in the direction of results from RCTs, as well as minimal differences both within and between fish and comparison groups. Additionally, both PCSs were at high risk of bias and neither reported results in a way that the direction and magnitude of association could be interpreted to aid conclusions.

### Movement/physical development

There were 3 RCTs (Table 5) that assessed relationships between seafood intake and movement/physical development—PINGU [23], FINS-KIDS [26], and a German trial [25] described in the previous sections. These studies were at low risk of bias [26], had some concerns [25] due to randomization, or were at high risk of bias [23] due to reporting (Supplemental Table 7).

**TABLE 5:** Relationships between seafood consumption during childhood and adolescence and movement/physical development, language/communication development, and depression/anxiety outcomes.

Study name	Outcome assessment tool	Results	
Movement/physical development (*n* = 3 parallel-arm RCTs), organized from youngest to oldest age at outcome assessment			
Polyunsaturated fatty acids in child nutrition (PINGU) [23]	Bayley Scales of Infant Development II	Mean (SD) at 10 mo: Psychomotor development Rapeseed oil group (*n* = 40): 100.4 (7.9) Salmon group (*n* = 39): 99.8 (9.2) Corn oil control group (*n* = 45): 100.2 (9.3) Group difference: *P* = 0.83	
Fish Intervention Studies–KIDS (FINS-KIDS) [26]	9-Hole Peg Test	Preintervention to postintervention change at 4–6 y, mean (95% CI): Dominant hand dexterity: Fish (herring and mackerel) group (*n* = 105): −2.7 (−3.6, −1.8) Meat (chicken, lamb, and beef) group (*n* = 113): −1.8 (−2.7, −1.0) Group difference: *P* = 0.19 (Per-protocol analysis also showed NS) Nondominant hand dexterity: Fish^1^ group (*n* = 105): −4.2 (−5.3, −3.2) Meat^2^ group (*n* = 113): −2.7 (−3.8, −1.7) Group difference: *P* = 0.0470 (Per-protocol analysis found a significantly larger decrease in time in fish group than that in meat group, *P* = 0.0110)	
Unnamed randomized controlled trial [25]	9-Hole Peg Test	Preintervention to postintervention change at 4–6 y, mean (95% CI): Dominant hand dexterity: Salmon group (*n* = 91): −2.0 (−2.9, −1.1) Meat (self-selected beef, turkey, or ham) group (*n* = 90): −3.0 (−3.8, −2.1) Group difference: *P* = 0.149 Nondominant hand dexterity: Salmon group (*n* = 91): −3.6 (−4.8, −2.4) Meat^3^ group (*n* = 90): −3.6 (−4.8, −2.4) Group difference: *P* = 0.976	
Language/communication development (*n* = 1 prospective cohort study)			
Odense Child Cohort [21]	MacArthur Bates Communicative Development Inventories	Fish^4^ intake at 18 mo, %, *n* = 999 MB-CDI ≤15th percentile Language complexity Never/hardly ever: 31 Weekly: 39 Daily: 30 Vocabulary Never/hardly ever: 34 Weekly: 41 Daily: 25	Fish^4^ intake at 18 mo, %, *n* = 999 MB-CDI >15th percentile Language complexity Never/hardly ever: 20 Weekly: 48 Daily: 31 Vocabulary Never/hardly ever: 22 Weekly: 46 Daily: 32
Depression/anxiety (*n* = 2 prospective cohort studies), organized from youngest to oldest age at outcome assessment			
Children’s Lifestyle and School Performance Study (CLASS) [34]	Internalizing disorder, including depression and anxiety	Association between number of daily servings of fish^4^ at age 10–11 y and number of health care provider contacts for internalizing disorder with follow-up through 13–14 y (*n* = 3757), IRR (95% CI): First tertile: REF Second tertile: 0.88 (0.56, 1.39) Third tertile: 0.59 (0.41, 0.87)	
ROOTS Study [35]	Moods and Feelings Questionnaire	Association between servings of fish^4^ per day at 15 y and depressive symptoms at 17 y, β (95% CI): Full sample (*n* = 603): 2.34 (−1.15, 5.83) Males (*n* = 241): −0.09 (−4.44, 4.27) Females (*n* = 362): 4.20 (−1.32, 9.72)	

Abbreviations: IRR, incident rate ratio; NS, nonsignificance; REF, referent.

1 Fish group included herring and mackerel.

2 Meat group included chicken, lamb, and beef.

3 Meat group included self-selected beef, turkey, or ham.

4 Authors used the term fish intake with no further description.

For 10-mo-old infants in the PINGU study [23], there were no statistical differences in psychomotor development scores among the salmon, rapeseed oil, or corn oil control groups and effect sizes between groups were <0.2 points. For children aged 4–6 y in FINS-KIDS [26], the children in the fish group had greater improvements in fine motor skills measured by dexterity on the 9-hole peg test.Differences between groups were <2 s andonly the nondominant hand was statistically significant. Differences between groups in the German trial [25] were negligible and not statistically significant. Overall, the evidence was limited in the number of studies, the age of participants, and outcome assessments and, thus, did not support a conclusion about relationships between seafood intake and movement/physical development for children and adolescents.

### Language/communication development

There was 1 PCS, the Odense Child Cohort [21], which assessed relationships between fish intake and language/communication development in children aged 18 mo (Table 5). In this study, children who scored >15th percentile for language and communication on the MacArthur Bates Communicative Development Inventories at 21 and 30 mo had statistically higher fish intake at 18 mo than children who scored <15th percentile. This study was at high risk of bias due to confounding and exposure measurement (Supplemental Table 8). Overall, evidence from 1 study of a single population was not sufficient to support conclusions about relationships between seafood intake and language/communication development for children and adolescents.

### Depression/anxiety

There were 2 PCSs (Table 5) that assessed relationships between seafood intake and depression or anxiety outcomes, the Children’s Lifestyle and School Performance Study [34] conducted in Canada and the ROOTS Study [35] conducted in the United Kingdom. Both studies had some concerns for bias due to confounding, selection bias, postexposure intervention, and missing data (Supplemental Table 9).

For Children’s Lifestyle and School Performance Study [34], higher self-reported fish intake for children aged 10–11 y was associated with ∼40% lower risk of being diagnosed with internalizing disorders, such as depression and anxiety, about 3 y later. Conversely, results from the ROOTS study [35] suggested a potential adverse association between fish intake at 15 y old and self-reported depressive symptoms from the Moods and Feelings Questionnaire at 17 y old. Neither of these results were statistically significant. Overall, the evidence was limited in the number of studies and assessed outcomes and results were inconsistent. Therefore, the evidence did not support a conclusion about seafood intake and depression or anxiety outcome for children and adolescents.

### ADHD or autism spectrum disorder

No evidence was identified for ADHD or autism spectrum disorder.

## Discussion

This was an updated search and synthesis based on the 2020 DGAC systematic review examining the relationship between seafood intake and neurocognitive development outcomes in children and adolescents [12]. The DGAC reported that there was insufficient evidence to determine a relationship but that there was no indication of adverse relationships of higher seafood intake and these outcomes in children and adolescents. With the addition of 5 new studies—2 RCTs [23,24] and 3 PCSs [[20], [21], [22]]—our results suggested that seafood consumption, mainly as fatty fish, may result in favorable cognitive development in children and adolescents aged 0–18 y. These conclusions were based on short-term RCTs that were at low risk or had some concerns of bias that aligned with results from longer term PCSs. The studies were conducted largely in Northern European populations and seafood consumption ranged from 5 to 11 oz/wk. The narrow scope of the study populations and seafood types contributed to the low certainty of evidence. More research is needed to assess whether seafood as a broader food group, particularly for types that are lower in essential ω-3 fatty acids, would improve other neurocognitive development outcomes, as well as in the longer term and in more diverse populations.

Previous research suggested that higher seafood intake is related to improved neurocognitive outcomes in children and adolescents [41], likely because of its unique nutrient composition. Fatty fish is the primary food source of essential ω-3 fatty acids, including DHA and EPA. Intake of ω-3 fatty acids, particularly ≥450 mg/d of supplementary DHA plus EPA, can improve cognition for typically developing children and adolescents [42]. Previous research also suggested beneficial effects of ω-3 fatty acid supplementation as an adjunct treatment for behavioral disorders in children [43,44]. In addition to higher ω-3 fatty acid intake, increasing fatty fish intake for children can also lead to higher intakes of protein, vitamin B-12, iron, and other nutrients that have previously been reported to have beneficial effects on cognitive development and behavior [45,46]. A complementary systematic review found an association between total seafood intake during pregnancy and improved neurocognitive development in the child [47]. In alignment with this body of literature, our results suggested that that higher seafood intake, consumed mostly as fatty fish, was related to improved cognitive development outcomes. Evidence was limited for other outcomes and unclear for behavior potentially due to small sample sizes and a lack of studies that were long enough to observe meaningful changes in behavior. However, our results add to the totality of research that suggest seafood intake during both the prenatal and postnatal stages may improve neurocognitive development of children and adolescents with little to no adverse outcomes.

A strength of the evidence in our systematic review was the availability of data from RCTs in which study foods were provided to participants in prepared meals either at home or at school. However, generalizability of this evidence was limited because all RCTs were conducted in Northern Europe, with little reported data on participant characteristics and a focus on fatty fish rather than other seafood types. The comparison dietary interventions were also similar, mostly meat and poultry, which can affect conclusions depending on what the underlying biological mechanisms are for differential effects [48,49]. The duration of the included RCTs was short (<16 wk) which may in part explain statistically null results and small effect sizes because the studies were likely not long enough to result in detectable changes over time within or between groups. There is a need for more longitudinal studies with repeated measures over time in the same children using validated dietary assessment tools and rigorous controls for confounding to better understand the trajectory of how seafood intake may be associated with development outcomes across childhood and adolescence and into adulthood.

The 2020–2025 Dietary Guidelines for Americans recommends that children and adolescents consume 8–10 oz/wk of low mercury seafood, depending on age and energy requirements [1]. The results of our systematic review align with these recommendations but with important nuance. The quantity of seafood consumed in the included articles of our systematic review (∼5–11 oz/wk) overlapped with currently recommended amounts for children and adolescents (8–10 oz/wk) [1], and the types of seafood assessed (eg, salmon, herring, and mackerel) tended to be low in mercury [1]. However, all RCTs and most PCSs in our systematic review assessed benefits of fatty fish consumption only, with little to no assessment of other seafood types. Therefore, there is a paucity of data about whether seafood in general would offer similar cognitive benefits to fatty fish. This nuance is important because fatty fish is higher in ω-3 fatty acids than other seafood types commonly consumed in the United States. There may also be differences in cooking or preparation methods, such as breading and frying of white fish (eg, fish sticks and fish fry) compared with baked or raw fatty fish (eg, salmon or tuna), which would affect intakes of energy, sodium, saturated fat, and potentially carcinogenic compounds formed from high heat cooking [50]. Future seafood recommendations for children and adolescents should specifically reflect the type of seafood and preparation methods used in the studies upon which the recommendation is based.

Mean seafood intake for all age groups in the United States, including children and adolescents, is well below current recommendations [1]. Less than 6% of those aged >1 y old in the United States consume seafood, inclusive of fish and shellfish, at least twice per week [13,51]. Almost half of children and adolescents aged 1–19 y consume seafood less than once per month [13]. Our results suggested that increasing seafood intake, particularly fatty fish, to amounts that are closer to current recommendations have potential to improve child cognition. However, there are several barriers to increasing seafood intake including taste preferences, familiarity, cooking skills, affordability, access, and concerns of contaminant exposure [[52], [53], [54]]. Further, seafood intake is higher among foreign-born than that in United States–born people [55] and intake varies by race, ethnicity, education level, geographic location, and cultural preferences [13,56]. Strategies to increase seafood and fatty fish intake for children and adolescents in the United States would require a multipronged approach, such as offering more seafood containing meals at schools and educating caregivers on benefits of consuming more seafood at home for both them and their children.

This systematic review has several strengths. It is rated as a high-quality systematic review according to AMSTAR 2 [15], followed PRISMA and DGAC methodology [12], the analytical framework was informed by a NASEM expert committee, and was conducted by an independent third-party research team to reduce bias. Our narrative synthesis considered the direction, magnitude, and statistical significance of results. This multipronged approach that considers information beyond statistical significance is important when meta-analyses cannot be performed [57]. This is because the absence of a statistically significant effect can be the result of the analysis being underpowered due to a small sample size, in which case, is not evidence that there is no true effect [58,59]. Meta-analyses are often used to account for this by pooling data to increase sample size and statistical power. We planned to conduct meta-analyses as indicated in our preregistered protocol but decided not to due to high heterogeneity in the methods used to assess outcomes and how the data were reported in the primary articles. Therefore, our conclusions are founded on narrative syntheses in which there is a level of subjectivity and expertise required. To alleviate these concerns, 2 analysts conducted the narrative synthesis independently, and conclusions were discussed until consensus was reached. Often in nutrition research, it is challenging to define a meaningful difference for a given outcome, particularly when there are multiple outcomes assessments per study and different utilization of tools and scoring systems across studies, as we faced in this systematic review. For example, a difference in 3 points on an intelligence scale may seem smalls, but may not be trivial when considering the short duration of the study and the potential for that effect to persist or compound longer term. An additional limitation of our approach was the inclusion of only peer-reviewed articles published in English as this increases risk for publication bias.

Seafood consumption at current recommended amounts consumed mainly as fatty fish may result in improved cognitive development outcomes in children and adolescents aged 0–18 y. It remains unclear whether seafood not rich in ω-3 fatty acids would elicit similar benefits. Public health efforts to increase seafood consumption in the United States population could help realize these potential benefits for children and adolescents.

## Author contributions

The authors’ responsibilities were as follows – MKS, AJM: designed the research; MKS, LEO, SS, AB: conducted the research; MKS, LEO, SS, AB: prepared the data; LEO: synthesized the data; LEO: wrote the paper with editorial assistance from MKS, AJM, SS, and AB; MKS, AJM; primary responsibility for the final content; and all authors: read and approved the final manuscript.

## Data availability

Data described in the manuscript, code book, and analytic code will be made publicly and freely available without restriction in Supplemental Data Appendix.

## Funding

This work was supported by a contract with the National Academies of Sciences, Engineering, and Medicine who contributed to the search and protocol development.

## Conflict of interest

AJM reports financial support was provided by National Academies of Sciences, Engineering, and Medicine. LEO reports funding grants from Beef Checkoff and, as a previous employee of the USDA and the NIH, has previous and ongoing projects funded by the Beef Checkoff, National Cancer Institute, and National Institute for Food and Agriculture for research unrelated to this work. AJM and MS were consultants for the National Academies of Sciences, Engineering, and Medicine committee on the Role of Seafood in Child Growth and Development. All other authors report no conflicts of interest.
