# Supplementary material for: Seafood and Neurocognitive Development in Children: A Systematic Review
Source: Adv Nutr. 2025 Feb 14;16(4):100391. doi: 10.1016/j.advnut.2025.100391 (PMC11930684; doi:10.1016/j.advnut.2025.100391)
Supplement: multimedia component 1 [file mmc1.docx]

Table of Contents

[**Supplementary Figure 1:** Analytical PICO framework for a systematic review about relationships between seafood consumption during childhood and adolescence and neurocognitive development 2](#_Toc191748766)

[**Supplementary Figure 2:** PRISMA flow diagram for a systematic review about relationships between seafood consumption during childhood and adolescence and neurocognitive development 3](#_Toc191748767)

[**Supplementary Table 1**: Organization of neurocognitive development outcomes 4](#_Toc191748768)

[**Supplementary Table 2**: Search strategy for a systematic review about relationships between seafood consumption during childhood and adolescence and neurocognitive development 6](#_Toc191748769)

[**Supplementary Table 3**: Inclusion and exclusion criteria for a systematic review about relationships between seafood consumption during childhood and adolescence and neurocognitive development 16](#_Toc191748770)

[**Supplementary Table 4**: Full text articles that were reviewed and excluded for a systematic review about relationships between seafood consumption during childhood and adolescence and neurocognitive development 19](#_Toc191748771)

[**Supplementary Table 5**: Risk of bias for studies that reported at least one result related to seafood consumption during childhood and adolescence and cognitive development 29](#_Toc191748772)

[**Supplementary Table 6**: Risk of bias for studies that reported at least one result related to seafood consumption during childhood and adolescence and behavior, inclusive of social-emotional and behavioral development 31](#_Toc191748773)

[**Supplementary Table 7**: Risk of bias for studies that reported at least one result related to seafood consumption during childhood and adolescence and movement/physical development 33](#_Toc191748774)

[**Supplementary Table 8**: Risk of bias for studies that reported at least one result related to seafood consumption during childhood and adolescence and language/communication development 34](#_Toc191748775)

[**Supplementary Table 9**: Risk of bias for studies that reported at least one result related to seafood consumption during childhood and adolescence and depression or anxiety 35](#_Toc191748776)

[**Supplementary Appendix 1:** PRISMA checklist 36](#_Toc191748777)

[**Supplementary Appendix 2:** AMSTAR 2 checklist assessing methodological quality of the systematic review 42](#_Toc191748778)

[Supplementary references 48](#_Toc191748779)

### **Supplementary Figure 1:** Analytical PICO framework for a systematic review about relationships between seafood consumption during childhood and adolescence and neurocognitive development

**
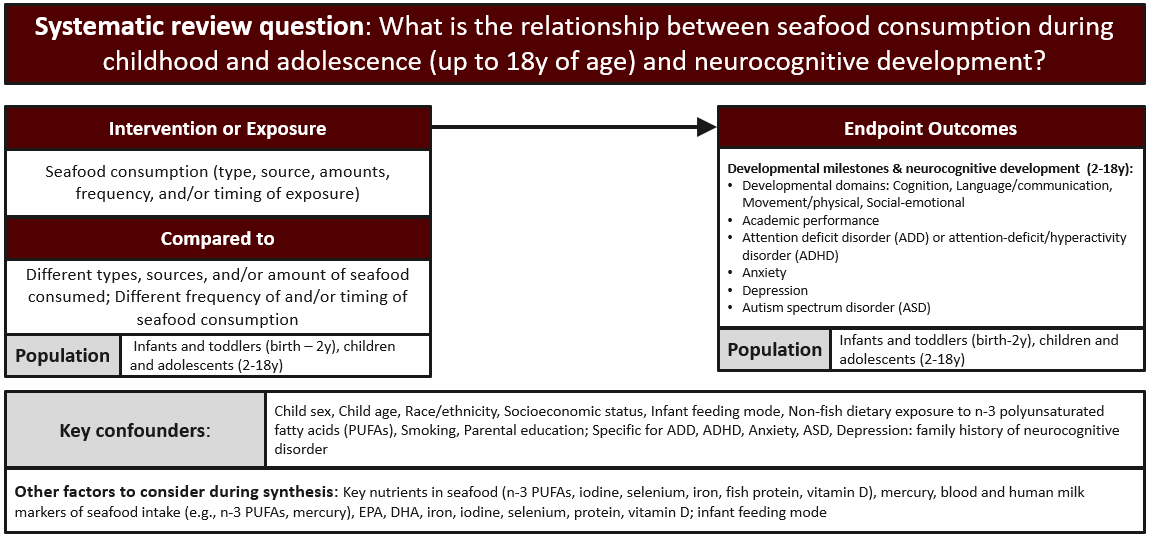
**

See Figure 1 of main text for organization of outcomes for data synthesis as well as Supplementary Table 1 for rationale. A diagnosis of ADD is no longer part of the most recent Diagnostic and Statistical Manual of Mental Disorders (DSM-5-TR), so the term ADHD is used to describe results. However, the term ADD was used in the database search for historical purposes. The term behavior was used instead of social-emotional in data synthesis and conclusions to be more encompassing of behaviors beyond social-emotional development that were captured in the assessment tools, such as the Strengths and Difficulties Questionnaire.

### **Supplementary Figure 2:** PRISMA flow diagram for a systematic review about relationships between seafood consumption during childhood and adolescence and neurocognitive development

Synthesize

Screen

Search

1391 records identified
through database searching

1245 records after duplicates removed; titles screened

146 duplicates removed

464 abstracts screened

360 excluded abstracts

104 full texts screened

87 excluded full texts

(see Supplementary Table 4 for reasons)

17 articles included

12 excluded full texts that were relevant for pregnancy/lactation review only

**5 articles included from new search**

**13 articles included from existing USDA review**

**18 articles included in this review**

781 excluded titles

This search was conducted to address two research questions: one related to seafood intake during pregnancy and lactation and neurocognitive development outcomes in the child, and one related to seafood intake during childhood and adolescence and neurocognitive development outcomes in the child. Additionally, relevant articles from a systematic review previously conducted by the USDA, as described in the methods of the main text, were included in the synthesis.

### **Supplementary Table 1**: Organization of neurocognitive development outcomes

| **Outcome categories from DGAC report*** | **Outcome categories in our review** | **Differences between DGAC and our review with references for outcome assessments** |
| --- | --- | --- |
| Cognitive development, including attention-related cognitive outcomes | Cognitive development, including attention-related cognitive outcomes | No change from DGAC report.  (Lesser and Pope, 2007) |
| Language and communication development | Language and communication development | No change from DGAC report.  (Lesser and Pope, 2007) |
| Movement and physical development | Movement/physical development | No change from DGAC report.  (Lesser and Pope, 2007) |
| Social-emotional and behavioral development | Behavior, inclusive of social-emotional and behavioral development | The umbrella term behavior was used in data synthesis and conclusions to be more encompassing of behaviors beyond social-emotional development that were captured in the assessment tools, such as in the Strengths and Difficulties Questionnaire.  (Lesser and Pope, 2007; Achenbach, 1999; Achenbach, 2001, Zubler et al, 2022) |
| Autism spectrum disorder-like traits or behaviors or autism spectrum disorder diagnosis | Autism spectrum disorder | No change from DGAC report.  (American Psychiatric Association, 2013) |
| Attention deficit disorder or attention-deficit/hyperactivity disorder-like traits or behaviors | Attention-Deficit/Hyperactivity Disorder (ADHD) | Traits, behaviors, and diagnosis were included in one category to align with the organization for Autism spectrum disorder. The term Attention deficit disorder (ADD) was removed from the category title because it is no longer used in the Diagnostic and Statistical Manual of Mental Disorders 5.  (American Psychiatric Association, 2013) |
| Attention deficit disorder or attention-deficit/hyperactivity disorder | Attention-Deficit/Hyperactivity Disorder (ADHD) | Traits, behaviors, and diagnosis were included in one category to align with the organization for Autism spectrum disorder used in the DGAC report (shown in row above). The term Attention deficit disorder (ADD) was removed from the category title because it is no longer used.  (American Psychiatric Association, 2013) |
| Academic performance | Cognitive development | Rather than being a separate outcome category, academic performance was reorganized to be included with cognitive development because academic performance is a measurable outcome of cognitive ability.  (Kuncel, 2004 and Vock, 2011) |
| Anxiety | Depression/anxiety | Anxiety and depression were combined into one category to better capture mental health outcomes more generally.  (WHO, 2024 and CDC, 2024) |
| Depression | Depression/anxiety | Anxiety and depression were combined into one category to better capture mental health outcomes more generally.  (WHO, 2024 and CDC, 2024) |

*Dietary Guidelines Advisory Committee report: Snetselaar L, Bailey R, Sabaté J, Van Horn L, Schneeman B, Spahn J, Kim JH, Bahnfleth C, Butera G, Terry N, Obbagy J. Seafood Consumption during Childhood and Adolescence and Neurocognitive Development: A Systematic Review. July 2020. U.S. Department of Agriculture, Food and Nutrition Service, Center for Nutrition Policy and Promotion, Nutrition Evidence Systematic Review. Available at: <https://doi.org/10.52570/NESR.DGAC2020.SR0503>

References used to inform outcome categorization:

Achenbach TM, Rescorla L. 2001. Manual for the ASEBA school-age forms & profiles: An integrated system of multi-informant assessment: Aseba Burlington, VT.

Achenbach, TM. (1999). The Child Behavior Checklist and related instruments. In M. E. Maruish (Ed.), The use of psychological testing for treatment planning and outcomes assessment (2nd ed., pp. 429–466). Lawrence Erlbaum Associates Publishers.

American Psychiatric Association. (2013). Diagnostic and statistical manual of mental disorders (5th ed.). <https://doi.org/10.1176/appi.books.9780890425596>

Centers for Disease Control and Prevention, About Mental Health. 2024. Available at: https://www.cdc.gov/mental-health/about/index.html

Kuncel NR, Hezlett SA, Ones DS. Academic performance, career potential, creativity, and job performance: can one construct predict them all? J Pers Soc Psychol. 2004 Jan;86(1):148-61. doi: 10.1037/0022-3514.86.1.148.

Lesser JG, Pope DS. Early Childhood the toddler and Early School Years. Human Behavior and the Social Environment: Theory and Practice: Pearson Allyn & Bacon; 2007. p. 264-70.

Vock M, Preckel F, Holling H. Mental abilities and school achievement: A test of a mediation hypothesis. Intelligence. 2011 Oct; 39(5): 357-369.

World Health Organization, Mental Health. 2024. Available at: <https://www.who.int/news-room/fact-sheets/detail/mental-health-strengthening-our-response>

Zubler JM, Wiggins LD, Macias MM, et al. Evidence-Informed Milestones for Developmental Surveillance Tools. Pediatrics March 2022; 149 (3): e2021052138. 10.1542/peds.2021-052138

### **Supplementary Table 2**: Search strategy for a systematic review about relationships between seafood consumption during childhood and adolescence and neurocognitive development

Database 1

Database: Pubmed

Search completed September 6, 2024

Limits: English

Search terms:

| #1 - "Brachyura"[Mesh] OR "Perciformes"[Mesh] OR "Decapodiformes"[Mesh] OR "Flounder"[Mesh] OR "Fish Flour"[Mesh] OR "Mya"[Mesh] OR "Bass"[Mesh] OR "Gadiformes"[Mesh] OR "Fish Proteins"[Mesh] OR "Fish Products"[Mesh] OR "Shellfish Proteins"[Mesh] OR "Salmo salar"[Mesh] OR "Tilapia"[Mesh] OR "Tuna"[Mesh] OR "Shellfish"[Mesh] OR "Methylmercury Compounds"[Mesh] OR "Cetacea"[Mesh] OR "Gadus morhua"[Mesh] OR "Bivalvia"[Mesh] OR "Seafood"[Mesh] OR "Catfishes"[Mesh] OR "Pectinidae"[Mesh] OR "Sharks"[Mesh] OR "Salmonidae"[Mesh] OR "Perches"[Mesh] OR "Ostreidae"[Mesh] OR "Astacoidea"[Mesh] OR "Fishes"[Mesh] OR "Fish Proteins, Dietary"[Mesh] OR "Mercury Poisoning"[Mesh] or seafood[tiab] or sea foods[tiab] or sea food[tiab] or sea-food[tiab] or sea-foods[tiab] or fish consumption[tiab] or fishes[tiab] or fish protein[tiab] or fish proteins[tiab] or fish product[tiab] or fish products[tiab] or fish meal[tiab] or fish flour[tiab] or fatty fish[tiab] or shellfish[tiab] or shellfish proteins[tiab] or mercurialism[tiab] or mercury poisoning[tiab] or methylmercury[tiab] or sharks[tiab] or swordfish[tiab] or tuna[tiab] or salmonine[tiab] or salmon[tiab] or sardine*[tiab] or sardines[tiab] or gadiformes[tiab] or pollock[tiab] or flounder[tiab] or cod[tiab] or tilapia[tiab] or shrimp[tiab] or oyster[tiab] or oysters[tiab] or clams[tiab] or scallops[tiab] or crab[tiab] or crabs[tiab] or perciformes[tiab] or mackerel[tiab] or catfishes[tiab] or trout[tiab] or lobster[tiab] or decapodiformes[tiab] or squid[tiab] or halibut[tiab] or mahi mahi[tiab] or crayfish[tiab] or crawfish[tiab] or anchovy[tiab] or herring[tiab] or rockfish[tiab] or marine product[tiab] or marine products[tiab] or marlin[tiab] or orange roughy[tiab] or tile fish[tiab] or whales[tiab] or perch[tiab] or walleye fish[tiab] or lake trout[tiab] or salmonid[tiab] or catfish[tiab] or sushi[tiab] or cerviche[tiab] or sashimi[tiab] or gravlax[tiab] or tuna tartare[tiab] or seafood crudo[tiab] or fluke crudo[tiab] or fluke[tiab] or carpaccio[tiab] or e-la-ota[tiab] or poke[tiab] or hinava[tiab] or gohu ikan[tiab] or esqueixada[tiab] or kelaguen[tiab] or namero[tiab] or kilawin[tiab] or stroganina[tiab] or yusheng[tiab] or koi pla[tiab] or kokoda[tiab] or kuai[tiab] or lakerda[tiab] or larb pla[tiab] or ota ika[tiab] or tiradito[tiab] or xato[tiab] or umai[tiab] or salmo salar[tiab] or atlantic salmon[tiab] or salmonids[tiab] |
| --- |
| #2 - "Phytic Acid"[Mesh] OR "Food Analysis"[Mesh] OR "Diet, Mediterranean"[Mesh] OR "Taurine"[Mesh] OR "Folic Acid"[Mesh] OR "Eicosapentaenoic Acid"[Mesh] OR "Zinc"[Mesh] OR "Vitamin D"[Mesh] OR "Selenium"[Mesh] OR "Nutritional Status"[Mesh] OR "Iron"[Mesh] OR "Iodine"[Mesh] OR "Food"[Mesh] OR "Drinking"[Mesh] OR "Diet"[Mesh] OR "Choline"[Mesh] OR "Calcium"[Mesh] OR "Diet, Food, and Nutrition"[Mesh] OR "Fatty Acids, Omega-3"[Mesh] OR "Docosahexaenoic Acids"[Mesh] OR "Dietary Supplements"[Mesh] OR "Meals"[Mesh] OR "Fatty Acids"[Mesh] OR "Fish Proteins"[Mesh] OR "Proteins"[Mesh] OR "Nutritive Value"[Mesh] OR "Recommended Dietary Allowances"[Mesh] OR "Polyphenols"[Mesh] OR "Diet, Healthy"[Mesh] OR "Diet Records"[Mesh] OR "Infant Nutritional Physiological Phenomena"[Mesh] OR "Eating"[Mesh] or Magnesium[mh] or diet[tiab] or dietary supplement[tiab] or dietary supplements[tiab] or food[tiab] or nutritional status[tiab] or iron[tiab] or folic acid[tiab] or calcium[tiab] or Zinc[tiab] or phytate[tiab] or phytic acid[tiab] or polyphenol[tiab] or polyphenols[tiab] or fish species[tiab] or fish tissue[tiab] or fish intake[tiab] or fish-rich diet[tiab] or fatty acids[tiab] or fatty acid[tiab] or fish protein[tiab] or mediterranean diet[tiab] or omega-3 fatty acids[tiab] or omega 3 fatty acids[tiab] or omega-3 fatty acid[tiab] or omega 3 fatty acid[tiab] or fish consumption[tiab] or eicosapentaenoic acid[tiab] or docosahexaenoic acid[tiab] or docosapentaenoic acid[tiab] or meals[tiab] or food intake[tiab] or dietary intake[tiab] or magnesium[tiab] |
| #3 - "Hexachlorobenzene"[Mesh] OR "Mercury"[Mesh] OR "Heptachlor"[Mesh] OR "Endrin"[Mesh] OR "Dieldrin"[Mesh] OR "DDT"[Mesh] OR "Arsenic"[Mesh] OR "Aldrin"[Mesh] OR "Mercury Compounds"[Mesh] OR "Mercury Poisoning, Nervous System"[Mesh] OR "Cyclohexane Monoterpenes"[Mesh] OR "Dioxins and Dioxin-like Compounds"[Mesh] OR "Fluorocarbons"[Mesh] OR "Nanofibers"[Mesh] OR "Polychlorinated Biphenyls"[Mesh] OR "Chlordan"[Mesh] OR "Arsenic Poisoning"[Mesh] OR "Microplastics"[Mesh] OR "Dioxins"[Mesh] OR "Methylmercury Compounds"[Mesh] or Cadmium[mh] or lead[mh] or chlorpyrifos[mh] or Harmful Algal Bloom[mh] or Cyanobacteria[mh] or Ciguatera Poisoning[mh] or Hepatitis A virus[mh] or Salmonella[mh] or Escherichia coli[mh] or polyfluoroalkyl substance*[tiab] or pfas[tiab] or methylmercury compounds[tiab] or methylmercury-toxicity[tiab] or organomercury compound[tiab] or organic mercury compound[tiab] or mercury exposure*[tiab] or organomercury[tiab] or methylmercury-induced cytotoxicity[tiab] or organomercurials[tiab] or mercurial*[tiab] or polychlorinated biphenyl[tiab] or polychlorinated biphenyls[tiab] or polychlorobiphenyl compounds[tiab] or methylmercury[tiab] or methyl mercury[tiab] or methyl-mercury[tiab] or mehg[tiab] or monomethylmercury[tiab] or methylquecksilber[tiab] or dioxin[tiab] or dioxins[tiab] or inorganic mercury[tiab] or pfos[tiab] or pfoa[tiab] or campheclor[tiab] or toxaphene[tiab] or dichlorodiphenyltrichlorethane[tiab] or chlordane[tiab] or chlordan[tiab] or dieldrin[tiab] or hexachlorobenzene[tiab] or cyclohexane[tiab] or brominated flame retardant*[tiab] or diphenyl ether[tiab] or diphenyl ethers[tiab] or polybrominated diphenyl ether[tiab] or polybrominated diphenyl ethers[tiab] or aquatic toxicology[tiab] or aquatic toxicity[tiab] or aldrin[tiab] or arsenic[tiab] or dibenzofuran*[tiab] or endrin[tiab] or fluorocarbon*[tiab] or furan*[tiab] or heptachlor[tiab] or microfiber*[tiab] or microplastic*[tiab] or nanofiber*[tiab] or nanoplastic*[tiab] or organic-fluorine*[tiab] or polyfluoroalkyl substance*[tiab] or total organic fluorine[tiab] or cadmium[tiab] or Chlorpyrifos[tiab] or lorsban[tiab] or dursban[tiab] or Algal Bloom Harmful[tiab] or Algal Blooms Harmful[tiab] or Bloom Harmful Algal[tiab] or Blooms Harmful Algal[tiab] or Harmful Algal Blooms[tiab] or Red Tide[tiab] or Red Tides[tiab] or Tide Red[tiab] or Tides Red[tiab] or Cyanobacteria[tiab] or Bacteria Blue Green[tiab] or Bacteria Blue-Green[tiab] or Blue Green Bacteria[tiab] or Algae Blue-Green[tiab] or Algae Blue Green[tiab] or Blue Green Algae[tiab] or Blue-Green Algae[tiab] or Cyanophyceae[tiab] or Blue-Green Bacteria[tiab] or Ciguatera[tiab] or Ciguatera Poisonings[tiab] or Poisoning Ciguatera[tiab] or Poisonings Ciguatera[tiab] or Ciguatera Fish Poisoning[tiab] or Ciguatera Fish Poisonings[tiab] or Poisoning Ciguatera Fish[tiab] or Poisonings Ciguatera Fish[tiab] or Ciguater[tiab] or Scombroid[tiab] or Domoic acid[tiab] or Hepatitis A viruses[tiab] or hepatitis A virus[tiab] or Salmonella[tiab] |
| #4 - "Blindness"[Mesh] OR "Neoplasms"[Mesh] OR "Mental Disorders"[Mesh] OR "Thyroid Diseases"[Mesh] OR "Hypersensitivity"[Mesh] OR "Mortality"[Mesh] OR "Psychomotor Performance"[Mesh] OR "Muscle Weakness"[Mesh] OR "Congenital, Hereditary, and Neonatal Diseases and Abnormalities"[Mesh] OR "Alzheimer Disease"[Mesh] OR "Language Development Disorders"[Mesh] OR "Ataxia"[Mesh] OR "Developmental Disabilities"[Mesh] OR "Attention Deficit Disorder with Hyperactivity"[Mesh] OR "Cell Proliferation"[Mesh] OR "Metacognition"[Mesh] OR "Motor Skills"[Mesh] OR "Cognition"[Mesh] OR "Hypotension"[Mesh] OR "Hypertension"[Mesh] OR "Growth Disorders"[Mesh] OR "Executive Function"[Mesh] OR "Growth and Development"[Mesh] OR "Asperger Syndrome"[Mesh] OR "Risk Assessment"[Mesh] OR "Oxidative Stress"[Mesh] OR "Apoptosis"[Mesh] OR "Outcome Assessment, Health Care"[Mesh] OR "Autoimmunity"[Mesh] OR "Tremor"[Mesh] OR "Thyroxine"[Mesh] OR "Thyroid Gland"[Mesh] OR "Shyness"[Mesh] OR "Sexual Maturation"[Mesh] OR "Reading"[Mesh] OR "Puberty"[Mesh] OR "Problem Solving"[Mesh] OR "Paresthesia"[Mesh] OR "Morbidity"[Mesh] OR "Mental Health"[Mesh] OR "Memory"[Mesh] OR "Inflammation"[Mesh] OR "Infant, Low Birth Weight"[Mesh] OR "Immunity"[Mesh] OR "Growth"[Mesh] OR "Goiter"[Mesh] OR "Depression"[Mesh] OR "Dementia"[Mesh] OR "Coronary Artery Disease"[Mesh] OR "Chronic Disease"[Mesh] OR "Child Development"[Mesh] OR "Central Nervous System"[Mesh] OR "Cell Differentiation"[Mesh] OR "Brain"[Mesh] OR "Blood Pressure"[Mesh] OR "Attention"[Mesh] OR "Asthma"[Mesh] OR "Anxiety"[Mesh] OR "Allergy and Immunology"[Mesh] OR "Acrodynia"[Mesh] OR "Memory and Learning Tests"[Mesh] OR "Central Nervous System Sensitization"[Mesh] OR "Child Behavior Disorders"[Mesh] OR "Fractures, Bone"[Mesh] OR "Risk Factors"[Mesh] OR "Depressive Disorder"[Mesh] OR "Autistic Disorder"[Mesh] OR "Vision Disorders"[Mesh] OR "Learning Disabilities"[Mesh] OR "Diabetes Mellitus, Type 2"[Mesh] OR "Child Nutritional Physiological Phenomena"[Mesh] OR "Frailty"[Mesh] OR "Hearing Loss"[Mesh] OR "Biomarkers"[Mesh] OR "Sleep Wake Disorders"[Mesh] OR "Kidney Diseases"[Mesh] OR "Cardiovascular Diseases"[Mesh] OR "Autoimmune Diseases"[Mesh] OR "Accidental Falls"[Mesh] OR "Academic Success"[Mesh] OR "Immune System Phenomena"[Mesh] OR "Vision, Low"[Mesh] OR "Irritable Mood"[Mesh] OR "Congenital Abnormalities"[Mesh] OR "Motor Skills Disorders"[Mesh] OR "Attention Deficit and Disruptive Behavior Disorders"[Mesh] OR "Central Nervous System Infections"[Mesh] OR "Brain Diseases"[Mesh] OR "Growth Charts"[Mesh] or Failure to Thrive[mh] or malnutrition[mh] or protein deficiency[mh] or Dyslipidemias[mh] or Cholesterol[mh] or "Cholesterol, LDL"[Mesh] or Triglycerides[mh] or "Lipoproteins, HDL"[Mesh] or mental disorder[tiab] or mental disorders[tiab] or cognitive[tiab] or neurocognitive[tiab] or neurodevelop*[tiab] or neurological[tiab] or Alzheimer*[tiab] or senility[tiab] or senile[tiab] or presenile[tiab] or motor skills[tiab] or attention-deficit-disorder[tiab] or attention-deficit-disorders[tiab] or ADHD[tiab] or Autism-Spectrum-Disorder[tiab] or Asperger[tiab] or academic performance[tiab] or academic failure[tiab] or academic success[tiab] or mental process*[tiab] or congenital abnormality[tiab] or congenital abnormalities[tiab] or birth defects[tiab] or birth defect[tiab] or chronic condition*[tiab] or changes-in-vision[tiab] or vision problem[tiab] or changes-in-hearing[tiab] or deafness[tiab] or gastrointestinal[tiab] or respiratory[tiab] or obstruction-of-visual-field[tiab] or pink disease[tiab] or renal disease*[tiab] or tubular injury[tiab] or cancer[tiab] or learning disabilit*[tiab] or communication*[tiab] or social-interactive-skills[tiab] or functional disabilities[tiab] or functional disability[tiab] or sensorineural defect[tiab] or sensorineural defects[tiab] or vision loss[tiab] or hearing loss[tiab] or psychological disorder*[tiab] or behavioral disorder*[tiab] or Asperge*[tiab] or coronary disease*[tiab] or cardiac death[tiab] or Growth-and-development[tiab] or diabetes-mellitus-Type-2[tiab] or Metabolic outcome*[tiab] or accidental fall*[tiab] or thyroid*[tiab] or triiodothyronine[tiab] or mental-growth-retardation[tiab] or immune-system-phenomena[tiab] or hazardous substances[tiab] or hazardous substance[tiab] or risk factors[tiab] or risk factor[tiab] or risk-benefit[tiab] or thyroid dysfunction[tiab] or thyroid defect[tiab] or thyroid defects[tiab] or pre-term[tiab] or neurodevelopmental disorder*[tiab] or cardiovascular disease*[tiab] or toenail biomarker[tiab] or neurotoxicity syndrome*[tiab] or academic achievement*[tiab] or acrodynia[tiab] or allergy[tiab] or anxiety[tiab] or apoptosis[tiab] or areflexia[tiab] or asthma[tiab] or ataxia[tiab] or atopy[tiab] or attention[tiab] or autism[tiab] or autoimmune disease*[tiab] or autoimmunity[tiab] or blood pressure[tiab] or cell differentiation[tiab] or cell proliferation[tiab] or central nervous system[tiab] or child behavior disorder*[tiab] or child behaviour disorder*[tiab] or child development[tiab] or child nutritional physiological phenomena[tiab] or child nutritional physiological phenomena[tiab] or chronic disease*[tiab] or cognition[tiab] or dementia[tiab] or depression[tiab] or developmental delay*[tiab] or developmental disorder*[tiab] or environmental disease[tiab] or disorders-of-environmental-origin[tiab] or executive function[tiab] or fine motor[tiab] or bone-fracture*[tiab] or fractures-bone[tiab] or goiter[tiab] or gross motor[tiab] or health hazard[tiab] or hypersensitivity[tiab] or hypertension[tiab] or hypotension[tiab] or immune function[tiab] or immune system phenomena[tiab] or immunity[tiab] or inflammation[tiab] or irritability[tiab] or kidney disease[tiab] or language delay*[tiab] or language processing[tiab] or low birth weight[tiab] or memory[tiab] or mental growth retardation[tiab] or mental health[tiab] or metacognition[tiab] or morbidity[tiab] or mortality[tiab] or motor coordination[tiab] or muscle weakness[tiab] or neoplasms[tiab] or neuropathy[tiab] or outcome assessment[tiab] or oxidative stress[tiab] or paresthesia[tiab] or prematurity[tiab] or premature birth[tiab] or problem solving[tiab] or psychomotor performance[tiab] or puberty[tiab] or reading[tiab] or risk assessment*[tiab] or school readiness[tiab] or sensitization[tiab] or sexual maturation[tiab] or sexual maturity[tiab] or shyness[tiab] or sleep disorder*[tiab] or social interactive skill*[tiab] or tremor[tiab] or tubular dysfunction[tiab] or tubular injury[tiab] or verbal memory[tiab] or vision loss[tiab] or vision problem[tiab] or weakness[tiab] or Failure to thrive[tiab] or malnutrition[tiab] or protein deficiency[tiab] or Dyslipidemias[tiab] or cholesterol[tiab] or Low Density Lipoprotein Cholesterol[tiab] or beta-Lipoprotein Cholesterol[tiab] or Cholesterol beta-Lipoprotein[tiab] or beta Lipoprotein Cholesterol[tiab] or LDL Cholesterol[tiab] or Cholesteryl Linoleate LDL[tiab] or LDL Cholesteryl Linoleate[tiab] or triglycerides[tiab] or Triacylglycerols[tiab] or Triacylglycerol[tiab] or Triglyceride[tiab] or HDL Lipoproteins[tiab] or High-Density Lipoprotein[tiab] or Lipoprotein High-Density[tiab] or High-Density Lipoproteins[tiab] or High Density Lipoproteins[tiab] or Lipoproteins High-Density[tiab] or alpha-Lipoproteins[tiab] or alpha Lipoproteins[tiab] or Heavy Lipoproteins[tiab] or Lipoproteins Heavy[tiab] or High Density Lipoprotein[tiab] or Density Lipoprotein High[tiab] or Lipoprotein High Density[tiab] or alpha-Lipoprotein[tiab] or alpha Lipoprotein[tiab] or alpha-1 Lipoprotein[tiab] |
| #5 – (#1 AND #2 AND #3 AND #4) |
| #6 - #5 NOT ("Animals"[Mesh] NOT ("Animals"[Mesh] AND "Humans"[Mesh])) NOT (editorial[ptyp] OR comment[ptyp] OR news[ptyp] OR letter[ptyp] OR review[ptyp] OR systematic review[ptyp] OR systematic review[ti] OR meta-analysis[ptyp] OR meta-analysis[ti] OR meta-analyses[ti] OR retracted publication[ptyp] OR retraction of publication[ptyp] OR retraction of publication[tiab] OR retraction notice[ti]) |
| #7 - (catarrhini[mh:noexp] OR cercopithecidae[mh] OR gorilla gorilla[mh] OR haplorhini[mh:noexp] OR hominidae[mh:noexp] OR hylobatidae[mh] OR pan paniscus[mh] OR pan troglodytes[mh] OR platyrrhini[mh] OR pongo[mh] OR primates[mh:noexp] OR strepsirhini[mh] OR tarsii[mh] or Muridae[mh] or Murinae[mh] or Macaca[mh] or ape[tiab] apeas[tiab] or macaca[tiab] or macaque[tiab] or allenopithecus[tiab] OR allocebus[tiab] OR alouatta[tiab] OR alouattinae[tiab] OR angwantibo*[tiab] OR anthropoid[tiab] OR anthropoidea[tiab] OR anthropoids[tiab] OR aotes[tiab] OR aotidae[tiab] OR aotinae[tiab] OR aotus[tiab] OR ape[tiab] OR apes[tiab] OR arctocebus[tiab] OR ateles[tiab] OR atelidae[tiab] OR atelinae[tiab] OR avahi[tiab] OR aye-aye*[tiab] OR baboon[tiab] OR baboons[tiab] OR bonobo[tiab] OR bonobos[tiab] OR brachyteles[tiab] OR bushbabies[tiab] OR bushbaby[tiab] OR cacajao[tiab] OR callibella[tiab] OR callicebinae[tiab] OR callicebus[tiab] OR callimico[tiab] OR callithrichid*[tiab] OR callithrichinae[tiab] OR callithrix[tiab] OR callitrichid[tiab] OR callitrichidae[tiab] OR callitrichide[tiab] OR callitrichids[tiab] OR callitrichinae[tiab] OR capuchin[tiab] OR capuchins[tiab] OR carlito syrichta[tiab] OR catarhine*[tiab] OR catarhini[tiab] OR catarrhina[tiab] OR catarrhine*[tiab] OR catarrhini[tiab] OR cebid[tiab] OR cebidae[tiab] OR cebids[tiab] OR cebinae[tiab] OR ceboidea[tiab] OR cebuella[tiab] OR cebus[tiab] OR cephalopachus[tiab] OR cercocebus[tiab] OR cercopithecid*[tiab] OR cercopithecinae[tiab] OR cercopithecine*[tiab] OR cercopithecini[tiab] OR cercopithecoid[tiab] OR cercopithecoidea[tiab] OR cercopithecoids[tiab] OR cercopithecus[tiab] OR cheirogaleidae[tiab] OR cheirogaleus[tiab] OR cheracebus[tiab] OR chimp[tiab] OR chimpanzee[tiab] OR chimpanzees[tiab] OR chimps[tiab] OR chiromyiformes[tiab] OR chiropotes[tiab] OR chlorocebus[tiab] OR colobidae[tiab] OR colobinae[tiab] OR colobine*[tiab] OR colobini[tiab] OR colobus*[tiab] OR cynomolgus[tiab] OR daubentonia[tiab] OR daubentoniidae[tiab] OR douc[tiab] OR doucs[tiab] OR erythrocebus[tiab] OR eulemur[tiab] OR euoticus[tiab] OR euprimate*[tiab] OR galagid*[tiab] OR galago[tiab] OR galagoides[tiab] OR galagonidae[tiab] OR galagos[tiab] OR gelada[tiab] OR geladas[tiab] OR gibbon[tiab] OR gibbons[tiab] OR gorilla[tiab] OR gorillas[tiab] OR grivet[tiab] OR grivets[tiab] OR guenon*[tiab] OR guereza*[tiab] OR hapalemur[tiab] OR haplorhine*[tiab] OR haplorhini[tiab] OR haplorrhine*[tiab] OR haplorrhini[tiab] OR hominid*[tiab] OR hominin[tiab] OR homininae[tiab] OR hominine[tiab] OR hominines[tiab] OR hominini[tiab] OR hominins[tiab] OR hominoidea[tiab] OR hoolock[tiab] OR howler*[tiab] OR hylobates[tiab] OR hylobatidae[tiab] OR indri[tiab] OR indridae[tiab] OR indriid*[tiab] OR indris[tiab] OR kipunji*[tiab] OR lagothrix[tiab] OR langur[tiab] OR langurs[tiab] OR lemur[tiab] OR lemurid*[tiab] OR lemuriform[tiab] OR lemuriformes[tiab] OR lemuriforms[tiab] OR lemurinae[tiab] OR lemuroidea[tiab] OR lemurs[tiab] OR leontideus[tiab] OR leontocebus[tiab] OR leontopithecus[tiab] OR lepilemur[tiab] OR lepilemurid*[tiab] OR lesula*[tiab] OR lophocebus[tiab] OR loriform[tiab] OR loriformes[tiab] OR lorinae[tiab] OR loris[tiab] OR lorises[tiab] OR lorisid*[tiab] OR lorisiform*[tiab] OR lorisinae[tiab] OR lorisoid*[tiab] OR lutung[tiab] OR lutungs[tiab] OR macaca[tiab] OR macaque's[tiab] OR macaque[tiab] OR macaques[tiab] OR malbrouck*[tiab] OR mandrill[tiab] OR mandrills[tiab] OR mandrillus[tiab] OR mangabey*[tiab] OR marmoset[tiab] OR marmosets[tiab] OR mico argentatus[tiab] OR mico chrysoleucos[tiab] OR mico emiliae[tiab] OR mico humilis[tiab] OR mico marcai[tiab] OR mico melanurus[tiab] OR mico rondoni[tiab] OR microcebus[tiab] OR miopithecus[tiab] OR mirza coquereli[tiab] OR mirza zaza[tiab] OR monkey[tiab] OR monkeys[tiab] OR muriqui*[tiab] OR nasalis larvatus[tiab] OR nomascus[tiab] OR nycticebus[tiab] OR oedipomidas[tiab] OR orang utan*[tiab] OR orang-utan*[tiab] OR orangutan*[tiab] OR oreonax[tiab] OR otolemur[tiab] OR pan paniscus[tiab] OR pan troglodytes[tiab] OR panin[tiab] OR panina[tiab] OR panins[tiab] OR papio[tiab] OR papionini[tiab] OR paragalago[tiab] OR perodicticinae[tiab] OR perodicticus[tiab] OR phaner[tiab] OR piliocolobus[tiab] OR pithecia[tiab] OR pithecidae[tiab] OR pitheciid*[tiab] OR pitheciinae[tiab] OR pithecinae[tiab] OR platyrhine*[tiab] OR platyrhini[tiab] OR platyrrhina[tiab] OR platyrrhine*[tiab] OR platyrrhini[tiab] OR plecturocebus[tiab] OR pongid*[tiab] OR ponginae[tiab] OR pongo[tiab] OR potto[tiab] OR pottos[tiab] OR presbytini[tiab] OR presbytis[tiab] OR primate[tiab] OR primates[tiab] OR procolobus[tiab] OR prolemur[tiab] OR propithecus[tiab] OR prosimian*[tiab] OR prosimii[tiab] OR pseudopotto[tiab] OR pygathrix[tiab] OR rhinopithecus[tiab] OR rungwecebus[tiab] OR saguinus[tiab] OR saimiri[tiab] OR saimiriinae[tiab] OR sapajus[tiab] OR sciurocheirus[tiab] OR semnopithecus[tiab] OR siamang[tiab] OR siamangs[tiab] OR sifaka[tiab] OR sifakas[tiab] OR simians[tiab] OR simias[tiab] OR simiiform*[tiab] OR strepsir*[tiab] OR surili*[tiab] OR symphalangus[tiab] OR talapoin*[tiab] OR tamarin[tiab] OR tamarins[tiab] OR tamarinus[tiab] OR tarsier[tiab] OR tarsiers[tiab] OR tarsiid*[tiab] OR tarsiiform*[tiab] OR tarsius[tiab] OR theropithecus[tiab] OR trachypithecus[tiab] OR uacari*[tiab] OR uakari[tiab] OR uakaris[tiab] OR varecia[tiab] OR vervet*[tiab]) |
| #8 - #5 AND #7 |
| #9 - #8 NOT (editorial[ptyp] OR comment[ptyp] OR news[ptyp] OR letter[ptyp] OR review[ptyp] OR systematic review[ptyp] OR systematic review[ti] OR meta-analysis[ptyp] OR meta-analysis[ti] OR meta-analyses[ti] OR retracted publication[ptyp] OR retraction of publication[ptyp] OR retraction of publication[tiab] OR retraction notice[ti]) |
| Filters: Publication date from 2023/07/01; English |

Database 2

Database: Embase

Provider: Ovid

Search completed September 6, 2024

Limits: English

Search terms:

| 1 | exp sea food/ or exp fish/ or exp fish consumption/ or exp fish protein/ or exp fish product/ or exp fish flour/ or exp shellfish/ or exp shellfish protein/ or exp mercurialism/ or exp methylmercury/ or exp shark/ or exp swordfish/ or exp tuna/ or exp salmonine/ or exp sardine/ or exp gadiformes/ or exp flounder/ or exp atlantic cod/ or exp cod/ or exp tilapia/ or exp shrimp/ or exp oyster/ or exp mya/ or exp bivalve/ or exp clam/ or exp scallop/ or exp brachyura/ or exp crab/ or exp perciformes/ or exp mackerel/ or exp lobster/ or exp decapodiformes/ or exp squid/ or exp halibut/ or exp crayfish/ or exp anchovy/ or exp herring/ or exp rockfish/ or exp cetacea/ or exp perch/ or exp bass/ or exp lake trout/ or exp catfish/ or exp sushi/ or exp salmo salar/ or exp salmonid/ |
| --- | --- |
| 2 | (seafood or sea foods or sea food or sea-food or sea-foods or fish consumption or fishes or fish protein or fish proteins or fish product or fish products or fish meal or fish flour or fatty fish or shellfish or shellfish proteins or mercurialism or mercury poisoning or methylmercury or sharks or swordfish or tuna or salmonine or salmon or sardine* or sardines or gadiformes or pollock or flounder or cod or tilapia or shrimp or oyster or oysters or clams or scallops or crab or crabs or perciformes or mackerel or catfishes or trout or lobster or decapodiformes or squid or halibut or mahi mahi or crayfish or crawfish or anchovy or herring or rockfish or marine product or marine products or marlin or orange roughy or tile fish or whales or perch or walleye fish or lake trout or salmonid or catfish or sushi or cerviche or sashimi or gravlax or tuna tartare or seafood crudo or fluke crudo or fluke or carpaccio or e-la-ota or poke or hinava or gohu ikan or esqueixada or kelaguen or namero or kilawin or stroganina or yusheng or koi pla or kokoda or kuai or lakerda or larb pla or ota ika or tiradito or xato or umai or salmo salar or atlantic salmon or salmonids).ti,ab,kw. |
| 3 | or/1-2 |
| 4 | exp diet/ or exp dietary supplement/ or exp food/ or exp nutritional status/ or exp food analysis/ or exp nutritional value/ or exp iron/ or exp folic acid/ or exp calcium/ or exp zinc/ or exp phytate/ or exp phytic acid/ or exp polyphenol/ or exp omega 3 fatty acid/ or exp fatty acid/ or exp fish protein/ or exp mediterranean diet/ or exp fish consumption/ or exp eicosapentaenoic acid/ or exp docosapentaenoic acid/ or exp food intake/ or exp docosahexaenoic acid/ or exp meal/ or exp dietary intake/ or exp protein/ or exp vitamin D/ or exp choline/ or exp taurine/ or exp Iodine/ or exp selenium/ or exp drinking/ or exp eating/ or exp infant feeding/ or exp meat consumption/ or exp amino acid intake/ or exp dietary reference intake/ or exp fat intake/ or exp mineral intake/ or exp iodine intake/ or exp nutrient intake/ or exp protein intake/ or exp tolerable daily intake/ or exp vitamin intake/ or exp magnesium/ |
| 5 | (diet or dietary supplement or dietary supplements or food or nutritional status or iron or folic acid or calcium or Zinc or phytate or phytic acid or polyphenol or polyphenols or fish species or fish tissue or fish intake or fish-rich diet or fatty acids or fatty acid or fish protein or mediterranean diet or omega-3 fatty acids or omega 3 fatty acids or omega-3 fatty acid or omega 3 fatty acid or fish consumption or eicosapentaenoic acid or docosahexaenoic acid or docosapentaenoic acid or meals or food intake or dietary intake or magnesium).ti,ab,kw. |
| 6 | or/4-5 |
| 7 | exp organomercury compound/ or exp dioxins/ or exp polychlorinated biphenyl/ or exp methylmercury/ or exp monomethylmercury/ or exp dioxin/ or exp mercury/ or exp inorganic mercury/ or exp campheclor/ or exp ddt/ or exp chlordane/ or exp dieldrin/ or exp aldrin/ or exp endrin/ or exp heptachlor/ or exp hexachlorobenzene/ or exp cyclohexane/ or exp brominated flame retardant/ or exp diphenyl ether/ or exp polybrominated diphenyl ether/ or exp aquatic toxicology/ or exp aquatic toxicity/ or exp arsenic/ or exp dibenzofuran/ or exp fluorocarbon/ or exp microplastic/ or exp nanofiber/ or exp nanoplastic/ or exp cadmium/ or exp lead/ or exp chlorpyrifos/ or exp algal bloom/ or exp cyanobacterium/ or exp ciguatera/ or exp scombroid poisoning/ or exp domoic acid/ or exp Hepatitis A virus/ or exp Salmonella/ or exp Escherichia coli/ |
| 8 | (polyfluoroalkyl substance* or pfas or methylmercury compounds or methylmercury-toxicity or organomercury compound or organic mercury compound or mercury exposure* or organomercury or methylmercury-induced cytotoxicity or organomercurials or mercurial* or polychlorinated biphenyl or polychlorinated biphenyls or polychlorobiphenyl compounds or methylmercury or methyl mercury or methyl-mercury or mehg or monomethylmercury or methylquecksilber or dioxin or dioxins or inorganic mercury or pfos or pfoa or campheclor or toxaphene or dichlorodiphenyltrichlorethane or chlordane or chlordan or dieldrin or hexachlorobenzene or cyclohexane or brominated flame retardant* or diphenyl ether or diphenyl ethers or polybrominated diphenyl ether or polybrominated diphenyl ethers or aquatic toxicology or aquatic toxicity or aldrin or arsenic or dibenzofuran* or endrin or fluorocarbon* or furan* or heptachlor or microfiber* or microplastic* or nanofiber* or nanoplastic* or organic-fluorine* or polyfluoroalkyl substance* or total organic fluorine or cadmium or 208Pb or inorganic-lead or lead-208 or lead-ion or lead-ore or lead-radioisotope or lead-radioisotopes or plumbum or radioisotope-lead or chloropyrifos or chlorpyrifos-ethyl or chlorpyriphos or dursban or lorsban or o-3-5-6-trichloropyrid-2-yl-o-o-diethyl-phosphorothioate or o-o-diethyl-o-3-5-6-trichloropyrid-2-yl-phosphorothioate or o-o-diethyl-o-3-5-6-trichloro-2-pyridyl-phosphorothioate or o-o-diethyl-o-3-5-6-trichloro-2-pyridylphosphorothioate or phosphorothioic-acid-o-o-diethyl-o-3-5-6-trichloropyrid-2-yl-ester or harmful-algal-bloom or phytoplankton-bloom or blue-green-alga or blue-green-algae or blue-green-bacteria or blue-green-bacterium or blue-green-algae or Cyanobacteria or Cyanophyceae or Cyanophyta or gram-negative-oxygenic-photosynthetic-bacteria or ciguatera or histamine-fish-poisoning or histamine-food-poisoning or scombroid or scombroid-poisoning or scombroid-fish-intoxication or scombroid-fish-poisoning or scombroid-food-poisoning or scombroid-ichthyotoxicosis or scombroid-intoxication or scombroid-syndrome or scombrotoxic-fish-poisoning or scombrotoxic-poisoning or scombrotoxin-fish-poisoning or scombrotoxin-poisoning or scombrotoxism or domoic-acid or domoate or hepatitis-A or epidemic-hepatitis-virus or HAV or Hepatitis-virus-A or Hepatovirus-A or infectious-hepatitis-virus or bacillus-paratyphi-alcaligenes or bacillus-paratyphosus or alkalescens-dispar-group or Bacillus-coli or Bacillus-escherichii or Bacterium-coli or Bacterium-coli-commune or bacterium-E3 or coli-bacillus or coli-bacterium or colibacillus or colon-bacillus or E-coli or E-coli or E-coli or Enterococcus-coli or Escherichia-alkalescens-dispart or Escherichia-coli-0100 or Escherichia-coli-0124 or Escherichia-coli-8 or Escherichia-coli-strain or Escherichia-coli-suspension).ti,ab,kw. |
| 9 | or/7-8 |
| 10 | exp Mental Disease/ or exp Cognition/ or exp metacognition/ or exp Depression/ or exp Dementia/ or exp anxiety/ or exp Psychomotor Performance/ or exp motor performance/ or exp Executive Function/ or exp Child Behavior Disorder/ or exp developmental disorder/ or exp autism/ or exp language processing/ or exp language delay/ or exp Child Development/ or exp developmental delay/ or exp motor skill/ or exp Problem Solving/ or exp academic achievement/ or exp mental health/ or exp mental function/ or exp Congenital Disorder/ or exp congenital malformation/ or exp Mortality/ or exp Morbidity/ or exp chronic disease/ or exp environmental disease/ or exp child nutrition/ or exp low birth weight/ or exp Irritability/ or exp Shyness/ or exp Tremor/ or exp blindness/ or exp hearing impairment/ or exp Memory/ or exp Attention/ or exp verbal memory/ or exp Acrodynia/ or exp Kidney disease/ or exp Hypertension/ or exp tubular dysfunction/ or exp malignant neoplasm/ or exp gross motor/ or exp ataxia/ or exp fine motor/ or exp learning disorder/ or exp reading/ or exp peripheral neuropathy/ or exp neuropathy/ or exp weakness/ or exp areflexia/ or exp paresthesia/ or exp motor coordination/ or exp functional disability/ or exp visual impairment/ or exp behavior disorder/ or exp school readiness/ or exp sleep disorder/ or exp asperger syndrome/ or exp Cardiovascular disease/ or exp heart death/ or exp Neoplasm/ or exp Hypersensitivity/ or exp non insulin dependent diabetes mellitus/ or exp Asthma/ or exp allergy/ or exp atopy/ or exp Central nervous system/ or exp Immune function/ or exp cell proliferation/ or exp cell differentiation/ or exp apoptosis/ or exp Fracture/ or exp falling/ or exp thyroid gland/ or exp thyroxine/ or liothyronine/ or exp goiter/ or exp growth disorder/ or exp blood pressure/ or exp hypotension/ or exp muscle weakness/ or exp oxidative stress/ or exp immunity/ or exp health hazard/ or exp risk factor/ or exp risk assessment/ or exp outcome assessment/ or exp infection/ or exp sexual maturity/ or exp Sexual maturation/ or exp puberty/ or exp thyroid diseases/ or exp thyroid disease/ or exp prematurity/ or exp sensitization/ or exp Inflammation/ or exp Autoimmunity/ or exp Autoimmune Disease/ or exp Brain/ or exp attention deficit hyperactivity disorder/ or exp Alzheimer disease/ or exp behavior disorder/ or exp blindness/ or exp malignant neoplasm/ or exp heart death/ or exp congenital disorder/ or exp coronary artery disease/ or exp growth/ or exp child growth/ or exp "toxicity and intoxication"/ or exp biological marker/ or exp cholesterol/ or exp low density lipoprotein cholesterol/ or exp triacylglycerol/ or exp high density lipoprotein/ or exp failure to thrive/ or exp malnutrition/ or exp protein deficiency/ or exp dyslipidemia/ |
| 11 | (mental disorder or mental disorders or cognitive or neurocognitive or neurodevelop* or neurological or Alzheimer* or senility or senile or presenile or motor skills or attention-deficit-disorder or attention-deficit-disorders or ADHD or Autism-Spectrum-Disorder or Asperger or academic performance or academic failure or academic success or mental process* or congenital abnormality or congenital abnormalities or birth defects or birth defect or chronic condition* or changes-in-vision or vision problem or changes-in-hearing or deafness or gastrointestinal or respiratory or obstruction-of-visual-field or pink disease or renal disease* or tubular injury or cancer or learning disabilit* or communication* or social-interactive-skills or functional disabilities or functional disability or sensorineural defect or sensorineural defects or vision loss or hearing loss or psychological disorder* or behavioral disorder* or Asperge* or coronary disease* or cardiac death or Growth-and-development or diabetes-mellitus-Type-2 or Metabolic outcome* or accidental fall* or thyroid* or triiodothyronine or mental-growth-retardation or immune-system-phenomena or hazardous substances or hazardous substance or risk factors or risk factor or risk-benefit or thyroid dysfunction or thyroid defect or thyroid defects or pre-term or neurodevelopmental disorder* or cardiovascular disease* or toenail biomarker or neurotoxicity syndrome* or academic achievement* or acrodynia or allergy or anxiety or apoptosis or areflexia or asthma or ataxia or atopy or attention or autism or autoimmune disease* or autoimmunity or blood pressure or cell differentiation or cell proliferation or central nervous system or child behavior disorder* or child behaviour disorder* or child development or child nutritional physiological phenomena or child nutritional physiological phenomena or chronic disease* or cognition or dementia or depression or developmental delay* or developmental disorder* or environmental disease or disorders-of-environmental-origin or executive function or fine motor or bone-fracture* or fractures-bone or goiter or gross motor or health hazard or hypersensitivity or hypertension or hypotension or immune function or immune system phenomena or immunity or inflammation or irritability or kidney disease or language delay* or language processing or low birth weight or memory or mental growth retardation or mental health or metacognition or morbidity or mortality or motor coordination or muscle weakness or neoplasms or neuropathy or outcome assessment or oxidative stress or paresthesia or prematurity or premature birth or problem solving or psychomotor performance or puberty or reading or risk assessment* or school readiness or sensitization or sexual maturation or sexual maturity or shyness or sleep disorder* or social interactive skill* or tremor or tubular dysfunction or tubular injury or verbal memory or vision loss or vision problem or weakness or 3-hydroxy-5-cholestene or 3beta-hydroxy-5-cholestene or 3beta-hydroxycholest-5-ene or 5-cholesten-3beta-ol or beta-cholesterol or cholest-5-en-3beta-ol or cholest-5-ene-3-ol or cholesterin or cholesterine or cholesterol-release or dythol or nsc-8798 or cholesterol-LDL- or LDL-cholesterol or lipoproteins-LDL-cholesterol or acylglycerol-tri or fatty-acid-triglyceride or triacyl-glyceride or triglyceride or triglycerides- or tryglyceride or alpha-7-lipoprotein or alpha-lipoprotein or HDL or high-density-lipoprotein-phospholipid or lipoprotein-alpha or lipoprotein-high-density or lipoproteins-hdl or pre-alpha-lipoprotein or very-high-density-lipoprotein or failure-to-thrive or deficient-nutrition or malnourishment or severe-acute-malnutrition- or underfeeding or undernourishment or undernutrition or deficiency-protein or dietary-protein-deficiency or dyslipaemia or dyslipemia or dyslipidaemia or dyslipidaemias or dyslipidemias- or lipidaemia-dys or lipidemia-dys).ti,ab,kw. |
| 12 | or/10-11 |
| 13 | 3 and 6 and 9 and 12 |
| 14 | 13 not ((exp animal/ or exp invertebrate/ or nonhuman/ or animal experiment/ or animal tissue/ or animal model/ or exp plant/ or exp fungus/) not (exp human/ or human tissue/)) |
| 15 | limit 14 to (english language and yr="2000 -Current") |
| 16 | 15 and (article/ or article in press/) |
| 17 | 16 not (conference abstract/ or conference review/ or conference paper/ or editorial/ or erratum/ or letter/ or note/ or review/ or systematic review/ or meta analysis/) |
| 18 | exp Macaca/ or Haplorhini/ or Catarrhini/ or exp Platyrrhini/ or exp Gorilla/ or exp Pan paniscus/ or exp Pan troglodytes/ or hominid/ or exp chimpanzee/ or exp orangutan/ or exp Cercopithecidae/ or exp Hylobatidae/ or exp tarsiiform/ or exp prosimian/ or primate/ |
| 19 | (ape* or macaca* or macaque* or allenopithecus or allocebus or alouatta or alouattinae or angwantibo* or anthropoid or anthropoidea or anthropoids or aotes or aotidae or aotinae or aotus or ape or apes or arctocebus or ateles or atelidae or atelinae or avahi or aye-aye* or baboon or baboons or bonobo or bonobos or brachyteles or bushbabies or bushbaby or cacajao or callibella or callicebinae or callicebus or callimico or callithrichid* or callithrichinae or callithrix or callitrichid or callitrichidae or callitrichide or callitrichids or callitrichinae or capuchin or capuchins or carlito syrichta or catarhine* or catarhini or catarrhina or catarrhine* or catarrhini or cebid or cebidae or cebids or cebinae or ceboidea or cebuella or cebus or cephalopachus or cercocebus or cercopithecid* or cercopithecinae or cercopithecine* or cercopithecini or cercopithecoid or cercopithecoidea or cercopithecoids or cercopithecus or cheirogaleidae or cheirogaleus or cheracebus or chimp or chimpanzee or chimpanzees or chimps or chiromyiformes or chiropotes or chlorocebus or colobidae or colobinae or colobine* or colobini or colobus* or cynomolgus or daubentonia or daubentoniidae or douc or doucs or erythrocebus or eulemur or euoticus or euprimate* or galagid* or galago or galagoides or galagonidae or galagos or gelada or geladas or gibbon or gibbons or gorilla or gorillas or grivet or grivets or guenon* or guereza* or hapalemur or haplorhine* or haplorhini or haplorrhine* or haplorrhini or hominid* or hominin or homininae or hominine or hominines or hominini or hominins or hominoidea or hoolock or howler* or hylobates or hylobatidae or indri or indridae or indriid* or indris or kipunji* or lagothrix or langur or langurs or lemur or lemurid* or lemuriform or lemuriformes or lemuriforms or lemurinae or lemuroidea or lemurs or leontideus or leontocebus or leontopithecus or lepilemur or lepilemurid* or lesula* or lophocebus or loriform or loriformes or lorinae or loris or lorises or lorisid* or lorisiform* or lorisinae or lorisoid* or lutung or lutungs or macaca or macaque's or macaque or macaques or malbrouck* or mandrill or mandrills or mandrillus or mangabey* or marmoset or marmosets or mico argentatus or mico chrysoleucos or mico emiliae or mico humilis or mico marcai or mico melanurus or mico rondoni or microcebus or miopithecus or mirza coquereli or mirza zaza or monkey or monkeys or muriqui* or nasalis larvatus or nomascus or nycticebus or oedipomidas or orang utan* or orang-utan* or orangutan* or oreonax or otolemur or pan paniscus or pan troglodytes or panin or panina or panins or papio or papionini or paragalago or perodicticinae or perodicticus or phaner or piliocolobus or pithecia or pithecidae or pitheciid* or pitheciinae or pithecinae or platyrhine* or platyrhini or platyrrhina or platyrrhine* or platyrrhini or plecturocebus or pongid* or ponginae or pongo or potto or pottos or presbytini or presbytis or primate or primates or procolobus or prolemur or propithecus or prosimian* or prosimii or pseudopotto or pygathrix or rhinopithecus or rungwecebus or saguinus or saimiri or saimiriinae or sapajus or sciurocheirus or semnopithecus or siamang or siamangs or sifaka or sifakas or simians or simias or simiiform* or strepsir* or surili* or symphalangus or talapoin* or tamarin or tamarins or tamarinus or tarsier or tarsiers or tarsiid* or tarsiiform* or tarsius or theropithecus or trachypithecus or uacari* or uakari or uakaris or varecia or vervet*).ti,ab,kw. |
| 20 | or/18-19 |
| 21 | 13 and 20 |
| 22 | limit 21 to (english language and yr="2000 -Current") |
| 23 | 22 and (article/ or article in press/) |
| 24 | 23 not (conference abstract/ or conference review/ or conference paper/ or editorial/ or erratum/ or letter/ or note/ or review/ or systematic review/ or meta analysis/) |
| 25 | limit 24 to yr="2023 -Current" |
| 26 | (202311* or 202312* or 2024*).dc. and 25 |
| 27 | (2023051* or 2023052* or 2023053*).dc. and 25 |
| 28 | (202306* or 202307* or 202308* or 202309*).dc. and 25 |
| 29 | or/26-28 |
|  | **160 results** |

Database 3

Database: CENTRAL

Provider: Wiley

Search completed September 6, 2024

Limits: English

Search terms:

| 1 | (seafood or sea foods or sea food or sea food or sea foods or fish consumption or fishes or fish protein or fish proteins or fish product or fish products or fish meal or fish flour or fatty fish or shellfish or shellfish proteins or mercurialism or mercury poisoning or methylmercury or sharks or swordfish or tuna or salmonine or salmon or sardine* or sardines or gadiformes or pollock or flounder or cod or tilapia or shrimp or oyster or oysters or clams or scallops or crab or crabs or perciformes or mackerel or catfishes or trout or lobster or decapodiformes or squid or halibut or mahi mahi or crayfish or crawfish or anchovy or herring or rockfish or marine product or marine products or marlin or orange roughy or tile fish or whales or perch or walleye fish or lake trout or salmonid or catfish or sushi or cerviche or sashimi or gravlax or tuna tartare or seafood crudo or fluke crudo or fluke or carpaccio or e la ota or poke or hinava or gohu ikan or esqueixada or kelaguen or namero or kilawin or stroganina or yusheng or koi pla or kokoda or kuai or lakerda or larb pla or ota ika or tiradito or xato or umai or salmo salar or atlantic salmon or salmonids) |
| --- | --- |
| 2 | (diet or dietary supplement or dietary supplements or food or nutritional status or iron or folic acid or calcium or Zinc or phytate or phytic acid or polyphenol or polyphenols or fish species or fish tissue or fish intake or fish rich diet or fatty acids or fatty acid or fish protein or mediterranean diet or omega 3 fatty acids or omega 3 fatty acids or omega 3 fatty acid or omega 3 fatty acid or fish consumption or eicosapentaenoic acid or docosahexaenoic acid or docosapentaenoic acid or meals or food intake or dietary intake or magnesium) |
| 3 | (polyfluoroalkyl substance* or pfas or methylmercury compounds or methylmercury toxicity or organomercury compound or organic mercury compound or mercury exposure* or organomercury or methylmercury induced cytotoxicity or organomercurials or mercurial* or polychlorinated biphenyl or polychlorinated biphenyls or polychlorobiphenyl compounds or methylmercury or methyl mercury or methyl mercury or mehg or monomethylmercury or methylquecksilber or dioxin or dioxins or inorganic mercury or pfos or pfoa or campheclor or toxaphene or dichlorodiphenyltrichlorethane or chlordane or chlordan or dieldrin or hexachlorobenzene or cyclohexane or brominated flame retardant* or diphenyl ether or diphenyl ethers or polybrominated diphenyl ether or polybrominated diphenyl ethers or aquatic toxicology or aquatic toxicity or aldrin or arsenic or dibenzofuran* or endrin or fluorocarbon* or furan* or heptachlor or microfiber* or microplastic* or nanofiber* or nanoplastic* or organic fluorine* or polyfluoroalkyl substance* or total organic fluorine or cadmium or Chlorpyrifos or lorsban or dursban or Algal Bloom Harmful or Algal Blooms Harmful or Bloom Harmful Algal or Blooms Harmful Algal or Harmful Algal Blooms or Red Tide or Red Tides or Tide Red or Tides Red or Cyanobacteria or Bacteria Blue Green or Bacteria Blue Green or Blue Green Bacteria or Algae Blue Green or Algae Blue Green or Blue Green Algae or Blue Green Algae or Cyanophyceae or Blue Green Bacteria or Ciguatera or Ciguatera Poisonings or Poisoning Ciguatera or Poisonings Ciguatera or Ciguatera Fish Poisoning or Ciguatera Fish Poisonings or Poisoning Ciguatera Fish or Poisonings Ciguatera Fish or Ciguater or Scombroid or Domoic acid or Hepatitis A viruses or hepatitis A virus or Salmonella) |
| 4 | (mental disorder or mental disorders or cognitive or neurocognitive or neurodevelop* or neurological or Alzheimer* or senility or senile or presenile or motor skills or attention deficit disorder or attention deficit disorders or ADHD or Autism Spectrum Disorder or Asperger or academic performance or academic failure or academic success or mental process* or congenital abnormality or congenital abnormalities or birth defects or birth defect or chronic condition* or changes in vision or vision problem or changes in hearing or deafness or gastrointestinal or respiratory or obstruction of visual field or pink disease or renal disease* or tubular injury or cancer or learning disabilit* or communication* or social interactive skills or functional disabilities or functional disability or sensorineural defect or sensorineural defects or vision loss or hearing loss or psychological disorder* or behavioral disorder* or Asperge* or coronary disease* or cardiac death or Growth and development or diabetes mellitus Type 2 or Metabolic outcome* or accidental fall* or thyroid* or triiodothyronine or mental growth retardation or immune system phenomena or hazardous substances or hazardous substance or risk factors or risk factor or risk benefit or thyroid dysfunction or thyroid defect or thyroid defects or pre term or neurodevelopmental disorder* or cardiovascular disease* or toenail biomarker or neurotoxicity syndrome* or academic achievement* or acrodynia or allergy or anxiety or apoptosis or areflexia or asthma or ataxia or atopy or attention or autism or autoimmune disease* or autoimmunity or blood pressure or cell differentiation or cell proliferation or central nervous system or child behavior disorder* or child behaviour disorder* or child development or child nutritional physiological phenomena or child nutritional physiological phenomena or chronic disease* or cognition or dementia or depression or developmental delay* or developmental disorder* or environmental disease or disorders of environmental origin or executive function or fine motor or bone fracture* or fractures bone or goiter or gross motor or health hazard or hypersensitivity or hypertension or hypotension or immune function or immune system phenomena or immunity or inflammation or irritability or kidney disease or language delay* or language processing or low birth weight or memory or mental growth retardation or mental health or metacognition or morbidity or mortality or motor coordination or muscle weakness or neoplasms or neuropathy or outcome assessment or oxidative stress or paresthesia or prematurity or premature birth or problem solving or psychomotor performance or puberty or reading or risk assessment* or school readiness or sensitization or sexual maturation or sexual maturity or shyness or sleep disorder* or social interactive skill* or tremor or tubular dysfunction or tubular injury or verbal memory or vision loss or vision problem or weakness or Failure to thrive or malnutrition or protein deficiency or Dyslipidemias or cholesterol or Low Density Lipoprotein Cholesterol or beta Lipoprotein Cholesterol or Cholesterol beta Lipoprotein or beta Lipoprotein Cholesterol or LDL Cholesterol or Cholesteryl Linoleate LDL or LDL Cholesteryl Linoleate or triglycerides or Triacylglycerols or Triacylglycerol or Triglyceride or HDL Lipoproteins or High Density Lipoprotein or Lipoprotein High Density or High Density Lipoproteins or High Density Lipoproteins or Lipoproteins High Density or alpha Lipoproteins or alpha Lipoproteins or Heavy Lipoproteins or Lipoproteins Heavy or High Density Lipoprotein or Density Lipoprotein High or Lipoprotein High Density or alpha Lipoprotein or alpha Lipoprotein or alpha 1 Lipoprotein) |
| 5 | 1 and 2 and 3 and 4 |

### **Supplementary Table 3**: Inclusion and exclusion criteria for a systematic review about relationships between seafood consumption during childhood and adolescence and neurocognitive development

| **Category** | **Inclusion Criteria** | **Exclusion Criteria** | |
| --- | --- | --- | --- |
| Population | Individuals living in countries ranked as high or very high on the human development index during the study.     - Exposed population: Individuals in the general population who are infants, children, or adolescents up to age 18 years. Subgroups of interest:   - By race/ethnicity   - By income   - By cumulative exposure to non-chemical and environmental stressors (e.g., stress, depression, neighborhood or locale, food security)   - By pre-existing disease burden - Outcome population: Children and adolescents (up to age 18 years). Subgroups of interest:   - Infants (ages 0 to 12 months)   - Toddlers (ages 1 to 3 years)   - Early childhood (ages 4 to 8 years)   - Puberty (ages 9 to 13 years)   - Adolescents (ages 14 to 18 years) | Studies exclusively of participants with a chronic condition, hospitalized with an illness or injury. Examples include:   - Diabetes (not including gestational diabetes) - Cancer - Cardiometabolic disorders - Chronic kidney disease - Malabsorption (any disorder that causes malabsorption from the gastrointestinal tract) - Asthma | |
| Exposure | Seafood consumption:   - Types (e.g., salmon, tuna, bass) - Sources (e.g., sea, fresh water, farmed, canned, wild) - Amount (e.g., ounces per day, grams per meal) - Frequency (e.g., daily, twice a week) - Duration (e.g., length of time consuming seafood) - Preparation (e.g., fried, baked) - Timing (e.g., by trimester, age) | - Supplements - Infant formula | |
| Comparator | - Different types, sources, amounts, frequencies, durations, preparations, or timings of seafood consumption      - No seafood consumption | No comparator | |
| Outcome | Neurodevelopment and Neurodevelopmental Disorders:   - Development categories: cognition, language/communication, movement/physical, social-emotional* - Social/emotional outcomes* - Academic performance - Autism spectrum disorders (ASD) - Anxiety - Depression - Attention deficit hyperactivity disorder (ADHD) |  | |
| Study Designs | - Randomized controlled trials - Controlled (nonrandomized) trials - Cohort (observational) studies, prospective or retrospective - Case-cohort studies | - Case reports - Studies reported in theses or conference abstracts only - Studies not reported in English - Studies without primary data, such as systematic reviews, narrative reviews, editorials, and commentaries |  |

*The term behavior was used instead of social-emotional in data synthesis and conclusions to be more encompassing of behaviors beyond social-emotional development that were captured in the assessment tools, such as the Strengths and Difficulties Questionnaire.

### **Supplementary Table 4**: Full text articles that were reviewed and excluded for a systematic review about relationships between seafood consumption during childhood and adolescence and neurocognitive development

| Article | Reason for exclusion* |
| --- | --- |
| Al Malki JS, Hussien NA, Al Malki F. Maternal toxoplasmosis and the risk of childhood autism: serological and molecular small-scale studies. BMC Pediatr. 2021 Mar 17;21(1):133. doi: 10.1186/s12887-021-02604-4. PMID: 33731054; PMCID: PMC7968291. | Intervention/exposure |
| Al-Ghannami SS, Al-Adawi S, Ghebremeskel K, Hussein IS, Min Y, Jeyaseelan L, Al-Shammakhi SM, Mabry RM, Al-Oufi HS. Randomized open-label trial of docosahexaenoic acid-enriched fish oil and fish meal on cognitive and behavioral functioning in Omani children. Nutrition. 2019 Jan;57:167-172. doi: 10.1016/j.nut.2018.04.008. Epub 2018 May 17. PMID: 30195244. | Comparator |
| Barbone F, Valent F, Pisa F, Daris F, Fajon V, Gibicar D, Logar M, Horvat M. Prenatal low-level methyl mercury exposure and child development in an Italian coastal area. Neurotoxicology. 2020 Dec;81:376-381. doi: 10.1016/j.neuro.2020.09.033. Epub 2020 Oct 14. PMID: 35623359. | Population |
| Bernstein AS, Oken E, de Ferranti S; COUNCIL ON ENVIRONMENTAL HEALTH; COMMITTEE ON NUTRITION. Fish, Shellfish, and Children's Health: An Assessment of Benefits, Risks, and Sustainability. Pediatrics. 2019 May 20:e20190999. doi: 10.1542/peds.2019-0999. Epub ahead of print. Erratum in: Pediatrics. 2019 Oct;144(4):e20192403. doi: 10.1542/peds.2019-2403. PMID: 31110165; PMCID: PMC6864235. | Study design |
| Borasio F, De Cosmi V, D'Oria V, Scaglioni S, Syren ME, Turolo S, Agostoni C, Coniglio M, Molteni M, Antonietti A, Lorusso ML. Associations between Dietary Intake, Blood Levels of Omega-3 and Omega-6 Fatty Acids and Reading Abilities in Children. Biomolecules. 2023 Feb 15;13(2):368. doi: 10.3390/biom13020368. PMID: 36830737; PMCID: PMC9952928. | Intervention/exposure |
| Breider S, Hoekstra PJ, Wardenaar KJ, van den Hoofdakker BJ, Dietrich A, de Bildt A. Early-Life Environmental and Child Factors Associated with the Presence of Disruptive Behaviors in Seven-Year-Old Children with Autistic Traits in the Avon Longitudinal Study of Parents and Children. J Autism Dev Disord. 2022 Jun;52(6):2747-2761. doi: 10.1007/s10803-021-05081-x. Epub 2021 Jul 10. PMID: 34247301; PMCID: PMC9114014. | Intervention/exposure |
| Broś-Konopielko M, Białek A, Johne M, Czajkowski K. Increased LC PUFA Levels in the Serum of Pregnant Women and Their Children as a Result of Dietary Supplementation with 'Omega' Fatty Acids. Nutrients. 2023 Jan 2;15(1):231. doi: 10.3390/nu15010231. PMID: 36615888; PMCID: PMC9824740. | Intervention/exposure |
| Buckland G, Northstone K, Emmett PM, Taylor CM. Adherence to UK dietary guidelines in school-aged children from the Avon Longitudinal Study of Parents and Children (ALSPAC) cohort. Br J Nutr. 2023 Aug 14;130(3):454-466. doi: 10.1017/S0007114522003336. Epub 2022 Oct 28. PMID: 36305030; PMCID: PMC10331434. | Outcome |
| Canals-Sans J, Esteban-Figuerola P, Morales-Hidalgo P, Arija V. Do Children with Autism Spectrum Disorders Eat Differently and Less Adequately than Those with Subclinical ASD and Typical Development? EPINED Epidemiological Study. J Autism Dev Disord. 2022 Jan;52(1):361-375. doi: 10.1007/s10803-021-04928-7. Epub 2021 Mar 21. PMID: 33745113. | Population |
| Carvalho C, Correia D, Severo M, Afonso C, Bandarra NM, Gonçalves S, Lourenço HM, Dias MG, Oliveira L, Nabais P, Carmona P, Monteiro S, Borges M, Lopes C, Torres D. Quantitative risk-benefit assessment of Portuguese fish and other seafood species consumption scenarios. Br J Nutr. 2022 Nov 28;128(10):1997-2010. doi: 10.1017/S0007114521004773. Epub 2021 Dec 7. PMID: 34872627. | Population |
| Cediel Ulloa A, Gliga A, Love TM, Pineda D, Mruzek DW, Watson GE, Davidson PW, Shamlaye CF, Strain JJ, Myers GJ, van Wijngaarden E, Ruegg J, Broberg K. Prenatal methylmercury exposure and DNA methylation in seven-year-old children in the Seychelles Child Development Study. Environ Int. 2021 Feb;147:106321. doi: 10.1016/j.envint.2020.106321. Epub 2020 Dec 16. PMID: 33340986. | Outcome |
| Chen T, Li Y, Liu J, Wang Y, Wei S. The burden of mild intellectual disability attributed to prenatal exposure to methylmercury in China, 2017. Ecotoxicol Environ Saf. 2023 Apr 1;254:114748. doi: 10.1016/j.ecoenv.2023.114748. Epub 2023 Mar 13. PMID: 36921496. | Intervention/exposure |
| Clarkson TW, Strain JJ. Methyl mercury: loaves versus fishes. Neurotoxicology. 2020 Dec;81:282-287. doi: 10.1016/j.neuro.2020.09.018. Epub 2020 Oct 14. PMID: 35587135. | Study design |
| Collet OA, Heude B, Forhan A, Delcourt C, Orri M, Van der Waerden J, Melchior M, Côté S, Lioret S, de Lauzon-Guillain B, Galéra C. Prenatal Diet and Children's Trajectories of Anxiety and Depression Symptoms from 3 to 8 Years: The EDEN Mother-Child Cohort. J Nutr. 2021 Jan 4;151(1):162-169. doi: 10.1093/jn/nxaa343. PMID: 33296456. | Population |
| Conway MC, Yeates AJ, Love TM, Weller D, McSorley EM, Mulhern MS, Wesolowska M, Watson GE, Myers GJ, Shamlaye CF, Henderson J, Davidson PW, van Wijngaarden E, Strain JJ. Maternal fish consumption and child neurodevelopment in Nutrition 1 Cohort: Seychelles Child Development Study. Br J Nutr. 2023 Oct 28;130(8):1366-1372. doi: 10.1017/S0007114523000375. Epub 2023 Feb 10. PMID: 36759019; PMCID: PMC10511674. | Population |
| Dadang, R., Silmi Dzakiyyah, M., Widartika, W., Maryati, D., Rahmat, M., Surmita, S., Pusparini, P., Agung, F., Ashri Nurfurqon, F., Hamzah, A., & Mulyo, G.P. (2021). The Provision of Carrot Catfish Dumplings Affects the Weight of Elementary School Students with Low Nutritional Status. Open Access Macedonian Journal of Medical Sciences. | Outcome |
| Darcey VL, McQuaid GA, Fishbein DH, VanMeter JW. Dietary Long-Chain Omega-3 Fatty Acids Are Related to Impulse Control and Anterior Cingulate Function in Adolescents. Front Neurosci. 2019 Jan 9;12:1012. doi: 10.3389/fnins.2018.01012. PMID: 30686978; PMCID: PMC6333752. | Intervention/exposure |
| Darcey VL, Serafine KM. Omega-3 Fatty Acids and Vulnerability to Addiction: Reviewing Preclinical and Clinical Evidence. Curr Pharm Des. 2020;26(20):2385-2401. doi: 10.2174/1381612826666200429094158. PMID: 32348211. | Population |
| de Lauzon-Guillain B, Marques C, Kadawathagedara M, Bernard JY, Tafflet M, Lioret S, Charles MA. Maternal diet during pregnancy and child neurodevelopment up to age 3.5 years: the nationwide Étude Longitudinale Française depuis l'Enfance (ELFE) birth cohort. Am J Clin Nutr. 2022 Oct 6;116(4):1101-1111. doi: 10.1093/ajcn/nqac206. PMID: 35918250. | Population |
| de Paula HK, Love TM, Pineda D, Watson GE, Thurston SW, Yeates AJ, Mulhern MS, McSorley EM, Strain JJ, Shamlaye CF, Myers GJ, Rand MD, van Wijngaarden E, Broberg K. KEAP1 polymorphisms and neurodevelopmental outcomes in children with exposure to prenatal MeHg from the Seychelles Child Development Study Nutrition Cohort 2. Neurotoxicology. 2023 Dec;99:177-183. doi: 10.1016/j.neuro.2023.10.008. Epub 2023 Oct 17. PMID: 37858899; PMCID: PMC10841683. | Intervention/exposure |
| Desrochers-Couture M, Courtemanche Y, Forget-Dubois N, Bélanger RE, Boucher O, Ayotte P, Cordier S, Jacobson JL, Jacobson SW, Muckle G. Association between early lead exposure and externalizing behaviors in adolescence: A developmental cascade. Environ Res. 2019 Nov;178:108679. doi: 10.1016/j.envres.2019.108679. Epub 2019 Aug 19. PMID: 31454729; PMCID: PMC6759380. | Intervention/exposure |
| Fahmida U, Hidayat AT, Oka AASI, Suciyanti D, Pathurrahman P, Wangge G. Effectiveness of an Integrated Nutrition Rehabilitation on Growth and Development of Children under Five Post 2018 Earthquake in East Lombok, Indonesia. Int J Environ Res Public Health. 2022 Feb 28;19(5):2814. doi: 10.3390/ijerph19052814. PMID: 35270504; PMCID: PMC8910115. | Intervention/exposure |
| Flor-Alemany M, Baena-García L, Migueles JH, Henriksson P, Löf M, Aparicio VA. Associations of Mediterranean diet with psychological ill-being and well-being throughout the pregnancy course: The GESTAFIT project. Qual Life Res. 2022 Sep;31(9):2705-2716. doi: 10.1007/s11136-022-03121-2. Epub 2022 Mar 16. PMID: 35297499; PMCID: PMC9356938. | Population |
| Frost B, Martin CR, Calkins KL. Dilemmas in the delivery of intravenous lipid emulsions and approach to hypertriglyceridemia in very preterm and low birth weight infants. J Perinatol. 2023 Sep;43(9):1189-1193. doi: 10.1038/s41372-023-01637-0. Epub 2023 Apr 8. PMID: 37031340. | Intervention/exposure |
| Fruh V, Rifas-Shiman SL, Coull BA, Devick KL, Amarasiriwardena C, Cardenas A, Bellinger DC, Wise LA, White RF, Wright RO, Oken E, Claus Henn B. Prenatal exposure to a mixture of elements and neurobehavioral outcomes in mid-childhood: Results from Project Viva. Environ Res. 2021 Oct;201:111540. doi: 10.1016/j.envres.2021.111540. Epub 2021 Jun 21. PMID: 34166661; PMCID: PMC8502495. | Intervention/exposure |
| Gallini F, Pelosi MS, De Rose DU, Coppola M, Costa S, Romeo DM, Cocca C, Maggio L, Cota F, Piersanti A, Ricci D, Vento G. Neurodevelopmental Outcomes in Preterm Infants Receiving a Multicomponent vs. a Soybean-Based Lipid Emulsion: 24 Month Follow-Up of a Randomized Controlled Trial. Nutrients. 2022 Dec 23;15(1):58. doi: 10.3390/nu15010058. PMID: 36615716; PMCID: PMC9824491. | Intervention/exposure |
| Gignac F, Romaguera D, Fernández-Barrés S, Phillipat C, Garcia Esteban R, López-Vicente M, Vioque J, Fernández-Somoano A, Tardón A, Iñiguez C, Lopez-Espinosa MJ, García de la Hera M, Amiano P, Ibarluzea J, Guxens M, Sunyer J, Julvez J. Maternal nut intake in pregnancy and child neuropsychological development up to 8 years old: a population-based cohort study in Spain. Eur J Epidemiol. 2019 Jul;34(7):661-673. doi: 10.1007/s10654-019-00521-6. Epub 2019 May 7. PMID: 31062119. | Intervention/exposure |
| Gonzalez-Nahm S, Marchesoni J, Maity A, Maguire RL, House JS, Tucker R, Atkinson T, Murphy SK, Hoyo C. Maternal Mediterranean Diet Adherence and Its Associations with Maternal Prenatal Stressors and Child Growth. Curr Dev Nutr. 2022 Nov 15;6(11):nzac146. doi: 10.1093/cdn/nzac146. PMID: 36406812; PMCID: PMC9665863. | Outcome |
| Gustafsson HC, Dunn GA, Mitchell AJ, Holton KF, Loftis JM, Nigg JT, Sullivan EL. The association between heightened ADHD symptoms and cytokine and fatty acid concentrations during pregnancy. Front Psychiatry. 2022 Jul 22;13:855265. doi: 10.3389/fpsyt.2022.855265. PMID: 35935444; PMCID: PMC9353213. | Intervention/exposure |
| Hamazaki K, Matsumura K, Tsuchida A, Kasamatsu H, Tanaka T, Ito M, Inadera H; Japan Environment and Children's Study Group. Maternal dietary intake of fish and PUFAs and child neurodevelopment at 6 months and 1 year of age: a nationwide birth cohort-the Japan Environment and Children's Study (JECS). Am J Clin Nutr. 2020 Nov 11;112(5):1295-1303. doi: 10.1093/ajcn/nqaa190. PMID: 32766751; PMCID: PMC7657336. | Population |
| Harris HA, Mou Y, Dieleman GC, Voortman T, Jansen PW. Child Autistic Traits, Food Selectivity, and Diet Quality: A Population-Based Study. J Nutr. 2022 Mar 3;152(3):856-862. doi: 10.1093/jn/nxab413. PMID: 34871440; PMCID: PMC8891181. | Population |
| Harrison TM, Brown R, Bonny AE, Manos BE, Bravender T. Omega-3 fatty acids and autonomic function in adolescents with anorexia: A randomized trial. Pediatr Res. 2022 Oct;92(4):1042-1050. doi: 10.1038/s41390-022-02058-z. Epub 2022 Jul 28. PMID: 35902705. | Intervention/exposure |
| He XJ, Dai RX, Tian CQ, Hu CL. Neurodevelopmental outcome at 1 year in offspring of women with gestational diabetes mellitus. Gynecol Endocrinol. 2021 Jan;37(1):88-92. doi: 10.1080/09513590.2020.1754785. Epub 2020 Apr 21. PMID: 32314619. | Intervention/exposure |
| Hulkkonen P, Kataja EL, Vahlberg T, Koivuniemi E, Houttu N, Pellonperä O, Mokkala K, Karlsson H, Laitinen K. The efficacy of probiotics and/or n-3 long-chain polyunsaturated fatty acids intervention on maternal prenatal and postnatal depressive and anxiety symptoms among overweight and obese women. J Affect Disord. 2021 Jun 15;289:21-30. doi: 10.1016/j.jad.2021.04.006. Epub 2021 Apr 20. PMID: 33930612. | Population |
| Jaramillo-Ospina AM, Roman GT, Rodrigues DM, Patel S, Pokhvisneva I, Chakr VG, Levitan RD, Meaney MJ, Silveira PP. Omega-3 polygenic score protects against altered eating behavior in intrauterine growth-restricted children. Pediatr Res. 2023 Sep;94(3):1225-1234. doi: 10.1038/s41390-023-02609-y. Epub 2023 May 4. PMID: 37142650. | Outcome |
| Jaureguiberry MS, Venturino A. Nutritional and environmental contributions to autism spectrum disorders: Focus on nutrigenomics as complementary therapy. Int J Vitam Nutr Res. 2022 Jul;92(3-4):248-266. doi: 10.1024/0300-9831/a000630. Epub 2020 Feb 17. PMID: 32065556. | Intervention/exposure |
| Jung TH, Hwang HJ, Han KS. Correlation of attention deficit hyperactivity disorder with gut microbiota according to the dietary intake of Korean elementary school students. PLoS One. 2022 Sep 30;17(9):e0275520. doi: 10.1371/journal.pone.0275520. PMID: 36178961; PMCID: PMC9524712. | Study design |
| Katrenčíková B, Vaváková M, Waczulíková I, Oravec S, Garaiova I, Nagyová Z, Hlaváčová N, Ďuračková Z, Trebatická J. Lipid Profile, Lipoprotein Subfractions, and Fluidity of Membranes in Children and Adolescents with Depressive Disorder: Effect of Omega-3 Fatty Acids in a Double-Blind Randomized Controlled Study. Biomolecules. 2020 Oct 8;10(10):1427. doi: 10.3390/biom10101427. PMID: 33050072; PMCID: PMC7650679. | Population |
| Khayyatzadeh SS, Shafiee M, Far PE, Ziaee SS, Bagherniya M, Ebrahimi S, Boromand N, Ferns GA, Ghayour-Mobarhan M. Adherence to a healthy dietary pattern is associated with less severe depressive symptoms among adolescent girls. Psychiatry Res. 2019 Feb;272:467-473. doi: 10.1016/j.psychres.2018.12.164. Epub 2018 Dec 31. PMID: 30611966. | Study design |
| Kou X, Bulló M, Rovira J, Díaz-López A, Arija V. Dietary intake of metals, metalloids, and persistent organic pollutants in Spanish pregnant women. ECLIPSES study. Chemosphere. 2023 Dec;344:140319. doi: 10.1016/j.chemosphere.2023.140319. Epub 2023 Oct 4. PMID: 37802481. | Population |
| Kvestad I, Hysing M, Kjellevold M, Næss S, Dahl L, Markhus MW. Maternal Cod Intake during Pregnancy and Infant Development in the First Year of Life: Secondary Analyses from a Randomized Controlled Trial. J Nutr. 2021 Jul 1;151(7):1879-1885. doi: 10.1093/jn/nxab083. PMID: 33847344. | Population |
| Lach LE, Chetta KE, Ruddy-Humphries AL, Ebeling MD, Gregoski MJ, Katikaneni LD. Body Composition and "Catch-Up" Fat Growth in Healthy Small for Gestational Age Preterm Infants and Neurodevelopmental Outcomes. Nutrients. 2022 Jul 25;14(15):3051. doi: 10.3390/nu14153051. PMID: 35893903; PMCID: PMC9332383. | Intervention/exposure |
| Landrigan PJ, Stegeman JJ, Fleming LE, Allemand D, Anderson DM, Backer LC, Brucker-Davis F, Chevalier N, Corra L, Czerucka D, Bottein MD, Demeneix B, Depledge M, Deheyn DD, Dorman CJ, Fénichel P, Fisher S, Gaill F, Galgani F, Gaze WH, Giuliano L, Grandjean P, Hahn ME, Hamdoun A, Hess P, Judson B, Laborde A, McGlade J, Mu J, Mustapha A, Neira M, Noble RT, Pedrotti ML, Reddy C, Rocklöv J, Scharler UM, Shanmugam H, Taghian G, van de Water JAJM, Vezzulli L, Weihe P, Zeka A, Raps H, Rampal P. Human Health and Ocean Pollution. Ann Glob Health. 2020 Dec 3;86(1):151. doi: 10.5334/aogh.2831. PMID: 33354517; PMCID: PMC7731724. | Study design |
| Lehner A, Staub K, Aldakak L, Eppenberger P, Rühli F, Martin RD, Bender N. Fish consumption is associated with school performance in children in a non-linear way: Results from the German cohort study KiGGS. Evol Med Public Health. 2019 Dec 23;2020(1):2-11. doi: 10.1093/emph/eoz038. PMID: 31976073; PMCID: PMC6970346. | Study design |
| Lin P, Lee PI, Ling MP. Probabilistic benefit-risk analysis of fish: Nutritional benefit versus methylmercury-contaminated risk. Mar Pollut Bull. 2023 Aug;193:115179. doi: 10.1016/j.marpolbul.2023.115179. Epub 2023 Jun 22. PMID: 37352805. | Population |
| Llop S, Murcia M, Amorós R, Julvez J, Santa-Marina L, Soler-Blasco R, Rebagliato M, Iñiguez C, Aguinagalde X, Iriarte G, Lopez-Espinosa MJ, Andiarena A, Gonzalez L, Vioque J, Sunyer J, Ballester F. Postnatal exposure to mercury and neuropsychological development among preschooler children. Eur J Epidemiol. 2020 Mar;35(3):259-271. doi: 10.1007/s10654-020-00620-9. Epub 2020 Mar 13. PMID: 32170664. | Intervention/exposure |
| Love TM, Wahlberg K, Pineda D, Watson GE, Zareba G, Thurston SW, Davidson PW, Shamlaye CF, Myers GJ, Rand M, van Wijngaarden E, Broberg K. Contribution of child ABC-transporter genetics to prenatal MeHg exposure and neurodevelopment. Neurotoxicology. 2022 Jul;91:228-233. doi: 10.1016/j.neuro.2022.05.019. Epub 2022 May 30. PMID: 35654246; PMCID: PMC9723801. | Intervention/exposure |
| Lozano M, Murcia M, Soler-Blasco R, González L, Iriarte G, Rebagliato M, Lopez-Espinosa MJ, Esplugues A, Ballester F, Llop S. Exposure to mercury among 9-year-old children and neurobehavioural function. Environ Int. 2021 Jan;146:106173. doi: 10.1016/j.envint.2020.106173. Epub 2020 Oct 20. PMID: 33096466. | Outcome |
| Mahmassani HA, Switkowski KM, Johnson EJ, Scott TM, Rifas-Shiman SL, Oken E, Jacques PF. Early Childhood Lutein and Zeaxanthin Intake Is Positively Associated with Early Childhood Receptive Vocabulary and Mid-Childhood Executive Function But No Other Cognitive or Behavioral Outcomes in Project Viva. J Nutr. 2022 Nov;152(11):2555-2564. doi: 10.1093/jn/nxac188. Epub 2022 Aug 24. PMID: 36774121; PMCID: PMC9644167. | Intervention/exposure |
| Mank I, Vandormael A, Traoré I, Ouédraogo WA, Sauerborn R, Danquah I. Dietary habits associated with growth development of children aged < 5 years in the Nouna Health and Demographic Surveillance System, Burkina Faso. Nutr J. 2020 Aug 9;19(1):81. doi: 10.1186/s12937-020-00591-3. PMID: 32772913; PMCID: PMC7416397. | Country |
| Marinoni M, Giordani E, Mosconi C, Rosolen V, Concina F, Fiori F, Carletti C, Knowles A, Pani P, Bin M, Ronfani L, Ferraroni M, Barbone F, Parpinel M, Edefonti V. Are Dietary Patterns Related to Cognitive Performance in 7-Year-Old Children? Evidence from a Birth Cohort in Friuli Venezia Giulia, Italy. Nutrients. 2022 Oct 7;14(19):4168. doi: 10.3390/nu14194168. PMID: 36235820; PMCID: PMC9571625. | Intervention/exposure |
| Marinov DB, Hristova DN. Behavioral and nutritional assessment of teenagers from Varna. J of IMAB. 2021 Jan-Mar;27(1):3549-3553. doi: 10.5272/jimab.2021271.3549. | Intervention/exposure |
| Markhus MW, Hysing M, Midtbø LK, et al. Effects of Two Weekly Servings of Cod for 16 Weeks in Pregnancy on Maternal Iodine Status and Infant Neurodevelopment: Mommy's Food, a Randomized-Controlled Trial. Thyroid. 2021;31(2):288-298. doi:10.1089/thy.2020.0115 | Population |
| Matrov D, Kurrikoff T, Villa I, et al. Association of Impulsivity With Food, Nutrients, and Fitness in a Longitudinal Birth Cohort Study. Int J Neuropsychopharmacol. 2022;25(12):1014-1025. doi:10.1093/ijnp/pyac052 | Population |
| McNamara RK, Strawn JR, Tallman MJ, et al. Effects of Fish Oil Monotherapy on Depression and Prefrontal Neurochemistry in Adolescents at High Risk for Bipolar I Disorder: A 12-Week Placebo-Controlled Proton Magnetic Resonance Spectroscopy Trial. J Child Adolesc Psychopharmacol. 2020;30(5):293-305. doi:10.1089/cap.2019.0124 | Intervention/exposure |
| Medin AC, Carlsen MH, Andersen LF. Iodine intake among children and adolescents in Norway: Estimates from the national dietary survey Ungkost 3 (2015-2016). J Trace Elem Med Biol. 2020 Mar;58:126427. doi: 10.1016/j.jtemb.2019.126427. Epub 2019 Nov 6. PMID: 31770674. | Intervention/exposure |
| Mergler D, Philibert A, Fillion M, Da Silva J. The Contribution across Three Generations of Mercury Exposure to Attempted Suicide among Children and Youth in Grassy Narrows First Nation, Canada: An Intergenerational Analysis. Environ Health Perspect. 2023 Jul;131(7):77001. doi: 10.1289/EHP11301. Epub 2023 Jul 19. PMID: 37466317; PMCID: PMC10355150. | Population |
| Morandini HAE, Rao P, Hood SD, Griffiths K, Silk TJ, Zepf FD. Effects of dietary omega-3 intake on vigilant attention and resting-state functional connectivity in neurotypical children and adolescents. Nutr Neurosci. 2022 Nov;25(11):2269-2278. doi: 10.1080/1028415X.2021.1955434. Epub 2021 Aug 9. PMID: 34369315. | Intervention/exposure |
| Morton SU, Vyas R, Gagoski B, Vu C, Litt J, Larsen RJ, Kuchan MJ, Lasekan JB, Sutton BP, Grant PE, Ou Y. Maternal Dietary Intake of Omega-3 Fatty Acids Correlates Positively with Regional Brain Volumes in 1-Month-Old Term Infants. Cereb Cortex. 2020 Apr 14;30(4):2057-2069. doi: 10.1093/cercor/bhz222. PMID: 31711132; PMCID: PMC8355466. | Intervention/exposure |
| Myers GJ, Davidson PW, Shamlaye C, Cox C, Kost J, Beck C, Huang LS, Weiss B. The Seychelles Child Development Study of methyl mercury from fish consumption: analysis of subscales from the Child Behaviour Checklist at age 107 months in the main cohort. Neurotoxicology. 2020 Dec;81:331-338. doi: 10.1016/j.neuro.2020.09.025. Epub 2020 Oct 14. PMID: 35623358. | Intervention/exposure |
| Nakamura M, Tatsuta N, Murata K, Nakai K, Iwata T, Otobe T, Sakamoto M, Yamamoto M, Itatani M, Miura Y, Koriyama C. Neurodevelopmental associations of prenatal and postnatal methylmercury exposure among first-grade children in the Kinan region, Japan. Environ Res. 2023 Oct 15;235:116688. doi: 10.1016/j.envres.2023.116688. Epub 2023 Jul 17. PMID: 37467938. | Intervention/exposure |
| Nišević JR, Prpić I, Kolić I, Baždarić K, Tratnik JS, Prpić IŠ, Mazej D, Špirić Z, Barbone F, Horvat M. Combined prenatal exposure to mercury and LCPUFA on newborn's brain measures and neurodevelopment at the age of 18 months. Environ Res. 2019 Nov;178:108682. doi: 10.1016/j.envres.2019.108682. Epub 2019 Aug 20. PMID: 31450150. | Population |
| Nishi D, Su KP, Usuda K, Pei-Chen Chang J, Chiang YJ, Chen HT, Chien YC, Guu TW, Okazaki E, Hamazaki K, Susukida R, Nakaya N, Sone T, Sano Y, Ito H, Isaka K, Tachibana Y, Tanigaki S, Suzuki T, Hashimoto K, Hamazaki T, Matsuoka YJ. The Efficacy of Omega-3 Fatty Acids for Depressive Symptoms among Pregnant Women in Japan and Taiwan: A Randomized, Double-Blind, Placebo-Controlled Trial (SYNCHRO; NCT01948596). Psychother Psychosom. 2019;88(2):122-124. doi: 10.1159/000495296. Epub 2018 Dec 12. PMID: 30540990. | Population |
| Normia J, Niinivirta-Joutsa K, Isolauri E, Jääskeläinen SK, Laitinen K. Perinatal nutrition impacts on the functional development of the visual tract in infants. Pediatr Res. 2019 Jan;85(1):72-78. doi: 10.1038/s41390-018-0161-2. Epub 2018 Sep 20. PMID: 30237571. | Date of publication |
| Oliveira RAA, Pinto BD, Rebouças BH, Ciampi de Andrade D, Vasconcellos ACS, Basta PC. Neurological Impacts of Chronic Methylmercury Exposure in Munduruku Indigenous Adults: Somatosensory, Motor, and Cognitive Abnormalities. Int J Environ Res Public Health. 2021 Sep 29;18(19):10270. doi: 10.3390/ijerph181910270. PMID: 34639574; PMCID: PMC8507861. | Study design |
| Orlando MS, Love T, Harrington D, Dziorny AC, Shamlaye CF, Watson GE, van Wijngaarden E, Davidson PW, Myers GJ. The association of auditory function measures with low-level methylmercury from oceanic fish consumption and mercury vapor from amalgam: The Seychelles Child Development Study Nutrition 1 Cohort. Neurotoxicology. 2023 Mar;95:46-55. doi: 10.1016/j.neuro.2022.12.010. Epub 2023 Jan 5. PMID: 36621469; PMCID: PMC9998349. | Intervention/exposure |
| Packull-McCormick S, Ashley-Martin J, Singh K, Fisher M, Arbuckle TE, Lanphear B, Laird BD, Muckle G, Booij L, Asztalos E, Walker M, Bouchard MF, Saint-Amour D, Boivin M, Borghese M. Prenatal and concurrent blood mercury concentrations and associations with IQ in canadian preschool children. Environ Res. 2023 Sep 15;233:116463. doi: 10.1016/j.envres.2023.116463. Epub 2023 Jun 19. PMID: 37343750. | Intervention/exposure |
| Perini JA, Silva MC, Vasconcellos ACS, Viana PVS, Lima MO, Jesus IM, Kempton JW, Oliveira RAA, Hacon SS, Basta PC. Genetic Polymorphism of Delta Aminolevulinic Acid Dehydratase (ALAD) Gene and Symptoms of Chronic Mercury Exposure in Munduruku Indigenous Children within the Brazilian Amazon. Int J Environ Res Public Health. 2021 Aug 19;18(16):8746. doi: 10.3390/ijerph18168746. PMID: 34444495; PMCID: PMC8394242. | Outcome |
| Plaza-Diaz J, Flores-Rojas K, Torre-Aguilar MJ, Gomez-Fernández AR, Martín-Borreguero P, Perez-Navero JL, Gil A, Gil-Campos M. Dietary Patterns, Eating Behavior, and Nutrient Intakes of Spanish Preschool Children with Autism Spectrum Disorders. Nutrients. 2021 Oct 10;13(10):3551. doi: 10.3390/nu13103551. PMID: 34684552; PMCID: PMC8541028. | Outcome |
| Rojo-Marticella M, Arija V, Alda JÁ, Morales-Hidalgo P, Esteban-Figuerola P, Canals J. Do Children with Attention-Deficit/Hyperactivity Disorder Follow a Different Dietary Pattern than That of Their Control Peers? Nutrients. 2022 Mar 8;14(6):1131. doi: 10.3390/nu14061131. PMID: 35334788; PMCID: PMC8949924. | Population |
| Rothenberg SE, Korrick SA, Liu J, Nong Y, Nong H, Hong C, Trinh EP, Jiang X, Biasini FJ, Ouyang F. Maternal methylmercury exposure through rice ingestion and child neurodevelopment in the first three years: a prospective cohort study in rural China. Environ Health. 2021 Apr 28;20(1):50. doi: 10.1186/s12940-021-00732-z. PMID: 33910568; PMCID: PMC8082930. | Population |
| Saito H, Sekikawa T, Taguchi J, Shozawa T, Kinoshita Y, Matsumura K, Yanagihara K, Nikaido K, Urasaki S, Imaizumi H, Hatano H. Prenatal and postnatal methyl mercury exposure in Niigata, Japan: adult outcomes. Neurotoxicology. 2020 Dec;81:364-372. doi: 10.1016/j.neuro.2020.09.031. Epub 2020 Oct 14. PMID: 35587140. | Population |
| Santos-Lima CD, Mourão DS, Carvalho CF, Souza-Marques B, Vega CM, Gonçalves RA, Argollo N, Menezes-Filho JA, Abreu N, Hacon SS. Neuropsychological Effects of Mercury Exposure in Children and Adolescents of the Amazon Region, Brazil. Neurotoxicology. 2020 Jul;79:48-57. doi: 10.1016/j.neuro.2020.04.004. Epub 2020 Apr 23. PMID: 32335201. | Intervention/exposure |
| Saros L, Lind A, Setänen S, Tertti K, Koivuniemi E, Ahtola A, Haataja L, Shivappa N, Hébert JR, Vahlberg T, Laitinen K. Maternal obesity, gestational diabetes mellitus, and diet in association with neurodevelopment of 2-year-old children. Pediatr Res. 2023 Jul;94(1):280-289. doi: 10.1038/s41390-022-02455-4. Epub 2023 Jan 3. PMID: 36596942; PMCID: PMC10356612. | Population |
| Sathe, N, Gokhale, D. Intelligence Quotient and Nutritional Status of 4–6 Year Old Children from Fishermen Community of Goa, India. Indian Journal of Public Health Research & Development. 2019 Jan;10(7):1594. doi: 10.5958/0976-5506.2019.01824.2. | Study design |
| Shamlaye C, Davidson PW, Myers GJ. The Seychelles Child Development Study: two decades of collaboration. Neurotoxicology. 2020 Dec;81:315-322. doi: 10.1016/j.neuro.2020.09.023. Epub 2020 Oct 14. PMID: 35587138. | Intervention/exposure |
| Silman AK, Chhabria R, Hafzalla GW, Giffin L, Kucharski K, Myers K, Culquichicón C, Montero S, Lescano AG, Vega CM, Fernandez LE, Silman MR, Kane MJ, Sanders JW. Impairment in Working Memory and Executive Function Associated with Mercury Exposure in Indigenous Populations in Upper Amazonian Peru. Int J Environ Res Public Health. 2022 Sep 2;19(17):10989. doi: 10.3390/ijerph191710989. PMID: 36078698; PMCID: PMC9517927. | Study design |
| Sloane-Reeves J, Davidson PW, Myers GJ, Shamlaye C, Leste A, Huang LS, Thurston S. Scholastic achievement among children enrolled in the Seychelles Child Development Study. Neurotoxicology. 2020 Dec;81:347-352. doi: 10.1016/j.neuro.2020.09.027. Epub 2020 Oct 14. PMID: 33742601. | Intervention/exposure |
| Spiller P, van Wijngaarden E, Adams HR, Strain JJ, McSorley EM, Mulhern MS, Conway MC, Yeates AJ, Carrington C, Bolger PM, Morgan KM, Taylor CM, Ralston NVC, Crawford MA, Hibbeln JR, Brenna JT, Myers GJ. Net effects explains the benefits to children from maternal fish consumption despite methylmercury in fish. Neurotoxicology. 2023 Dec;99:195-205. doi: 10.1016/j.neuro.2023.10.010. Epub 2023 Oct 20. PMID: 37866693. | Intervention/exposure |
| Strain JJ, Bonham MP, Duffy EM, Wallace JMW, Robson PJ, Clarkson TW, Shamlaye C. Nutrition and neurodevelopment: the search for candidate nutrients in the Seychelles Child Development Nutrition Study. Neurotoxicology. 2020 Dec;81:300-306. doi: 10.1016/j.neuro.2020.09.021. Epub 2020 Oct 14. PMID: 33741113. | Study design |
| Strain JJ, Love TM, Yeates AJ, Weller D, Mulhern MS, McSorley EM, Thurston SW, Watson GE, Mruzek D, Broberg K, Rand MD, Henderson J, Shamlaye CF, Myers GJ, Davidson PW, van Wijngaarden E. Associations of prenatal methylmercury exposure and maternal polyunsaturated fatty acid status with neurodevelopmental outcomes at 7 years of age: results from the Seychelles Child Development Study Nutrition Cohort 2. Am J Clin Nutr. 2021 Feb 2;113(2):304-313. doi: 10.1093/ajcn/nqaa338. PMID: 33330939; PMCID: PMC7851824. | Intervention/exposure |
| Szamreta EA, Qin B, Rivera-Núñez Z, Parekh N, Barrett ES, Ferrante J, Lin Y, Bandera EV. Greater adherence to a Mediterranean-like diet is associated with later breast development and menarche in peripubertal girls. Public Health Nutr. 2020 Apr;23(6):1020-1030. doi: 10.1017/S1368980019002349. Epub 2019 Aug 23. PMID: 31439055; PMCID: PMC10071494. | Outcome |
| Thanhaeuser M, Fuiko R, Oberleitner-Leeb C, Brandstaetter S, Binder C, Thajer A, Huber-Dangl M, Haiden N, Pablik E, Berger A, Repa A. A Randomized Trial of Parenteral Nutrition Using a Mixed Lipid Emulsion Containing Fish Oil in Infants of Extremely Low Birth Weight: Neurodevelopmental Outcome at 12 and 24 Months Corrected Age, A Secondary Outcome Analysis. J Pediatr. 2020 Nov;226:142-148.e5. doi: 10.1016/j.jpeds.2020.06.056. Epub 2020 Jun 23. PMID: 32590001; PMCID: PMC7612562. | Intervention/exposure |
| Thanhaeuser M, Steyrl D, Fuiko R, Brandstaetter S, Binder C, Thajer A, Huber-Dangl M, Haiden N, Berger A, Repa A. A secondary Outcome Analysis of a Randomized Trial Using a Mixed Lipid Emulsion Containing Fish Oil in Infants with Extremely Low Birth Weight: Cognitive and Behavioral Outcome at Preschool Age. J Pediatr. 2023 Mar;254:68-74.e3. doi: 10.1016/j.jpeds.2022.10.014. Epub 2022 Oct 17. PMID: 36257349. | Intervention/exposure |
| Thanhaeuser M, Steyrl D, Fuiko R, Brandstaetter S, Binder C, Thajer A, Huber-Dangl M, Haiden N, Berger A, Repa A. Neurodevelopmental Outcome of Extremely Low Birth Weight Infants with Cholestasis at 12 and 24 Months. Neonatology. 2022;119(4):501-509. doi: 10.1159/000525003. Epub 2022 Jun 9. PMID: 35679842. | Intervention/exposure |
| Thurston SW, Myers G, Mruzek D, Harrington D, Adams H, Shamlaye C, van Wijngaarden E. Associations between time-weighted postnatal methylmercury exposure from fish consumption and neurodevelopmental outcomes through 24 years of age in the Seychelles Child Development Study Main Cohort. Neurotoxicology. 2022 Jul;91:234-244. doi: 10.1016/j.neuro.2022.05.016. Epub 2022 May 25. PMID: 35643326; PMCID: PMC9749799. | Intervention/exposure |
| Torgalkar R, Shah J, Dave S, Yang J, Ostad N, Kotsopoulos K, Unger S, Kelly E, Shah PS. Fish oil-containing multicomponent lipid emulsion vs soy-based lipid emulsion and neurodevelopmental outcomes of children born < 29 weeks' gestation. J Perinatol. 2020 Nov;40(11):1712-1718. doi: 10.1038/s41372-020-0710-5. Epub 2020 Jun 7. PMID: 32507860. | Intervention/exposure |
| Trdin A, Snoj Tratnik J, Mazej D, Fajon V, Krsnik M, Osredkar J, Prpić I, Špirić Z, Petrović O, Marc J, Neubauer D, Kodrič J, Kobal AB, Barbone F, Falnoga I, Horvat M. Mercury speciation in prenatal exposure in Slovenian and Croatian population - PHIME study. Environ Res. 2019 Oct;177:108627. doi: 10.1016/j.envres.2019.108627. Epub 2019 Aug 5. PMID: 31421448. | Intervention/exposure |
| Tressou J, Buaud B, Simon N, Pasteau S, Guesnet P. Very low inadequate dietary intakes of essential n-3 polyunsaturated fatty acids (PUFA) in pregnant and lactating French women: The INCA2 survey. Prostaglandins Leukot Essent Fatty Acids. 2019 Jan;140:3-10. doi: 10.1016/j.plefa.2018.11.007. Epub 2018 Nov 22. PMID: 30553400. | Intervention/exposure |
| Varsi K, Torsvik IK, Huber S, Averina M, Brox J, Bjørke-Monsen AL. Impaired gross motor development in infants with higher PFAS concentrations. Environ Res. 2022 Mar;204(Pt D):112392. doi: 10.1016/j.envres.2021.112392. Epub 2021 Nov 17. PMID: 34800539. | Population |
| Vecchione R, Vigna C, Whitman C, Kauffman EM, Braun JM, Chen A, Xu Y, Hamra GB, Lanphear BP, Yolton K, Croen LA, Fallin MD, Irva Hertz-Picciotto, Newschaffer CJ, Lyall K. The Association Between Maternal Prenatal Fish Intake and Child Autism-Related Traits in the EARLI and HOME Studies. J Autism Dev Disord. 2021 Feb;51(2):487-500. doi: 10.1007/s10803-020-04546-9. PMID: 32519188; PMCID: PMC7725860. | Population |
| Vejrup K, Brantsæter AL, Meltzer HM, Mohebbi M, Knutsen HK, Alexander J, Haugen M, Jacka F. Prenatal mercury exposure, fish intake and child emotional behavioural regulation in the Norwegian Mother, Father and Child Cohort Study. BMJ Nutr Prev Health. 2022 Nov 15;5(2):313-320. doi: 10.1136/bmjnph-2021-000412. PMID: 36619316; PMCID: PMC9813626. | Outcome |
| Wang J, Wu W, Li H, Cao L, Wu M, Liu J, Gao Z, Zhou C, Liu J, Yan C. Relation of prenatal low-level mercury exposure with early child neurobehavioral development and exploration of the effects of sex and DHA on it. Environ Int. 2019 May;126:14-23. doi: 10.1016/j.envint.2019.02.012. Epub 2019 Feb 15. PMID: 30776746. | Intervention/exposure |
| Wang S, Dong D, Li P, Hua X, Zheng N, Sun S, Hou S, An Q, Li P, Li Y, Song X, Li X. Mercury concentration and fatty acid composition in muscle tissue of marine fish species harvested from Liaodong Gulf: An intelligence quotient and coronary heart disease risk assessment. Sci Total Environ. 2020 Jul 15;726:138586. doi: 10.1016/j.scitotenv.2020.138586. Epub 2020 Apr 8. PMID: 32481211. | Population |
| Wernio E, Kłosowska A, Kuchta A, Ćwiklińska A, Sałaga-Zaleska K, Jankowski M, Kłosowski P, Wiśniewski P, Wierzba J, Małgorzewicz S. Analysis of Dietary Habits and Nutritional Status of Children with Down Syndrome in the Context of Lipid and Oxidative Stress Parameters. Nutrients. 2022 Jun 9;14(12):2390. doi: 10.3390/nu14122390. PMID: 35745122; PMCID: PMC9231028. | Intervention/exposure |
| Weyde KVF, Winterton A, Surén P, Andersen GL, Vik T, Biele G, Knutsen HK, Thomsen C, Meltzer HM, Skogheim TS, Engel SM, Aase H, Villanger GD. Association between gestational levels of toxic metals and essential elements and cerebral palsy in children. Front Neurol. 2023 Aug 17;14:1124943. doi: 10.3389/fneur.2023.1124943. PMID: 37662050; PMCID: PMC10470125. | Intervention/exposure |
| Yawei S, Jianhai L, Junxiu Z, Xiaobo P, Zewu Q. Epidemiology, clinical presentation, treatment, and follow-up of chronic mercury poisoning in China: a retrospective analysis. BMC Pharmacol Toxicol. 2021 May 3;22(1):25. doi: 10.1186/s40360-021-00493-y. PMID: 33941274; PMCID: PMC8091676. | Intervention/exposure |
| Young EC, Davidson PW, Wilding G, Myers GJ, Shamlaye C, Cox C, de Broeck J, Bennett CM, Reeves JS. Association between prenatal dietary methyl mercury exposure and developmental outcomes on acquisition of articulatory-phonologic skills in children in the Republic of Seychelles. Neurotoxicology. 2020 Dec;81:353-357. doi: 10.1016/j.neuro.2020.09.028. Epub 2020 Oct 14. PMID: 33741116. | Intervention/exposure |
| Zareba W, Thurston SW, Zareba G, Couderc JP, Evans K, Xia J, Watson GE, Strain JJ, McSorley E, Yeates A, Mulhern M, Shamlaye CF, Bovet P, van Wijngaarden E, Davidson PW, Myers GJ. Prenatal and recent methylmercury exposure and heart rate variability in young adults: the Seychelles Child Development Study. Neurotoxicol Teratol. 2019 Jul-Aug;74:106810. doi: 10.1016/j.ntt.2019.106810. Epub 2019 May 23. PMID: 31128243; PMCID: PMC6642841. | Population |

*Only one reason for exclusion is required; therefore, there may be additional reasons for exclusion that are not listed .

### **Supplementary Table 5**: Risk of bias for studies that reported at least one result related to seafood consumption during childhood and adolescence and cognitive development

Randomized controlled trials assessed with ROB 2.0:

| **Article** | **Overall risk of bias judgement** | **Risk of bias arising from the randomization process** | **Risk of bias due to deviations from the intended interventions** | **Risk of bias due to missing outcome data** | **Risk of bias in measurement of the outcome** | **Risk of bias in selection of the reported result** |
| --- | --- | --- | --- | --- | --- | --- |
| Unnamed randomized  controlled trial in Germany  Demmelmair, 2019 [1] | Some Concerns | Some Concerns | Low Risk | Low Risk | Low Risk | Low Risk |
| Fish Intervention Studies - TEENS  (FINS-TEENS)  Handeland, 2017 [2] | Low Risk | Low Risk | Low Risk | Low Risk | Low Risk | Low Risk |
| Polyunsaturated fatty  acids in child nutrition  (PINGU)  Kalhoff, 2020 [3] | High Risk | Some Concerns | Some Concerns | Some Concerns | Low Risk | High Risk |
| Fish Intervention Studies – KIDS (FIN-KIDS)  Kvestad, 2018 [4] | Some Concerns | Low Risk | Some Concerns | Low Risk | Low Risk | Low Risk |
| Fish Intervention Studies – KIDS (FIN-KIDS)  Oyen, 2018 [5] | Low Risk | Low Risk | Low Risk | Low Risk | Low Risk | Low Risk |
| FiSK Junior (Fish, children, health, and cognition)  Teisen, 2020 [6] | Low Risk | Low Risk | Low Risk | Low Risk | Low Risk | Low Risk |

Cohort studies assessed with ROBINS-E:

| **Article** | **Overall risk of bias judgement** | **Risk of bias due to confounding** | **Risk of bias arising from measurement of the exposure** | **Risk of bias in selecting participants into the study (or analysis)** | **Risk of bias due to post-exposure interventions** | **Risk of bias due to missing data** | **Risk of bias arising from measurement of the outcome** | **Risk of bias in selection of the reported result** |
| --- | --- | --- | --- | --- | --- | --- | --- | --- |
| Avon Longitudinal Study of Parents and Children (ALSPAC)  Williams, 2001 [16] | High Risk | High Risk | Some Concerns | Low Risk | Low Risk | Some Concerns | Low Risk | High Risk |
| Spanish Environment and Childhood Project (INMA)  Julvez, 2020 [14] | High Risk | Some Concerns | Low Risk | Low Risk | Some Concerns | Some Concerns | Low Risk | High Risk |
| China Jintan Child Cohort Study  Liu, 2017 [9] | Some Concerns | Some Concerns | Some Concerns | Low Risk | Low Risk | Some Concerns | Low Risk | Low Risk |
| ALLERGY 2000  Kim, 2010 [8] | Some Concerns | Some Concerns | Some Concerns | Low Risk | Low Risk | Some Concerns | Low Risk | Low Risk |
| Unnamed prospective cohort study in Sweden  Aberg, 2009 [7] | High Risk | Some Concerns | High Risk | Low Risk | Some Concerns | Some Concerns | Low Risk | High Risk |

### **Supplementary Table 6**: Risk of bias for studies that reported at least one result related to seafood consumption during childhood and adolescence and behavior, inclusive of social-emotional and behavioral development

Randomized controlled trials assessed with ROB 2.0:

| **Article** | **Overall risk of bias judgement** | **Risk of bias arising from the randomization process** | **Risk of bias due to deviations from the intended interventions** | **Risk of bias due to missing outcome data** | **Risk of bias in measurement of the outcome** | **Risk of bias in selection of the reported result** |
| --- | --- | --- | --- | --- | --- | --- |
| Fish Intervention Studies - KIDS (FINS-KIDS)  Hysing, 2018 [10] | Some Concerns | Low Risk | Low Risk | Some Concerns | Some Concerns | Some Concerns |
| Fish Intervention Studies - TEENS (FINS-TEENS)  Skotheim, 2017 [11] | Some Concerns | Low Risk | Low Risk | Low Risk | Low Risk | Some Concerns |
| FiSK Junior study (Fish, children, health, and cognition)  Teisen, 2020 [6] | Low Risk | Low Risk | Low Risk | Low Risk | Low Risk | Low Risk |

Cohort studies assessed with ROBINS-E:

| **Article** | **Overall risk of bias judgement** | **Risk of bias due to confounding** | **Risk of bias arising from measurement of the exposure** | **Risk of bias in selecting participants into the study (or analysis)** | **Risk of bias due to post-exposure interventions** | **Risk of bias due to missing data** | **Risk of bias arising from measurement of the outcome** | **Risk of bias in selection of the reported result** |
| --- | --- | --- | --- | --- | --- | --- | --- | --- |
| Community Empowerment  and Care for Wellbeing  and Health Longevity  Ajmal, 2022 [12] | High Risk | High Risk | Some Concerns | Low Risk | Low Risk | High Risk | Low Risk | High Risk |
| Avon Longitudinal Study  of Parents and Children (ALSPAC)  Mesirow, 2017 [13] | High Risk | Some Concerns | Low Risk | Low Risk | Some Concerns | Some Concerns | Low Risk | High Risk |

### **Supplementary Table 7**: Risk of bias for studies that reported at least one result related to seafood consumption during childhood and adolescence and movement/physical development

Randomized controlled trials assessed with ROB 2.0:

| **Article** | **Overall risk of bias judgement** | **Risk of bias arising from the randomization process** | **Risk of bias due to deviations from the intended interventions** | **Risk of bias due to missing outcome data** | **Risk of bias in measurement of the outcome** | **Risk of bias in selection of the reported result** |
| --- | --- | --- | --- | --- | --- | --- |
| Unnamed randomized controlled trial in Germany  Demmelmair, 2019 [1] | Some Concerns | Some Concerns | Low Risk | Low Risk | Low Risk | Low Risk |
| Polyunsaturated fatty acids in child nutrition (PINGU)  Kalhoff, 2020 [3] | High Risk | Some Concerns | Some Concerns | Some Concerns | Low Risk | High Risk |
| Fish Intervention Studies - KIDS (FINS-KIDS)  Oyen, 2018 [5] | Low Risk | Low Risk | Low Risk | Low Risk | Low Risk | Low Risk |

### **Supplementary Table 8**: Risk of bias for studies that reported at least one result related to seafood consumption during childhood and adolescence and language/communication development

Cohort studies assessed with ROBINS-E:

| **Article** | **Overall risk of bias judgement** | **Risk of bias due to confounding** | **Risk of bias arising from measurement of the exposure** | **Risk of bias in selecting participants into the study (or analysis)** | **Risk of bias due to post-exposure interventions** | **Risk of bias due to missing data** | **Risk of bias arising from measurement of the outcome** | **Risk of bias in selection of the reported result** |
| --- | --- | --- | --- | --- | --- | --- | --- | --- |
| Odense Child Cohort  Beck, 2023 [15] | High Risk | High Risk | High Risk | Some Concerns | Low Risk | Some Concerns | Low Risk | Low Risk |

### **Supplementary Table 9**: Risk of bias for studies that reported at least one result related to seafood consumption during childhood and adolescence and depression or anxiety

Cohort studies assessed with ROBINS-E:

| **Article** | **Overall risk of bias judgement** | **Risk of bias due to confounding** | **Risk of bias arising from measurement of the exposure** | **Risk of bias in selecting participants into the study (or analysis)** | **Risk of bias due to post-exposure interventions** | **Risk of bias due to missing data** | **Risk of bias arising from measurement of the outcome** | **Risk of bias in selection of the reported result** |
| --- | --- | --- | --- | --- | --- | --- | --- | --- |
| Children's Lifestyle and School Performance Study (CLASS)  McMartin, 2012 [17] | Some Concerns | Some Concerns | Low Risk | Low Risk | Low Risk | Some Concerns | Low Risk | Low Risk |
| ROOTS Study  Winpenny, 2018 [18] | Some Concerns | Some Concerns | Low Risk | Some Concerns | Some Concerns | Some Concerns | Low Risk | Low Risk |

### **Supplementary Appendix 1:** PRISMA checklist

| **Section and Topic** | **Item #** | **Checklist item** | **Location where item is reported** |
| --- | --- | --- | --- |
| **TITLE** | | |  |
| Title | 1 | Identify the report as a systematic review. | Title |
| **ABSTRACT** | | |  |
| Abstract | 2 | See the PRISMA 2020 for Abstracts checklist. | Used journal formatting |
| **INTRODUCTION** | | |  |
| Rationale | 3 | Describe the rationale for the review in the context of existing knowledge. | Introduction |
| Objectives | 4 | Provide an explicit statement of the objective(s) or question(s) the review addresses. | Last statement in introduction and Supplementary Figure 1 |
| **METHODS** | | |  |
| Eligibility criteria | 5 | Specify the inclusion and exclusion criteria for the review and how studies were grouped for the syntheses. | Second paragraph in Methods and first sentence in Data Synthesis |
| Information sources | 6 | Specify all databases, registers, websites, organisations, reference lists and other sources searched or consulted to identify studies. Specify the date when each source was last searched or consulted. | Search strategy and Supplementary Table 2 |
| Search strategy | 7 | Present the full search strategies for all databases, registers and websites, including any filters and limits used. | Supplementary Table 2 |
| Selection process | 8 | Specify the methods used to decide whether a study met the inclusion criteria of the review, including how many reviewers screened each record and each report retrieved, whether they worked independently, and if applicable, details of automation tools used in the process. | Screening |
| Data collection process | 9 | Specify the methods used to collect data from reports, including how many reviewers collected data from each report, whether they worked independently, any processes for obtaining or confirming data from study investigators, and if applicable, details of automation tools used in the process. | Data extraction |
| Data items | 10a | List and define all outcomes for which data were sought. Specify whether all results that were compatible with each outcome domain in each study were sought (e.g. for all measures, time points, analyses), and if not, the methods used to decide which results to collect. | Last sentence of the second paragraph of Methods |
|  | 10b | List and define all other variables for which data were sought (e.g. participant and intervention characteristics, funding sources). Describe any assumptions made about any missing or unclear information. | Data extraction |
| Study risk of bias assessment | 11 | Specify the methods used to assess risk of bias in the included studies, including details of the tool(s) used, how many reviewers assessed each study and whether they worked independently, and if applicable, details of automation tools used in the process. | Risk of bias |
| Effect measures | 12 | Specify for each outcome the effect measure(s) (e.g. risk ratio, mean difference) used in the synthesis or presentation of results. | Data synthesis indicates both effects and associations were considered. |
| Synthesis methods | 13a | Describe the processes used to decide which studies were eligible for each synthesis (e.g. tabulating the study intervention characteristics and comparing against the planned groups for each synthesis (item #5)). | Data synthesis (i.e., study design and outcome defined in prior sections). |
|  | 13b | Describe any methods required to prepare the data for presentation or synthesis, such as handling of missing summary statistics, or data conversions. | Not relevant for narrative synthesis; data summarized as reported in primary studies. |
|  | 13c | Describe any methods used to tabulate or visually display results of individual studies and syntheses. | Data synthesis: “Study characteristics and outcome data are presented in tabular format.” |
|  | 13d | Describe any methods used to synthesize results and provide a rationale for the choice(s). If meta-analysis was performed, describe the model(s), method(s) to identify the presence and extent of statistical heterogeneity, and software package(s) used. | Data synthesis |
|  | 13e | Describe any methods used to explore possible causes of heterogeneity among study results (e.g. subgroup analysis, meta-regression). | Data synthesis: grouped by outcome, study design, and age group. Seafood type and population characteristics also considered in synthesis. |
|  | 13f | Describe any sensitivity analyses conducted to assess robustness of the synthesized results. | Data synthesis: “Sensitivity analyses were conducted by omitting studies that were at high or very high risk of bias.” |
| Reporting bias assessment | 14 | Describe any methods used to assess risk of bias due to missing results in a synthesis (arising from reporting biases). | Risk of bias: ROBINS-I, ROBINS-E, and ROB 2.0 include a domain for missing data and reporting bias. |
| Certainty assessment | 15 | Describe any methods used to assess certainty (or confidence) in the body of evidence for an outcome. | Certainty of evidence |
| **RESULTS** | | |  |
| Study selection | 16a | Describe the results of the search and selection process, from the number of records identified in the search to the number of studies included in the review, ideally using a flow diagram. | Search results and Supplementary Figure 2 |
|  | 16b | Cite studies that might appear to meet the inclusion criteria, but which were excluded, and explain why they were excluded. | Supplementary Table 4 |
| Study characteristics | 17 | Cite each included study and present its characteristics. | Citations are provided within each outcome section and characteristics of all studies are shown in Table 1. |
| Risk of bias in studies | 18 | Present assessments of risk of bias for each included study. | Risk of bias is described for each study with citations within each outcome section and all results are presented in tabular format in Supplementary Tables 5-9. |
| Results of individual studies | 19 | For all outcomes, present, for each study: (a) summary statistics for each group (where appropriate) and (b) an effect estimate and its precision (e.g. confidence/credible interval), ideally using structured tables or plots. | Tables 2, 4, and 5 |
| Results of syntheses | 20a | For each synthesis, briefly summarise the characteristics and risk of bias among contributing studies. | Described within each outcome section. |
|  | 20b | Present results of all statistical syntheses conducted. If meta-analysis was done, present for each the summary estimate and its precision (e.g. confidence/credible interval) and measures of statistical heterogeneity. If comparing groups, describe the direction of the effect. | No statistical syntheses or meta-analyses conducted, thus this is not relevant. |
|  | 20c | Present results of all investigations of possible causes of heterogeneity among study results. | No statistical syntheses or meta-analyses conducted, but source of heterogeneity such as seafood type and population characteristics are described throughout the outcome sections. |
|  | 20d | Present results of all sensitivity analyses conducted to assess the robustness of the synthesized results. | Described at the end of each outcome section. |
| Reporting biases | 21 | Present assessments of risk of bias due to missing results (arising from reporting biases) for each synthesis assessed. | ROBINS-I, ROBINS-E, and ROB 2.0 include a domain for missing data and reporting bias and are described for each study with citations within each outcome section and all results are presented in tabular format in Supplementary Tables 5-9. |
| Certainty of evidence | 22 | Present assessments of certainty (or confidence) in the body of evidence for each outcome assessed. | Table 3 |
| **DISCUSSION** | | |  |
| Discussion | 23a | Provide a general interpretation of the results in the context of other evidence. | First two paragraphs of the Discussion. |
|  | 23b | Discuss any limitations of the evidence included in the review. | Third and fourth paragraphs of Discussion. |
|  | 23c | Discuss any limitations of the review processes used. | Sixth paragraph in the Discussion. |
|  | 23d | Discuss implications of the results for practice, policy, and future research. | Fourth and fifth paragraphs in the Discussion. |
| **OTHER INFORMATION** | | |  |
| Registration and protocol | 24a | Provide registration information for the review, including register name and registration number, or state that the review was not registered. | First paragraph in Methods. |
|  | 24b | Indicate where the review protocol can be accessed, or state that a protocol was not prepared. | First paragraph in Methods. |
|  | 24c | Describe and explain any amendments to information provided at registration or in the protocol. | Data synthesis: “Meta-analyses were planned, as indicated in the protocol, but not performed due to limitations in the data. This was a deviation from the protocol.” |
| Support | 25 | Describe sources of financial or non-financial support for the review, and the role of the funders or sponsors in the review. | Funding |
| Competing interests | 26 | Declare any competing interests of review authors. | Author disclosures |
| Availability of data, code and other materials | 27 | Report which of the following are publicly available and where they can be found: template data collection forms; data extracted from included studies; data used for all analyses; analytic code; any other materials used in the review. | Data availability and Supplemental Data Appendix |

### **Supplementary Appendix 2:** AMSTAR 2 checklist assessing methodological quality of the systematic review

(***bolded and italicized text*** indicates which criteria were met and the far-right columns indicates the rationale and location in the manuscript)

| 1. **Did the research questions and inclusion criteria for the review include the components of PICO?** | | | |
| --- | --- | --- | --- |
| **For yes:**   - ***Population*** - ***Intervention*** - ***Comparator group*** - ***Outcome*** | **Optional (recommended):**   - Timeframe for follow-up | **Final rating:**   - ***Yes*** - No | **Location:**  Second paragraph of Methods and Analytic Framework: “In brief, randomized controlled trials (RCTs), non-randomized intervention studies, prospective cohort studies (PCSs), and retrospective cohort studies that compared different types, amounts, sources, frequency, or timing of seafood consumption during childhood and adolescence and neurocognitive development outcomes in the child at ages 0-18 years old were eligible (Supplementary Figure 1).” |
| 1. **Did the report of the review contain an explicit statement that the review methods were established prior to the conduct of the review and did the report justify any significant deviations from the protocol?** | | | |
| **For partial yes:**  **The authors state that they had a written protocol or guide that included ALL the following**   - ***Review question(s)*** - ***A search strategy*** - ***Inclusion/exclusion criteria*** - ***A risk of bias assessment*** | **For yes:**  **As for partial yes, plus the protocol should be registered and should also have specified:**   - ***A meta-analysis/synthesis plan, if appropriate, and*** - ***A plan for investigating causes of heterogeneity*** - ***Justification for any deviations from the protocol*** | **Final rating:**   - ***Yes*** - Partial yes - No | **Location:**  First paragraph of Methods: Our protocol was registered a priori in PROSPERO (CRD42023432844) and was based on the 2020 DGAC protocol [12]. The protocol included the review questions, general search strategy, inclusion/exclusion criteria, risk of bias assessment, and synthesis plan including heterogeneity investigation.  Justification for the deviation from the protocol is in Data synthesis: “Meta-analyses were planned, as indicated in the protocol, but not performed due to limitations in the data. This was a deviation from the protocol. This decision was made because only a small portion of the total extracted data could be pooled (e.g., 8 out of the 98 cognitive development outcomes and 10 of 32 behavior outcomes) due to the variation in the population age, outcome assessment tools, sub-outcomes and the variety of estimands reported.” |
| 1. **Did the review authors explain their selection of the study designs for inclusion in the review?** | | | |
| **For yes, the review should satisfy ONE of the following:**   - *Explanation for* including only RCTs - OR *Explanation for* including only NRSI - ***OR Explanation for including both RCTs and NRSI*** | | **Final rating:**   - ***Yes*** - No | **Location:**  Second paragraph in Methods: “These study designs and outcomes were selected to match the 2020 DGAC protocol that was developed by technical experts in the field and systematic review methodologists.” |
| 1. **Did the review authors use a comprehensive literature strategy?** | | | |
| **For partial yes (all the following):**   - ***Searched at least 2 databases (relevant to research question)*** - ***Provided key word and/or search strategy*** - ***Justified publication restrictions (e.g., language)*** | **For yes, should also have (all the following):**   - ***Searched the reference lists/bibliographies of included studies*** - ***Searched trial/study registries*** - ***Included/consulted content experts in the field*** - ***Where relevant, searched for grey literature*** - ***Conducted search within 24 months of completion of the review*** | **Final rating:**   - ***Yes*** - Partial yes - No | **Location:**  Search strategy and Supplemental Table 2. At least one database states that they include trial and study registries (e.g., Embase and CENTRAL). |
| 1. **Did the review authors perform study selection in duplicate?** | | | |
| **For yes, either ONE of the following:**   - ***at least two reviewers independently agreed on selection of eligible studies and archived consensus on which studies to include*** - OR two reviewers selected a sample of eligible studies and achieved good agreement (at least 80%), with the remainder selected by one reviewer | | **Final rating:**   - ***Yes*** - No | **Location:**  Screening |
| 1. **Did the review authors perform data extraction in duplicate?** | | | |
| **For yes, either ONE of the following:**   - ***At least two reviewers achieved consensus on which data to extract from included studies*** - OR two reviewers extracted data from a sample of eligible studies and achieved good agreement (at least 80%), with the remainder extracted by one reviewer | | **Final rating:**   - ***Yes*** - No | **Location:**  Data extraction: “Data from all articles were extracted by a trained analyst using a systematic approach and a standardized data extraction form. A second analyst reviewed all extracted data for accuracy and completeness.” |
| 1. **Did the review authors provide a list of excluded studies and justify the exclusions?** | | | |
| **For partial yes:**   - ***Provided a list of all potentially relevant studies that were read in full text form but excluded from review*** | **For yes, must also have:**   - ***Justified the exclusion from the review of each potentially relevant study*** | **Final rating:**   - ***Yes*** - Partial yes - No | **Location:**  Supplementary Table 4 |
| 1. **Did the review authors describe the included studies in adequate detail?** | | | |
| **For partial yes (all the following):**   - ***Described populations*** - ***Described interventions*** - ***Described comparators*** - ***Described outcomes*** - ***Described research designs*** | **For yes, should also have ALL the following:**   - ***Described population in detail*** - ***Described intervention and comparator in details (including doses where relevant)*** - ***Described study’s setting*** - ***Timeframe for follow-up*** | **Final rating:**   - ***Yes*** - Partial yes - No | **Location:**  Tables 1, 2, 4, and 5 |
| 1. **Did the review authors use a satisfactory technique for assessing the risk of bias (RoB) in individual studies that were included in the review?** | | | |
| **RCTs** | | | |
| **For partial yes, must have assessed RoB from:**   - ***Unconcealed allocation, and*** - ***Lack of blinding of patients and assessors when assessing outcomes (unnecessary for objective outcomes such as all-cause mortality)*** | **For yes, must also have assessed RoB from:**   - ***Allocation sequence that was not truly random, and*** - ***Selection of the reported result from among multiple measurements or analyses of a specified outcome*** | **Final rating:**   - ***Yes*** - Partial yes - No - Includes only NRSI | **Location:**  Risk of bias: ROB 2.0 was used for RCTs which includes these domains. |
| **NRSI** | | | |
| **For partial yes, must have assessed RoB from:**   - ***Confounding, and*** - ***Selection bias*** | **For yes, must also have assessed RoB:**   - ***Methods used to ascertain exposures and outcomes, and*** - ***Selection of the reported result from among multiple measurements or analyses of a specified outcome*** | **Final rating:**   - ***Yes*** - Partial yes - No - Includes only RCTs | **Location:**  Risk of bias: ROBINS-E was used for NRSI which includes these domains. |
| 1. **Did the review authors report on the sources of funding for the studies included in the review?** | | | |
| **For yes:**   - ***Must have reported on the sources of funding for individual studies included in the review. Note: Reporting that the reviewers look for this information, but it was not reported by study authors also qualifies*** | | **Final rating:**   - ***Yes*** - No | **Location:**  Table 1 |
| 1. **If meta-analysis was performed did the review authors use appropriate methods for statistical combination of results?** | | | |
| **RCTs** | | | |
| **For yes:**   - The authors justified combining the data in a meta-analysis - AND they used an appropriate weighted technique to combine study results and adjusted for heterogeneity if present - AND investigated the causes of any heterogeneity | | **Final rating:**   - Yes - No - ***No meta-analysis conducted*** | **Location:**  Not applicable. |
| **NRSI** | | | |
| **For yes:**   - The authors justified combining the data in a meta-analysis - AND they used an appropriate weighted technique to combine study results, adjusting for heterogeneity if present - AND they statistically combined effect estimates from NRSI that were adjusted for confounding, rather than combining raw rata, or justified combining raw data when adjusted effect estimates were not available - AND they reported separate summary estimates for RCTs and NRSI separately when both were included in the review | | **Final rating:**   - Yes - No - ***No meta-analysis conducted*** | **Location:**  Not applicable. |
| 1. **If meta-analysis was performed, did the review authors assess the potential impact of RoB in individual studies on the results of the meta-analysis or other evidence synthesis?** | | | |
| **For yes:**   - Included only low risk of bias RCTs - OR, if the pooled estimate was based on RCTs and/or NRSI at variable RoB, the authors performed analyses to investigate possible impact of RoB on summary estimates of effect | | **Final rating:**   - Yes - No - ***No meta-analysis conducted*** | **Location:**  Not applicable. |
| 1. **Did the review authors account for RoB in individual studies when interpreting/discussing the results of the review?** | | | |
| **For yes:**   - Included only low risk of bias RCTs - ***OR, if RCTs with moderate or high RoB, or NRSI were included the review provided a discussion with the likely impact of RoB on the results*** | | **Final rating:**   - ***Yes*** - No | **Location:**  The risk of bias was described for each study within each outcome section in the results and sensitivity analyses excluding studies at high risk of bias were performed. |
| 1. **Did the review authors provide a satisfactory explanation for, and discussion of, any heterogeneity observed in the results of the review?** | | | |
| **For yes:**   - There was no significant heterogeneity in the results - ***OR if heterogeneity was present the authors performed an investigation of sources of any heterogeneity in the results and discussed the impact of this on the results of the review*** | | **Final rating:**   - ***Yes*** - No | **Location:**  Sources of heterogeneity, such as seafood type or population characteristics, are described throughout the results section for each outcome to aid in the narrative synthesis and also described in the third and fourth paragraph of Discussion. |
| 1. **If they performed quantitative synthesis did the review authors carry out an adequate investigation of publication bias (small study bias) and discuss its likely impact on the results of the review?** | | | |
| **For yes:**   - Performed graphical or statistical tests for publication bias and discussed the likelihood and magnitude of impact of publication bias | | **Final rating:**   - Yes - No - ***No meta-analysis conducted*** | **Location:**  Not applicable. |
| 1. **Did the review authors report any potential sources of conflict of interest, including any funding they received for conducting the review?** | | | |
| **For yes:**   - The authors reported no competing interests OR - ***The authors described their funding sources and how they managed potential conflicts of interest*** | | **Final rating:**   - ***Yes*** - No | **Location:**  Author disclosures |

From Shea BJ, Reeves BC, Wells G, et al. Amstar 2: A critical appraisal tool for systematic reviews that include randomised or non-randomised studies of healthcare interventions, or both. *BMJ*. Sep 21 2017;358:j4008. doi:10.1136/bmj.j4008

### Supplementary references

1. Demmelmair H, Oyen J, Pickert T, et al. The effect of Atlantic salmon consumption on the cognitive performance of preschool children - A randomized controlled trial. Clin Nutr. Dec 2019;38(6):2558-2568. doi:10.1016/j.clnu.2018.11.031

2. Handeland K, Oyen J, Skotheim S, et al. Fatty fish intake and attention performance in 14-15 year old adolescents: FINS-TEENS - a randomized controlled trial. Nutr J. Oct 2 2017;16(1):64. doi:10.1186/s12937-017-0287-9

3. Kalhoff H, Mesch CM, Stimming M, et al. Effects of LC-PUFA supply via complementary food on infant development-a food based intervention (RCT) embedded in a total diet concept. Eur J Clin Nutr. May 2020;74(5):682-690. doi:10.1038/s41430-019-0491-0

4. Kvestad I, Vabo S, Kjellevold M, et al. Fatty fish, hair mercury and cognitive function in Norwegian preschool children: Results from the randomized controlled trial FINS-KIDS. *Environ Int*. Dec 2018;121(Pt 2):1098-1105. doi:10.1016/j.envint.2018.10.022

5. Oyen J, Kvestad I, Midtbo LK, et al. Fatty fish intake and cognitive function: FINS-KIDS, a randomized controlled trial in preschool children. *BMC Med*. Mar 12 2018;16(1):41. doi:10.1186/s12916-018-1020-z

6. Teisen MN, Vuholm S, Niclasen J, et al. Effects of oily fish intake on cognitive and socioemotional function in healthy 8-9-year-old children: the FiSK Junior randomized trial. *Am J Clin Nutr*. Jul 1 2020;112(1):74-83. doi:10.1093/ajcn/nqaa050

7. Aberg MA, Aberg N, Brisman J, Sundberg R, Winkvist A, Toren K. Fish intake of Swedish male adolescents is a predictor of cognitive performance. *Acta Paediatr*. Mar 2009;98(3):555-60. doi:10.1111/j.1651-2227.2008.01103.x

8. Kim JL, Winkvist A, Aberg MA, et al. Fish consumption and school grades in Swedish adolescents: a study of the large general population. *Acta Paediatr*. Jan 2010;99(1):72-7. doi:10.1111/j.1651-2227.2009.01545.x

9. Liu J, Cui Y, Li L, et al. The mediating role of sleep in the fish consumption - cognitive functioning relationship: a cohort study. *Sci Rep*. Dec 21 2017;7(1):17961. doi:10.1038/s41598-017-17520-w

10. Hysing M, Kvestad I, Kjellevold M, et al. Fatty Fish Intake and the Effect on Mental Health and Sleep in Preschool Children in FINS-KIDS, a Randomized Controlled Trial. *Nutrients*. Oct 11 2018;10(10)doi:10.3390/nu10101478

11. Skotheim S, Handeland K, Kjellevold M, et al. The effect of school meals with fatty fish on adolescents' self-reported symptoms for mental health: FINS-TEENS - a randomized controlled intervention trial. *Food Nutr Res*. 2017;61(1):1383818. doi:10.1080/16546628.2017.1383818

12. Ajmal A, Watanabe K, Tanaka E, et al. Eating Behaviour-Consumption Frequency of Certain Foods in Early Childhood as a Predictor of Behaviour Problems: 6-year follow-up study. *Sultan Qaboos Univ Med J*. May 2022;22(2):225-232. doi:10.18295/squmj.5.2021.103

13. Mesirow MS, Cecil C, Maughan B, Barker ED. Associations between Prenatal and Early Childhood Fish and Processed Food Intake, Conduct Problems, and Co-Occurring Difficulties. *J Abnorm Child Psychol*. Jul 2017;45(5):1039-1049. doi:10.1007/s10802-016-0224-y

14. Julvez J, Fernandez-Barres S, Gignac F, et al. Maternal seafood consumption during pregnancy and child attention outcomes: a cohort study with gene effect modification by PUFA-related genes. *Int J Epidemiol*. Apr 1 2020;49(2):559-571. doi:10.1093/ije/dyz197

15. Beck IH, Bilenberg N, Moller S, et al. Association Between Prenatal and Early Postnatal Exposure to Perfluoroalkyl Substances and IQ Score in 7-Year-Old Children From the Odense Child Cohort. *Am J Epidemiol*. Sep 1 2023;192(9):1522-1535. doi:10.1093/aje/kwad110

16. Williams C, Birch EE, Emmett PM, Northstone K, Avon Longitudinal Study of P, Childhood Study T. Stereoacuity at age 3.5 y in children born full-term is associated with prenatal and postnatal dietary factors: a report from a population-based cohort study. *Am J Clin Nutr*. Feb 2001;73(2):316-22. doi:10.1093/ajcn/73.2.316

17. McMartin SE, Kuhle S, Colman I, Kirk SF, Veugelers PJ. Diet quality and mental health in subsequent years among Canadian youth. *Public Health Nutr*. Dec 2012;15(12):2253-8. doi:10.1017/S1368980012000535

18. Winpenny EM, van Harmelen AL, White M, van Sluijs EM, Goodyer IM. Diet quality and depressive symptoms in adolescence: no cross-sectional or prospective associations following adjustment for covariates. *Public Health Nutr*. Sep 2018;21(13):2376-2384. doi:10.1017/S1368980018001179
